# Achieving near-theoretical strength and high elasticity in micrometer scale TiB_2_ ceramics

**DOI:** 10.1038/s41467-026-74750-1

**Published:** 2026-07-01

**Authors:** Qianduo Zhuang, Yizhuang Li, Fanghai Xin, Mingxin Huang, Wei Xu

**Affiliations:** 1 State Key Laboratory of Digital Steel, Northeastern University, Shenyang, China; 2 K.H Kuo Center for Material Characterization, Liaoning Academy of Materials, Shenyang, China; 3 Department of Mechanical Engineering, The University of Hong Kong, Hong Kong, China

**Keywords:** Actuators, Ceramics

## Abstract

Achieving near-theoretical strength and elastic limits in crystalline solids remains challenging, yet defect sensitivity typically restricts such behavior to nanometer scale specimens. Here we report experimental evidence of near-theoretical strength and large elastic tensile strains in micrometer scale TiB_2_ ceramics, produced in situ by eutectic solidification in steel. In situ bending of crystallographically oriented cantilevers, fixed end beams and C-shaped structures, combined with specimen specific finite element analysis, reveals tensile side stresses of tens of gigapascals and elastic strains up to 9%. Direct microscale tension shows that a large gauge volume exceeding 4 μm^3^ sustains nearly uniform tensile stress of ~13 GPa without fracture before grip edge failure, providing a conservative lower bound tensile benchmark, while micropillar compression confirms high stress bearing capability. These results establish eutectic solidification as a scalable pathway to suppress strength limiting defects over micrometer scale volumes, extending near-theoretical ceramic strength beyond the nanoscale and enabling robust microarchitected components.

## Abstract (web summary)

Achieving near theoretical strength at micrometer sizes remains challenging. Here, the authors use eutectic solidification in steel to grow micrometer scale TiB_2_ ceramics that reach tens of gigapascals and elastically stretch up to 9%.

## Introduction

The pursuit of the theoretical strength limit (~E/10, where E is Young’s modulus) of crystalline solids remains a fundamental goal in materials science and solid mechanics^1^. Achieving this limit would enable the development of ultrahigh-performance materials with exceptional durability and resistance to mechanical failure, critical for structures facing extreme localized stresses. However, this intrinsic limit is unattainable in bulk materials due to their high density of large internal defects, which concentrate stresses and induce premature failure^2^. Consequently, near-theoretical strength has only been possible in nanoscale specimens^3^, where minimized defects enable failure governed by intrinsic atomic bonding limits, exemplifying the well-known “smaller is stronger” size effect^4^. Advances in nanofabrication now permit the production and mechanical testing of such extremely small specimens^5^, confirming strengths approaching E/10.

Notable examples include alumina nanoscale whiskers (~E/10)^6^, silicon nanowires (~E/7)^7^, silicon nanopillars (~E/16)^8^, carbon nanotubes (~E/10)^9^, graphene (~E/9)^10–13^, and diamond nanostructures (~E/10)^14–16^. These nanoscale specimens offer the opportunity to observe ultrahigh strength and large elastic strains close to the theoretical limit through nanomechanical testing, primarily via bending or tension. For instance, high-quality chemical vapor deposited diamond^14–16^ has been fabricated into nanoneedles with nanoscale cross sections and tested by bending, yet the stressed volume remains nanoscale even when the overall specimen length is micrometric. Uniaxial tensile tests^16^ on similarly prepared diamond also confine the gauge cross-section to the nanometer scale. Clearly, achieving such near-theoretical properties in truly microscale specimens with micrometer-scale cross sections remains exceptionally challenging. This raises a critical question: can defect populations (voids, cracks) be controlled without relying on extreme size reduction? Resolving this question is of paramount importance, as nanoscale properties are notoriously difficult to scale up, yet micromachined structures—from microsensors^17^ and actuators^18,19^ to precision medical devices and microrobotics^20^—require enhanced mechanical properties at proper geometric dimensions to ensure reliable and durable performance.

In this study, we report the first experimental evidence of near-theoretical strength in micrometer-sized ceramics, using single-crystalline TiB_2_ formed via eutectic reaction in steel as a model system. This easily synthesized ceramic phase achieves maximum tensile surface stresses at failure exceeding 30 GPa and tensile elastic limits up to 9.0% in large specimens with micrometer scale cross sections under bending, distinct from the commonly reported micron-long nanoneedles, whose cross sections (and thus stressed volumes) remain nanoscale. While comparable tensile strains in cross-section-wise microscale specimens were previously attained only in defect-minimized silicon via advanced wafer technologies (also probed by bending), TiB_2_ combines such strains with markedly higher strength due to its superior elastic modulus. Using in situ micromechanical testing of crystallographically oriented cantilevers, fixed end beams, and C-shaped specimens with specimen-specific finite element analyses, we quantify the intrinsic anisotropic elasticity of TiB_2_ and its tensile surface failure stress, which approaches near-theoretical limits. In addition, microscale uniaxial tensile tests on TiB_2_ specimens show that the gauge sections can sustain up to ~13 GPa of near-uniform tensile stress without detectable cracking. Although final failure occurs at the gripping edge due to unavoidable stress concentration, these tests provide a conservative lower-bound tensile benchmark and support the conclusion that the in situ grown TiB_2_ is effectively free of strength-limiting defects at the micrometer scale. Finally, complementary micropillar compression tests reach nominal peak stresses in the tens of gigapascals, further confirming the ultrahigh stress-bearing capability of micrometer-scale TiB_2_.

## Results and discussion

We synthesized micrometer-scale TiB_2_ via eutectic solidification within Fe-Ti-B-based high-modulus steel systems^21^. In these Ti/B-enriched steels, TiB_2_ forms in situ during casting through a Fe-Ti-B eutectic/near-eutectic (often hypereutectic) solidification sequence, which is provided in Supplementary Note 1. Polished and deep-etched surfaces revealed partially exposed, single-crystalline TiB_2_ particles with hexagonal prism morphology (Supplementary Fig. 1). Because approaching near-theoretical strength at the micrometer scale critically depends on suppressing strength-limiting defects, we designed a hierarchy of test geometries that progressively vary the stress state and specimen volume for robust failure analysis. We first use crystallographically oriented microcantilever bending to quantify the intrinsic anisotropic elasticity and tensile side fracture stress of TiB_2_, with specimen-specific FEM mapping the maximum surface tensile stress/strain at failure. The cantilever results suggest that the ultrahigh stresses attained are consistent with an exceptionally low population of critical defects in in situ grown TiB_2_. We then test this inference using two complementary “defect sensitivity probing” geometries. A microscale four-point bending type configuration generates multiple spatially separated tensile-stress hot spots within a single beam, creating several competing sites where a randomly located critical flaw could trigger premature failure. Additionally, C-shaped structures provide an independent loading geometry, serving as an internal consistency check for the anisotropic elastic parameters extracted from the beams and also enabling cyclic loading-unloading with unobstructed views to probe whether damage accumulates from subcritical flaws. Finally, to probe larger, more uniformly stressed volumes under nominal uniaxial loading, we perform direct tensile tests, with FEM quantifying unavoidable grip edge stress concentrations, and further assess internal flaw populations by serial FIB sectioning of large TiB_2_ particles. Together, these experiments establish a consistent picture in which TiB_2_ failure is governed by the local tensile stress maximum rather than by a weakest link distribution of preexisting critical defects.

**Fig. 1:**
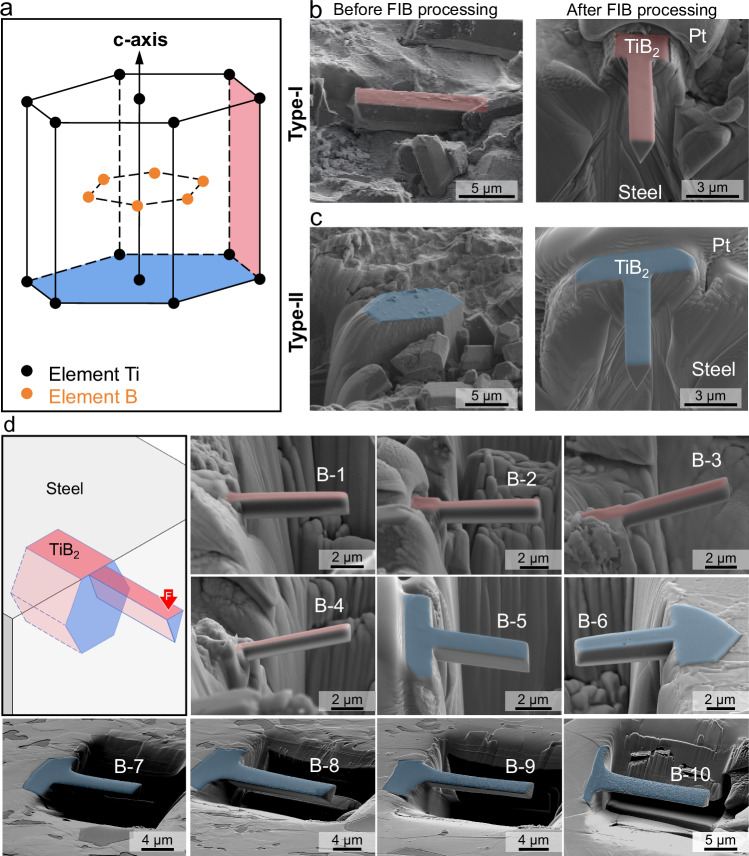
Microcantilever beams with different crystallographic orientations for probing the anisotropic elastic modulus and maximum tensile surface stress at failure of TiB_2_. **a** Schematic of the prismatic and basal planes in single crystal TiB_2_ with a hexagonal crystal structure. **b** Type I and **c** Type Ⅱ microcantilever beams before and after FIB machining. Red and blue indicate the prismatic and basal surfaces of TiB_2_, respectively. **d** Schematic showing a microcantilever beam partially embedded in the matrix for mechanical testing, together with SEM images of representative beams B-1 to B-10. The specimens are arranged from top to bottom and left to right as follows: top row, B-1 to B-3; middle row, B-4 to B-6; bottom row, B-7 to B-10. Different representative beams illustrating near-perfect alignment (B-1, B-2, B-6 to B-10) and slight misalignments (B-3 to B-5).

We first quantitatively assess the anisotropic elastic modulus and fracture strength arising from the hexagonal crystal structure (Fig. 1a) by fabricating ten FIB milled microcantilever beams from individual particles (Fig. 1d) for micromechanical testing. The cantilever geometry (Supplementary Fig. 2) was chosen because it enables tensile stress—essential for evaluating intrinsic fracture strength—unlike the purely compressive stress state in micropillars. Its simple geometry also allows cost-effective FIB fabrication of sufficient specimens for statistical reliability. Beams were oriented with either prismatic planes (type I) or basal planes (type II) nearly parallel to the matrix surface (Fig. 1b, c), with minor misalignments observed in some cases (B-3 and B-4 in Fig. 1d). Partial embedding of TiB_2_ particles within the matrix ensured sufficient clamping at the beam’s fixed end during loading (Fig. 1d). Beam dimensions ranged from 5.1 to 16.8 µm in length, 0.8 to 5.1 µm in width, and 1.1 to 2.6 µm in height (Supplementary Tables 1, 2), targeting the micrometer scale where achieving near-theoretical strength is particularly challenging.

**Fig. 2:**
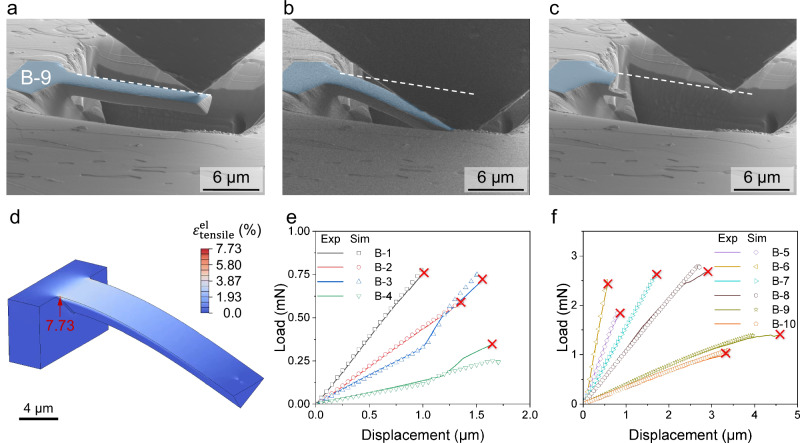
Micromechanical testing of microcantilever beams. **a** Initial state, **b** elastic bending prior to fracture, and **c** final fracture of a representative beam (B-9). The basal planes are marked in blue. **d** FEM simulation of the bending process, reproducing the overall deformation profile and illustrating the distribution of local elastic (tensile) maximum principal strain. Experimentally measured force-displacement curves (solid lines) compared with FEM simulations (symbols) for Type I (**e**) and Type II (**f**) beams, plotted up to the failure point (red cross symbol). For B-3 and B-5, the failure point is defined by the first detectable cracking event at the compressed lower corner; for all other cantilevers, it corresponds to the onset of catastrophic fracture. Source data are provided as a Supplementary Source Data file.

We performed quantitative in situ micromechanical tests on all ten microcantilever beams using an SEM nanoindenter system equipped with flat punch or cube corner tips. Downward loading induced substantial beam deflections, which were fully reversible upon unloading prior to catastrophic fracture (Supplementary Fig. 3). Larger imposed displacements led to fracture of the beams (Fig. 2a–c). The corresponding load-displacement curves, plotted up to the failure point (defined below), for all beams are shown in Fig. 2e, f (with original videos in the Supplementary Movies 1–4). Most beams exhibited linear elastic behavior up to fracture. However, two beams (B-3 and B-4), which displayed upturned initial geometries (Fig. 1d), showed a distinct two-stage elastic response characterized by a change in the slope of their load-displacement curves. This transition occurs when the contact point between the TiB_2_ particle and the flat punch indenter tip shifts toward the indenter edge as the upturned beam ends bend downward, effectively reducing the moment arm. In addition, two beams (B-3 and B-5) showed a small, abrupt load drop shortly before final failure (Supplementary Fig. 4), coincident with the first appearance of a microcrack at the highly constrained lower corner on the compressive side near the fixed end (Supplementary Movies 2, 3). Accordingly, throughout this work we define the failure point used for FEM extraction as the last stable loading increment immediately preceding catastrophic fracture, or, if present, preceding the first detectable cracking event (for B-3 and B-5).

We performed FEM simulations of elastic deformation for each beam, incorporating their actual geometries and corresponding indenter tip shapes. Elastic anisotropy was captured using a unified set of elastic constants (Supplementary Table 3), iteratively optimized to minimize deviations between simulated and experimental prefracture force-displacement curves. The optimized elastic constants—631 GPa along the c-axis and 425 GPa within the basal plane—are consistent with both theoretical predictions^22^ and prior experimental measurements^23^, thereby validating our method for quantifying anisotropy. The excellent agreement between simulations and experiments (Fig. 2e, f) further allows precise determination of the critical stress state at the onset of fracture.

Figure 2d shows the simulated deformation of beam B-9 immediately prior to catastrophic fracture, together with the maximum principal strain distribution. Significant strain concentrations are localized near the fixed end on the upper surface, where a maximum (principal) tensile surface strain ${\varepsilon }_{{{{\rm{I}}}},\max }$ of 7.7% corresponds to a maximum (principal) tensile surface stress at failure of ${\sigma }_{{{{\rm{I}}}},\max }$ = 48.8 GPa. This matches the experimentally observed fracture sites, suggesting that fracture initiates once the local tensile stress reaches the intrinsic strength limit. Under bending, the region above the neutral plane experiences pure tension, while the compressed lower zone resists cracking through the stabilizing effect of compression. For this reason, we focus on tensile rather than compressive stresses when estimating fracture strength, since tensile stress governs crack opening and failure. Furthermore, FEM tends to overestimate compressive stresses at the sharp bottom corner due to stress singularities in the triangular cross-section. To avoid such artifacts, we conservatively report the tensile surface value, ${\sigma }_{{{{\rm{I}}}},\max }$ (and the corresponding ${\varepsilon }_{{{{\rm{I}}}},\max }$), as a more reliable estimate of the intrinsic fracture limit of TiB_2_. Notably, ${\sigma }_{{{{\rm{I}}}},\max }$ is a local, FEM resolved tensile side surface stress under bending and should not be interpreted as a nominal tensile stress averaged over the full cross section of a uniaxial tensile specimen. Finally, to make the stressed volume issue explicit, we postprocessed the specimen specific FEM fields to obtain the stress volume distribution for this beam (B-9), showing that the TiB_2_ volume experiencing very high tensile stress (e.g., >15 GPa) is already micrometer scale (Supplementary Note 2 and Supplementary Fig. 5). This highlights the importance of increasing the specimen cross section to ensure that a substantial material volume is subjected to high tensile stress.

**Fig. 3:**
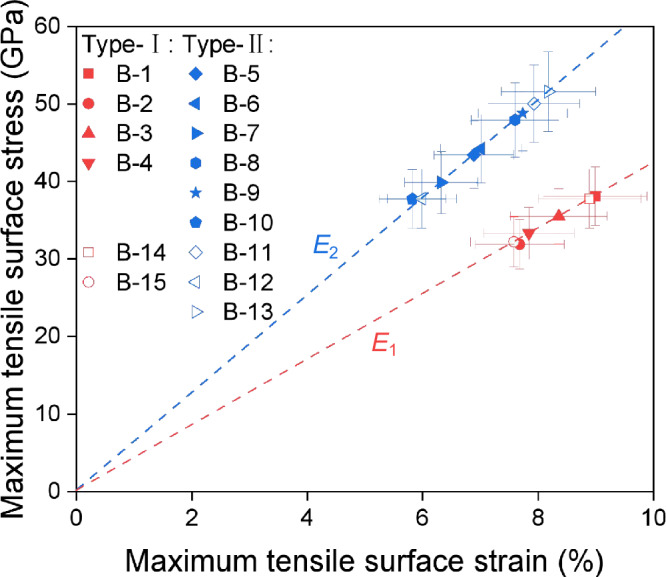
Plot of maximum tensile surface stress at failure versus corresponding maximum tensile surface strain. for Type I and Type II microcantilever and four-point bending beams, revealing only moderate anisotropy. Each symbol corresponds to one test. ${\sigma }_{{{{\rm{I}}}},\max }$ and ${\varepsilon }_{{{{\rm{I}}}},\max }$ are the maximum tensile surface stress and strain at the failure point, extracted from specimen specific FEM calibrated by the measured load-displacement curve. *E*_1_ and *E*_2_ are the Young’s moduli along the C-axis and base plane, respectively, obtained by fitting the finite element simulations. Error bars represent the simulation-derived uncertainty (±8%), rather than experimental measurement error. This uncertainty is propagated from three influencing factors: (i) matrix-support compliance, (ii) indenter-specimen friction, and (iii) SEM dimensional measurement uncertainty (Supplementary Note 3). Source data are provided as a Supplementary Source Data file.

The FEM simulations across all specimens (Supplementary Fig. 6) provide statistical insight into the anisotropy of tensile fracture strength (reflected by ${\sigma }_{{{{\rm{I}}}},\max }$). Figure 3 plots the ${\sigma }_{{{{\rm{I}}}},\max }$ of each microcantilever beam against the corresponding maximum elastic tensile strain ${\varepsilon }_{{{{\rm{I}}}},\max }$, where the slope from the origin to each point reflects the orientation-dependent elastic modulus. The error bars indicate the estimated uncertainty for each individual specimen in the FEM extracted ${\sigma }_{{{{\rm{I}}}},\max }$ and ${\varepsilon }_{{{{\rm{I}}}},\max }$ (see Supplementary Note 3 for details). Type I beams (tension along the crystallographic c-axis) exhibit ${\sigma }_{{{{\rm{I}}}},\max }$ at failure of 30–40 GPa, while Type II beams (tension perpendicular to the c-axis) reach 38–50 GPa, directly reflecting the intrinsic anisotropy of TiB_2_’s hexagonal crystal structure. Notably, high strength is retained even at the micrometer scale, as demonstrated by specimen B-7 (cross-sectional area: 7 μm^2^) achieving ~40 GPa without size-induced weakening.

**Fig. 4:**
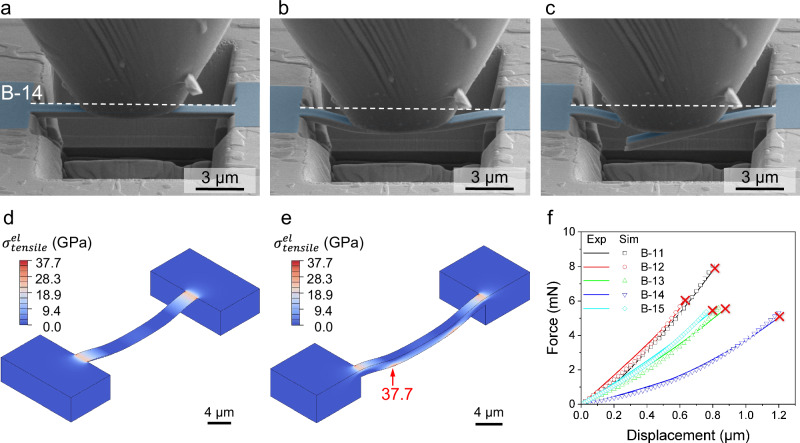
Micromechanical testing of fixed-end four-point bending beams. **a** Initial configuration, **b** elastic bending immediately prior to failure, and **c** post-mortem fracture of a representative beam (B-14). The basal planes are marked in blue. **d**, **e** FEM simulation of the bending process, reproducing the overall deformation profile and showing the distribution of tensile (principal) stress. Multiple spatially separated tensile stress “hot spots” arise within a single specimen, providing several competing sites where a critical defect—if present—could initiate failure. **e** The FEM predicted location of ${\sigma }_{{{{\rm{I}}}},\max }$, which coincides with the experimentally observed fracture origin in (**c**). **f** Experimental force-displacement curves (solid lines) compared with FEM predictions (symbols) for Type I (**e**) and Type II (**f**) beams, plotted up to the failure point (red cross symbol). Source data are provided as a Supplementary Source Data file.

The observation that micrometer-scale TiB_2_ beams reproducibly reach ultrahigh ${\sigma }_{{{{\rm{I}}}},\max }$ implies that strength-limiting defects are exceedingly scarce within the TiB_2_ particles. If failure were dominated by a conventional weakest link population of randomly distributed critical flaws (pores/microcracks), one would expect crack initiation to occur at other locations different from the ones with stress maxima, together with markedly larger specimen-to-specimen scatter even for similar orientations and sizes. To test this inference more stringently, we designed a microscale four-point bending configuration (Fig. 4a, Supplementary Fig. 7) that generates multiple spatially separated tensile stress hot spots within a single specimen, thereby providing several competing sites where a critical defect, if present, could trigger failure. Specifically, we fabricated five clamped-clamped TiB_2_ beams (Supplementary Figs. 8, 9) with dimensions summarized in Supplementary Table 4 and loaded them using a flat punch to approximate the constant moment region of four-point bending (Fig. 4b). Finite element analysis (Supplementary Fig. 10) confirms that the stress field produced by the flat punch approach closely matches that of an idealized two-line contact configuration, which is rarely available in micromechanics experiments. Further specimen specific simulations (Fig. 4d, Supplementary Figs. 8, 9), whose accuracy is validated by the excellent agreement between experimental and simulated load-displacement curves (Fig. 4e), show that the resulting boundary condition produces up to three competing tensile stress maxima (two near the fixed ends and one within the nominal constant moment region), so that defect controlled fracture could, in principle, initiate at any of these locations. Experimentally, however, all five beams fractured exactly at the FEM predicted highest stress location for that specimen (Fig. 4c, Supplementary Figs. 8, 9). In four beams, ${\sigma }_{{{{\rm{I}}}},\max }$ occurred at the upper surface edges near the fixed ends due to end constraints, and fracture initiated precisely at those edges; the remaining beam exhibited the expected constant moment stress state with the maximum tensile surface stress located on the lower surface, and fracture initiated accordingly. This one-to-one correspondence between predicted ${\sigma }_{{{{\rm{I}}}},\max }$ locations and observed fracture origins across distinct hot spots indicates that fracture is stress-controlled rather than defect-controlled, consistent with an exceptionally low population of strength-limiting defects in the in situ grown TiB_2_.

To further validate the beam-bending results, we fabricated C-shaped structures as an additional independent geometry for in situ compression testing (Supplementary Fig. 11), with their geometric information shown in Supplementary Table 5. Beyond providing a distinct stress state, this configuration offers an internal consistency check for the anisotropic elastic parameters extracted from the cantilever beams and, importantly, enables cyclic loading-unloading with an unobstructed view of the highly stressed region, thereby allowing us to probe whether any subcritical flaws accumulate damage under repeated high stress exposure. In this configuration, tensile stress develops along the outer back surface, while compressive stress acts on the inner concave side. The use of a rectangular cross-section minimizes stress singularities, enabling FEM simulations to more accurately capture the local stress distribution. Incorporating the elastic constants obtained from the cantilever tests, the simulations closely reproduced the experimental load-displacement response up to fracture (Supplementary Fig. 12), thereby confirming the reliability of the extracted material parameters (Supplementary Table 3). The C-shaped specimen reached a local maximum tensile strain of 6.1% (corresponding to a ${\sigma }_{{{{\rm{I}}}},\max }$ of 26 GPa), slightly lower than the cantilever results (Fig. 3), likely due to FIB-induced surface damage. On the compressive side, the maximum local strain was ~9.2%, corresponding to a compressive stress of 39.3 GPa (Supplementary Fig. 12).

**Fig. 5:**
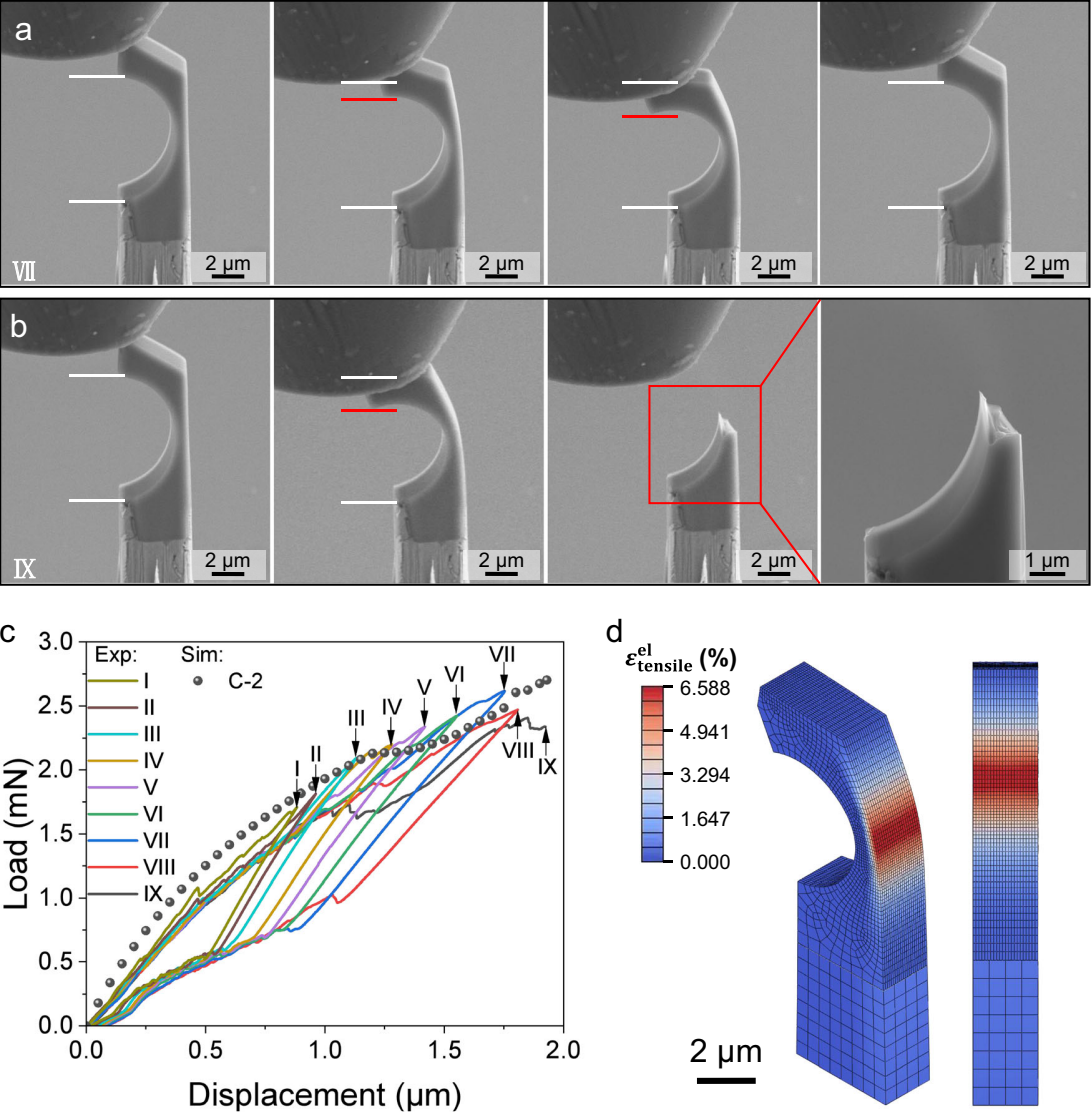
Reversible elastic behavior of a C-shaped structure. **a** SEM micrographs of specimen C-2 during loading cycle VI, showing pronounced bending and full recovery upon unloading. **b** Subsequent loading of C-2 to a higher deformation level, resulting in catastrophic fracture. Right: magnified fracture surface. **c** Load–displacement curves from multi-cycle loading–unloading tests of C-2 using a flat punch indenter tip. Source data are provided as a Supplementary Source Data file. **d** Finite element simulation of the bending process, reproducing the experimental deformation profile and mapping the local tensile maximum principal strain distribution, with a friction coefficient of *f* = 0.1.

We performed cyclic loading on an additional C-shaped TiB_2_ specimen to directly test whether repeated loading promotes microcrack nucleation or growth from any subcritical flaws. Loading-unloading tests revealed flawless recovery: the structure returned instantaneously and completely to its undeformed shape after unloading, with no measurable residual strain (Fig. 5a). Seven successive cycles with incrementally increasing peak loads (I–VII in Fig. 5c) produced perfectly overlapping force-displacement curves, confirming fully reversible, purely elastic behavior. At a maximum indenter displacement of 1.93 μm, immediately prior to fracture (Fig. 5b), the outer surface sustained a tensile strain of ~6.6% (Fig. 5d). This remarkable elasticity, maintained without sign of microcrack propagation or other dissipative mechanisms, demonstrates the absence of premature failure sources. We attribute this defect scarcity to the eutectic solidification process, highlighting its effectiveness in producing virtually flaw-free microscale ceramics capable of approaching their intrinsic elastic limits. We also emphasize that this does not imply a perfect crystal; rather, it indicates that strength-limiting defects are essentially absent at the micrometer scale.

**Fig. 6:**
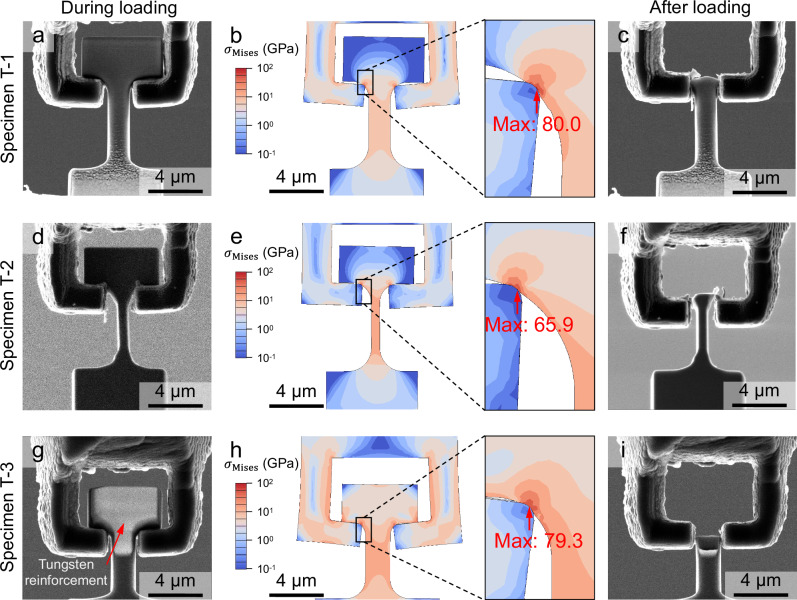
Microscale uniaxial tensile tests on TiB_2_ tensile specimens and corresponding FEM analyses. **a**–**c** Specimen T-1, **d**–**f** specimen T-2, and **g**–**i** specimen T-3. Each row corresponds to one specimen. **a**, **d**, **g** In situ SEM images at the final stable loading increment immediately prior to fracture at the grip edge. **b**, **e**, **h** Corresponding specimen-specific FEM maps of von Mises stress at the same applied displacement. **c**, **f**, **i** Post-mortem SEM images after failure. In all three tests, FEM reveals a strong stress concentration at the clamp edge, consistent with the experimentally observed fracture location, while the gauge section remains crack-free up to the onset of clamp-edge failure. These tensile tests therefore provide a lower-bound tensile benchmark for the gauge section, rather than a direct measurement of intrinsic tensile fracture strength.

Finally, we attempted direct micrometer-scale tensile testing to probe a larger, nearly uniformly stressed gauge volume under nominal uniaxial loading. Three micrometer-sized TiB_2_ tensile specimens (T-1 to T-3) were FIB-fabricated and pulled using PFIB machined diamond microgrippers (Supplementary Fig. 13). In all three tests, fracture occurred reproducibly at the grip edge rather than within the calibrated gauge section (Fig. 6, Supplementary Fig. 14), indicating that failure was governed by unavoidable stress concentration introduced by the gripping condition. This conclusion is supported by both the SEM fracture morphologies and the similar maximum loads sustained by the specimens despite their different cross-sectional areas (Supplementary Fig. 14). FEM analysis of the specimen gripper assembly further reveals a severe local multiaxial stress concentration at the grip edge, while the gauge region sustains a nearly uniform tensile stress of ~13 GPa immediately prior to fracture (Fig. 6, Supplementary Fig. 15). Because the maximum load is similar across specimens, the uniform gauge stress decreases with increasing cross-sectional area. This dominant stress concentration could not be eliminated by either narrowing the gauge width (T-2) or reinforcing the gripped region via deposition of a protective tungsten layer over the grip section (T-3). Therefore, these microscale tensile tests do not provide a direct measurement of the intrinsic tensile fracture strength of TiB_2_. Rather, they establish a conservative lower-bound tensile benchmark: the gauge section, with a volume exceeding 4 μm^3^, remains intact up to ~13 GPa of near uniform tensile stress prior to grip-controlled failure. This result shows that the present truly micrometer-scale TiB_2_ volume can sustain very high tensile stress without detectable cracking, supporting an exceptionally low population of strength-limiting defects in our TiB_2_. To directly examine whether large TiB_2_ particles contain hidden internal flaws, we serially sectioned three large TiB_2_ particles by FIB and imaged each newly exposed cross-section by SEM. Across multiple slices along the [0001] direction, the TiB_2_ interior remains fully dense and exhibits uniform contrast, with no pores, microcracks, or discontinuities that persist from slice to slice (Supplementary Fig. 16). This provides direct microstructural evidence that the measured ultrahigh strengths are governed by the applied local tensile stress maximum rather than preexisting internal flaw populations. As a further complementary check, we performed in situ compression tests on three FIB-fabricated single crystal TiB_2_ micropillars (Supplementary Figs. 17, 18), obtaining nominal peak compressive stresses of 31, 43, and 33 GPa for specimen volumes of 1.1, 1.8, and 9.7 μm^3^, respectively. Although compressive loading suppresses crack opening and is intrinsically less sensitive to strength-limiting flaws than tensile loading in brittle solids, these results provide an independent validation that the present TiB_2_ sustains stresses in the tens-of-gigapascal regime at the micrometer scale, consistent with the bending-based measurements.

**Fig. 7:**
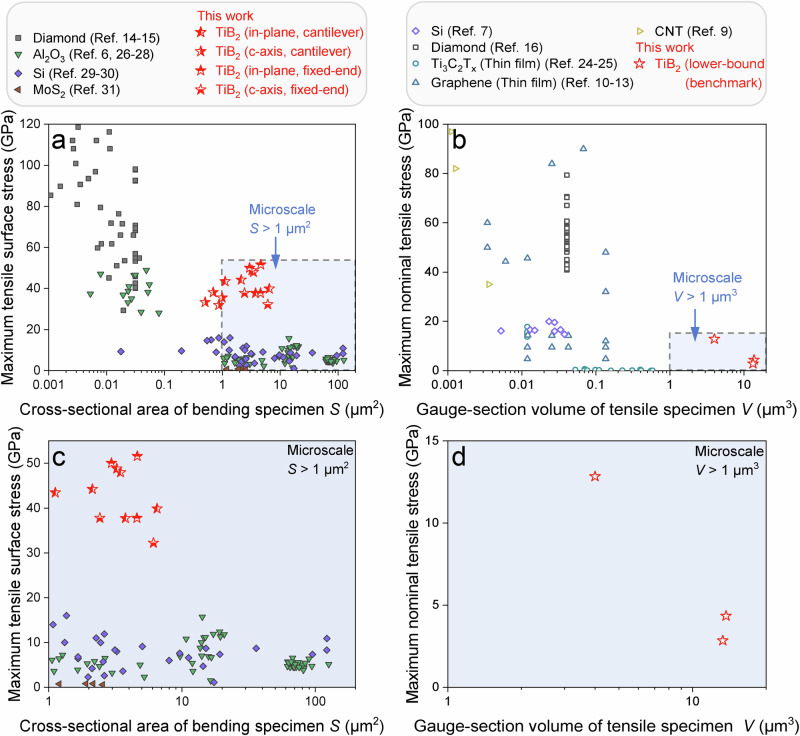
Strength limits of brittle solids as a function of specimen cross-sectional area, separated by loading mode to avoid mixing stress definitions. **a** Bending derived strength data reported as the maximum tensile surface stress at failure, ${\sigma }_{{{{\rm{I}}}},\max }$ (tensile side fracture stress), plotted against cross-sectional area (used here as a practical length scale descriptor). Our TiB_2_ results are shown for two loading geometries (cantilever and fixed end four-point bending) and two crystal directions (along the c-axis and within the basal plane). **b** Tensile-derived strength data from true uniaxial tensile tests, reported as the maximum nominal tensile stress plotted against the gauge-section volume. For our micrometer-scale TiB_2_, the plotted value corresponds to the nearly uniform tensile stress (up to ~13 GPa) sustained within a large gauge volume (>4 μm^3^) immediately prior to grip edge failure and therefore represents a conservative lower bound. **c** Enlarged view of panel (a) for the micrometer scale regime with the x-axis starting at 1 μm^2^. **d** Enlarged view of (**b**) for the micrometer scale regime with the x-axis starting at 1 μm^3^. Literature datasets include diamond^14–16^, graphene^10–13^, carbon nanotubes (CNT)^9^, Ti_3_C_2_T_x_^24,25^, Al_2_O_3_^6,26–28^, Si^7,29,30^ and MoS_2_^31^. Literature sources and the associated loading mode are listed in Supplementary Table 10.

We summarize our TiB_2_ results and compare them with literature data for brittle solids using consistent loading modes and stress/strain definitions. Accordingly, we separate bending-derived and tensile-derived datasets into two panels (Fig. 7a, b), with the data sources and their testing details summarized in Supplementary Table 10, thereby avoiding ambiguity that arises when “cross-sectional area” is used across fundamentally different stress states. Figure 7 focuses on stress values for these mode separated datasets, whereas the corresponding strain values for the same sources is provided in Supplementary Fig. 19. Figure 7a includes only bending type measurements, reported as the maximum tensile surface stress at failure, ${\sigma }_{{{{\rm{I}}}},\max }$ (i.e., the tensile side fracture stress), as defined or extracted in the original references, together with our microcantilever and four-point bending results reported using the same definition. In this bending only panel, the x-axis “cross-sectional area” is retained solely as a practical length scale descriptor: for brittle fracture under bending, a larger cross-section generally corresponds to a larger high-tensile-stress region and thus a higher probability of sampling a critical flaw (a weakest link size effect). Consistent with this expectation, the literature data reveal a clear size effect: ${\sigma }_{{{{\rm{I}}}},\max }$ decreases as cross-sectional area increases, particularly once it exceeds ~1 µm^2^. Despite this trend, our in situ grown single crystal TiB_2_ sustains tensile strains up to 9.0% (Supplementary Fig. 19)—corresponding to ${\sigma }_{{{{\rm{I}}}},\max }\approx$E/11—even at micrometer scale cross sections, thereby approaching the theoretical elastic limit. To date, such large elastic strains at this scale have only been reported for defect minimized silicon fabricated by advanced semiconductor processing tested by bending^8^, yet TiB_2_ markedly outperforms silicon in ${\sigma }_{{{{\rm{I}}}},\max }$ by multiple factors due to its far higher modulus (Fig. 7c).

Figure 7b compiles only tensile type measurements from true uniaxial tensile tests, together with our micrometer scale tensile benchmark for TiB_2_. Notably, for brittle materials, successful reports of micrometer scale tensile fracture stress with a truly micrometer scale gauge volume remain essentially absent (Fig. 7d), largely because unavoidable grip induced stress concentrations tend to dominate failure. Consistent with this limitation, our TiB_2_ tensile specimens fail prematurely at the grip. Nevertheless, the gauge section with a large volume (>4 μm^3^) sustains a nearly uniform, very high tensile stress up to ~13 GPa prior to grip edge failure. This value therefore provides a conservative lower bound, rather than the tensile fracture strength of the gauge section itself, and demonstrates that micrometer-scale TiB_2_ volumes can sustain exceptionally high, near-uniform tensile stress without fracture, partially filling a key gap in the literature regarding the attainable tensile stress level for brittle materials at this length scale. Overall, these mode-separated comparisons reinforce a central message: when TiB_2_ is formed in situ by eutectic solidification, micrometer-scale specimens can approach near-theoretical elastic limits because strength-limiting defects are exceedingly scarce.

In summary, the TiB_2_ steel system provides a compelling case study showing that cost-effective, in situ grown microscale ceramics can match nanoscale materials in approaching near-theoretical strength and elasticity. These near-flawless phases, capable of sustaining large, fully reversible strains, deliver exceptional mechanical reliability while avoiding the fabrication complexity and size constraints inherent to nanostructures. Because the approach relies on generic eutectic solidification, it should extend to other systems, offering a scalable metallurgical route to ultrahigh performance materials and microarchitectures—from actuators^17^ to microrobots^19^—without exclusive reliance on conventional nanomaterial synthesis methods.

## Methods

### Fabrication of micromechanical TiB_2_ specimens

Two TiB_2_ reinforced high modulus steels (HMS-1 and HMS-2; compositions in Supplementary Table 9) were prepared to provide micrometer-sized TiB₂ while isolating any matrix composition effects on the mechanical properties of the in situ TiB_2_. Ingots were produced by vacuum melting and hot rolling. TiB_2_ is generated during casting by eutectic/near-eutectic solidification in the Fe-Ti-B system. During cooling, Ti and B exhibit limited solubility in Fe and partition to form Ti-B-enriched liquid regions; TiB_2_ precipitates from these regions as primary faceted crystals. As solidification proceeds, the remaining Fe-rich liquid transforms into the steel matrix while preserving the already formed TiB_2_ (Supplementary Note 1), yielding the partially embedded hexagonal prismatic TiB_2_ morphology observed after deep etching (Supplementary Fig. 1). Sample surfaces were prepared by sequential grinding (80-3000 grit), polishing with diamond suspensions, and final vibratory polishing with a 50 nm SiO_2_ colloidal suspension. TiB_2_ was then exposed by deep etching in 33 vol% HCl.

To effectively vary the stress state and specimen volume for robust failure analysis, we employed five complementary specimen geometries, including (i) microcantilever beams (B-1 to B-10), (ii) clamped-clamped (fixed end) microscale four-point bending beams (B-11 to B-15), (iii) C-shaped structures (C-1 and C-2), (iv) microscale tensile specimens (T-1 to T-3), and (v) microscale compression pillars (P-1 to P-3). Focused ion beam (FIB) milling was performed using a Ga+ ion source at 30 kV in a Helios 5UC SEM/FIB system (Thermo Fisher Scientific, US). The SEM observation was performed in secondary-electron mode using the Everhart Thornley detector at an accelerating voltage of 10 kV, a beam current of 0.56 nA, and a working distance of 4 mm. A high ion beam current of 2–21 nA was first used for coarse trench milling around the target TiB_2_ particle to remove the surrounding steel matrix and isolate the region of interest. During coarse milling, intermediate FIB milling at 300–700 pA was used for specimen shaping and for all auxiliary preparation steps, including Pt or W deposition assisted fixation/protection. Final low-current milling at 100 pA was then applied to all specimens as the last cleaning step to smooth the surfaces, remove redeposited material, and minimize ion-induced damage. For the compression tests, three additional single-crystal TiB_2_ micropillars were fabricated from exposed TiB_2_ particles, including one cuboid pillar (P-1) and two cylindrical pillars (P-2 and P-3). Processing flow diagram, schematics and geometric parameters for the cantilevers, four-point bending beams, C-shaped structures, tensile specimens, and compression pillars are provided in Supplementary Figs. 2, 7, 11, 13, and 17, with dimensions summarized in Supplementary Tables 1-2, 4–8. For the four-point bending beams and cantilevers, the top tensile surface was intentionally preserved from direct FIB exposure (Supplementary Figs. 2 and 7) to minimize ion-induced surface flaws in the most highly stressed region.

### In situ micromechanical testing

Mechanical testing was performed on a micro/nanomechanical platform (FT-NMT04, FemtoTools, Switzerland) equipped with flat punch and cube corner indenter tips. Unless otherwise stated, tests were conducted under displacement control at 5 nm/s, with load and displacement recorded at a sampling rate of 3 kHz. Real-time imaging was used to track indenter position and specimen response, enabling correlation of mechanical data with the onset and location of cracking/fracture.

For bending tests (cantilevers and fixed-end four-point bending beams), loading was applied normal to the specimen surface. Beams B-1 to B-6 were loaded with a flat punch to maintain full face contact. For microcantilever beams B-7 to B-10 machined on smooth surfaces, a cube corner indenter was used to precisely define the loading distance (Supplementary Fig. 2d2), eliminating the edge-position ambiguity of a flat punch. For fixed-end beams B-11 to B-15, flat punch loading was used to approximate a constant moment region in the four-point bending geometry. For C-shaped structures, flat punch loading enabled monotonic loading to fracture (Supplementary Fig. 12) and cyclic loading-unloading with unobstructed views to probe damage accumulation under repeated high stress exposure. For microscale tensile tests, TiB_2_ tensile specimens were pulled using a PFIB-machined diamond gripper design (Supplementary Fig. 13). SEM images of the tensile specimens and representative fracture morphologies are provided in Supplementary Fig. 14. For microscale compression tests, TiB_2_ micropillars P-1 to P-3 were compressed using a diamond flat punch indenter under displacement control. The nominal engineering compressive stress was calculated from the applied load divided by the initial pillar cross-sectional area, and the nominal engineering compressive strain was calculated from the indenter displacement divided by the initial pillar height. Representative prefracture and postfracture SEM images, together with the corresponding nominal stress-strain curves, are provided in Supplementary Fig. 18.

### FEM analyses

Finite element (FE) simulations were performed in Abaqus/CAE (Version 2023, Dassault Systems) for each tested specimen geometry (cantilever, fixed end four-point bending, C-shaped, and tension) using three-dimensional models discretized with 10-node quadratic tetrahedra elements (C3D10). Each model contained ~10^6^ elements with mesh refinement in high-gradient regions (e.g., contact and fillet zones). Model dimensions were extracted from SEM measurements of each specimen. Single-crystal TiB_2_ was modeled as a transversely isotropic, linearly elastic solid. The anisotropic elastic constants were determined by fitting simulated and experimental load–displacement responses of the cantilever beams and then applied without further adjustment to the other geometries (four-point bending and C-shaped), providing an internal consistency check. During FIB fabrication, the TiB_2_ particles retained sufficient supporting volume; consequently, elastic deformation of the surrounding matrix had a negligible influence on the beam’s load-bearing stress-strain response (Supplementary Fig. 20). Indenter tips were modeled as rigid bodies, and surface-to-surface contact was used between the indenter and TiB_2_. To capture potential friction effects in bending, surface-to-surface contact between the indenter tip and specimen was defined with Coulomb friction (*f* varied from 0.1 to 1). The extracted ${\sigma }_{{{{\rm{I}}}},\max }$ and ${\varepsilon }_{{{{\rm{I}}}},\max }$ were insensitive to *f* over this range (Supplementary Fig. 21).

To estimate the uncertainty shown as error bars in Fig. 3, we quantified the sensitivity of the FEM extracted ${\sigma }_{{{{\rm{I}}}},\max }$ and ${\varepsilon }_{{{{\rm{I}}}},\max }$ to three dominant inputs: (i) compliance of the supporting matrix beneath the TiB_2_ beam (rigid versus elastoplastic substrate), (ii) the indenter specimen Coulomb friction coefficient (*f* = 0.1–1.0), and (iii) SEM based dimensional measurement uncertainty of beam width and height (±30 nm edge-definition uncertainty). The resulting variations were ±1%, ±4%, and ±3%, respectively (Supplementary Figs. 20–22), which we conservatively summed to obtain an overall ±8% uncertainty per specimen (Fig. 3).

For bending-based tests (cantilevers and fixed-end four-point bending), the reported “fracture strength” corresponds to the maximum tensile (principal) surface stress at the failure point, defined as the peak value of the maximum principal tensile stress on the tensile surface at the final stable loading increment immediately prior to the first cracking event. This is a local, FE-resolved surface stress in bending and should not be interpreted as a nominal uniaxial tensile stress averaged over the full cross-section. The corresponding maximum tensile strain was extracted from the same location and loading increment. For microscale tensile specimens, FE simulations incorporating the measured grip alignment were used to quantify the stress distribution and to identify the nearly uniform tensile stress sustained in the gauge section immediately prior to grip edge failure (Supplementary Fig. 14). Accordingly, the tensile value reported (~13 GPa) is treated only as a conservative lower bound tensile benchmark for the unbroken gauge section, rather than as the intrinsic tensile fracture strength of TiB_2_. Any local von Mises stress at the grip edge is used solely to illustrate the severity of the grip induced multiaxial stress concentration and is not compared directly with the bending-derived tensile fracture stresses.

To explicitly quantify the stressed volume associated with high tensile stresses in bending, we postprocessed FE stress fields to compute the cumulative TiB_2_ volume exceeding a chosen tensile stress threshold by integrating element volumes as a function of maximum principal tensile stress. This stress volume analysis was performed for representative cantilever beams with the largest cross sections to directly assess the micrometer scale volume subjected to ultrahigh tensile stress.

### Cross-sectional sectioning to assess internal flaws

To evaluate whether large TiB_2_ particles contain internal pores or microcracks that could act as strength-limiting defects, three representative large single-crystal TiB_2_ particles were randomly selected and serially sectioned by FIB along the [0001] direction and imaged by SEM (Supplementary Fig. 16).

## Supplementary information

Supplementary Information

Description of Additional Supplementary Files

Supplementary Movie 1

Supplementary Movie 2

Supplementary Movie 3

Supplementary Movie 4

Supplementary Movie 5

Supplementary Movie 6

Supplementary Movie 7

Supplementary Movie 8

Transparent Peer Review file

## Source data

Source Data

## Data Availability

The data that supports the findings of this study are provided in the Supplementary Information and Source Data file. Source data are provided with this paper.
