# Supplementary material for: Achieving near-theoretical strength and high elasticity in micrometer scale TiB2 ceramics
Source: Nat Commun. 2026 Jul 1;17:5672. doi: 10.1038/s41467-026-74750-1 (PMC13323769; doi:10.1038/s41467-026-74750-1)
Supplement: Supplementary file 1 — Supplementary Information [file 41467_2026_74750_MOESM1_ESM.pdf]

Supplementary Information for

Achieving near-theoretical strength and high elasticity in micrometer scale TiB_2 ceramics

Qianduo Zhuang [a], Yizhuang Li [a, *], Fanghai Xin [b], Mingxin Huang [c], Wei Xu [a, *]

[a] State Key Laboratory of Digital Steel, Northeastern University, Shenyang, China

[b] K.H Kuo Center for Material Characterization, Liaoning Academy of Materials, Shenyang, China

[c] Department of Mechanical Engineering, The University of Hong Kong, Hong Kong, China

*Corresponding author: Yizhuang Li, Email: liyz@mail.neu.edu.cn

Wei Xu, Email: xuwei@mail.neu.edu.cn

Table of Contents

Supplementary Notes 1-3 p. 2

Supplementary Figures 1-22 p. 5

Supplementary Tables 1-10 p. 29

Supplementary References p. 40

Supplementary Note 1. In situ formation of micrometer scale TiB_2 by Fe-Ti-B eutectic/near-eutectic solidification.

In this work, micrometer scale TiB_2 forms in situ during casting through eutectic/near-eutectic (often hypereutectic) solidification in the Fe-Ti-B system using Ti/B-enriched high modulus steels (HMS-1 and HMS-2; compositions in Supplementary Table 9). The resulting faceted hexagonal prismatic TiB_2 morphology is shown in Supplementary Fig. 1.

The formation pathway can be summarized as follows. (i) Solute enrichment prior to solidification: after Ti and B are introduced into the melt, their limited solubility in Fe and strong chemical affinity drive local Ti-B enrichment in the liquid, creating Ti-B-rich liquid pockets as cooling proceeds. (ii) Primary TiB_2 formation from a hypereutectic liquid: upon the onset of solidification, primary single crystal TiB_2 precipitates directly from these Ti-B-rich regions (hypereutectic solidification), and continued TiB_2 growth progressively depletes Ti and B from the surrounding melt, rendering the remaining liquid increasingly Fe-rich. This can be expressed schematically as $L_{(\mathrm{Ti,\ B\ rich})} \rightarrow TiB_2 + L_{(\mathrm{Fe\ rich})}$. The characteristic hexagonal prismatic morphology arises from anisotropic growth kinetics dictated by the TiB_2 crystal structure: growth along close-packed planes is slow because fewer atomic attachment sites are available, whereas less close-packed (inclined) planes advance faster and are eventually eliminated, leaving the slow growing basal (0001) and prismatic {10-10} facets as the dominant particle surfaces, consistent with Supplementary Fig. 1. (iii) Secondary TiB_2 during final solidification: as the residual Fe-rich liquid solidifies to the steel matrix, Ti and B are rejected to the solidification front and additional fine TiB_2 forms by the terminal eutectic reaction, $L_{(\mathrm{Fe\ rich})} \rightarrow TiB_2 + Fe$, completing the in situ TiB_2 dispersion.

Supplementary Note 2. Quantifying stressed volume in cantilever bending via stress volume distributions.

A common concern in microcantilever bending is that the maximum tensile stress occurs only within a limited region near the fixed end. To quantify this "stressed volume" issue explicitly (rather than qualitatively), we compute stress volume distributions from the specimen specific FEM stress fields for the representative beam B-9 (shown in Fig. 2).

We use the maximum principal stress and restrict the analysis to tensile values. This choice is consistent with brittle fracture being controlled by crack opening under tension and with the stress definition reported throughout the manuscript. Two different but related quantities were then extracted from the FEM results. For each FEM mesh, we post process all elements and calculate: (i) the TiB_2 volume in each stress bin, obtained by summing element volumes whose local tensile stress falls within each individual stress interval. This quantity is shown in Supplementary Fig. 5a for beam B-9; and (ii) the TiB_2 volume above a chosen stress threshold, obtained by summing all TiB_2 element volumes whose local tensile stress is higher than that threshold. This quantity is shown in Supplementary Fig. 5b for beam B-9. These two quantities answer different questions. Supplementary Fig. 5a shows how much TiB_2 volume lies within each individual stress interval, whereas Supplementary Fig. 5b shows the total TiB_2 volume subjected to stresses higher than a selected threshold.

The key outcome is that the material volume subjected to very high tensile stress is already micrometer scale: the cumulative volume above a high stress threshold of 15 GPa exceeds 5 μm^3. Therefore, although the stress field in cantilever bending is non-uniform, the present experiments do not rely on an ultrasmall stressed volume to reach ultrahigh tensile stresses prior to failure.

This micrometer scale high stress volume is consistent with an exceptionally low density of critical, strength limiting flaws in the in situ grown TiB_2, because failure at such high tensile stress sustained over μm^3-scale volumes would otherwise be unlikely under a weakest link scenario.

Supplementary Note 3. Uncertainty analysis for FEM extracted $\sigma_{\mathrm{I,max}}$ and $\varepsilon_{\mathrm{I,max}}$.

The error bars plotted in Fig. 3 represent the estimated uncertainty in the FEM extracted maximum tensile surface stress and strain at fracture for each individual beam. They do not represent specimen to specimen scatter. Because $\sigma_{\mathrm{I,max}}$ and $\varepsilon_{\mathrm{I,max}}$ are obtained by combining the experimental load-displacement curve with specimen specific FEM, the uncertainty is dominated by the sensitivity of the extracted peak tensile quantities to model inputs and geometric measurements.

We quantified three dominant contributions:

(i) Matrix support compliance beneath the beam: replacing a rigid support with an elastoplastic matrix changes the extracted $\sigma_{\mathrm{I,max}}$ and $\varepsilon_{\mathrm{I,max}}$ by $\leq \pm 1\%$ (Supplementary Fig. 20).

(ii) Indenter specimen friction: varying the Coulomb friction coefficient over a broad range f = 0.1-1.0 changes $\sigma_{\mathrm{I,max}}$ and $\varepsilon_{\mathrm{I,max}}$ by $\leq \pm 4\%$ (Supplementary Fig. 21).

(iii) Dimensional measurement uncertainty: perturbing the SEM measured beam width and height by ±30 nm (edge definition uncertainty) changes $\sigma_{\mathrm{I,max}}$ and $\varepsilon_{\mathrm{I,max}}$ by $\leq \pm 3\%$ (Supplementary Fig. 22).

Taking a conservative approach, we sum these contributions (1% + 4% + 3%) to obtain an overall uncertainty of ±8%, which is plotted as the error bars in Fig. 3.

Furthermore, in two cantilever tests (B-3 and B-5), the first detectable crack appears on the compressed lower surface near the fixed end and produces a sudden but limited load drop (Supplementary Fig. 4). To apply a uniform and conservative criterion across all beams, the FEM extracted $\sigma_{\mathrm{I,max}}$ and $\varepsilon_{\mathrm{I,max}}$ for these two specimens are evaluated at the last stable loading increment immediately before this first load drop (i.e., prior to any detectable cracking). Using this convention, the peak tensile side stresses are 35.5 GPa (B-3) and 43.4 GPa (B-5).

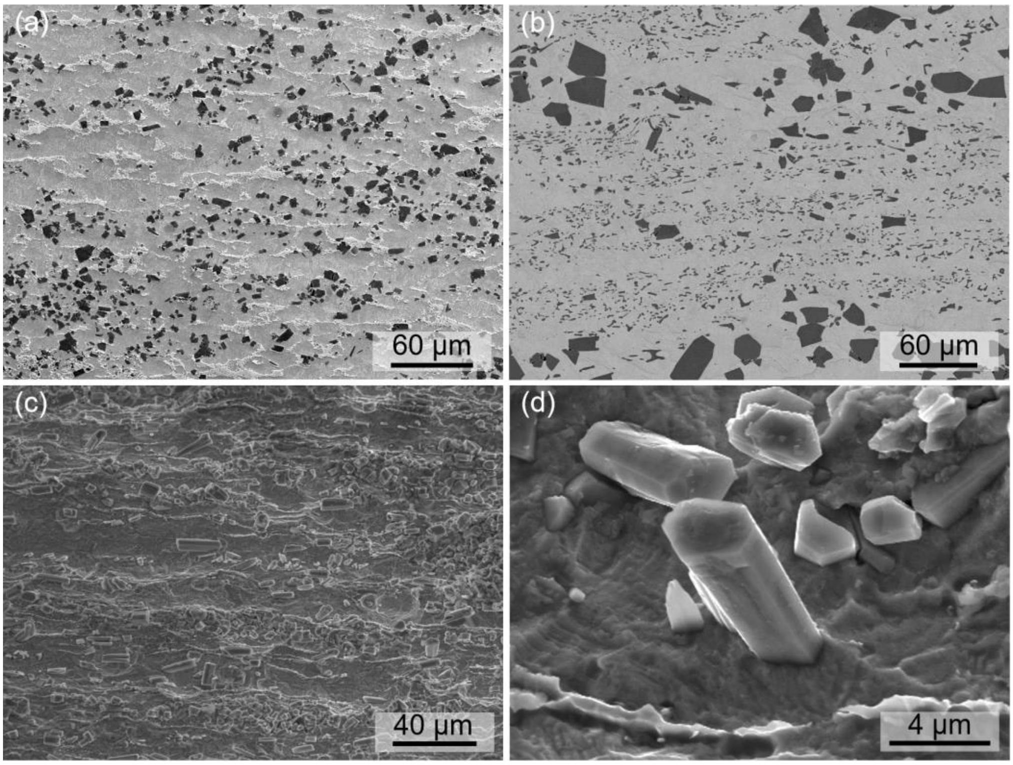

Supplementary Figure 1. Microstructure of the high modulus steels (HMSs) used in this work. (a,b) SEM micrographs of polished sample surfaces of HMS-1 and HMS-2, respectively. (c) TiB_2 particles with varied morphologies revealed by deep etching of HMS-1. (d) Representative TiB_2 particle with hexagonal prismatic morphology.

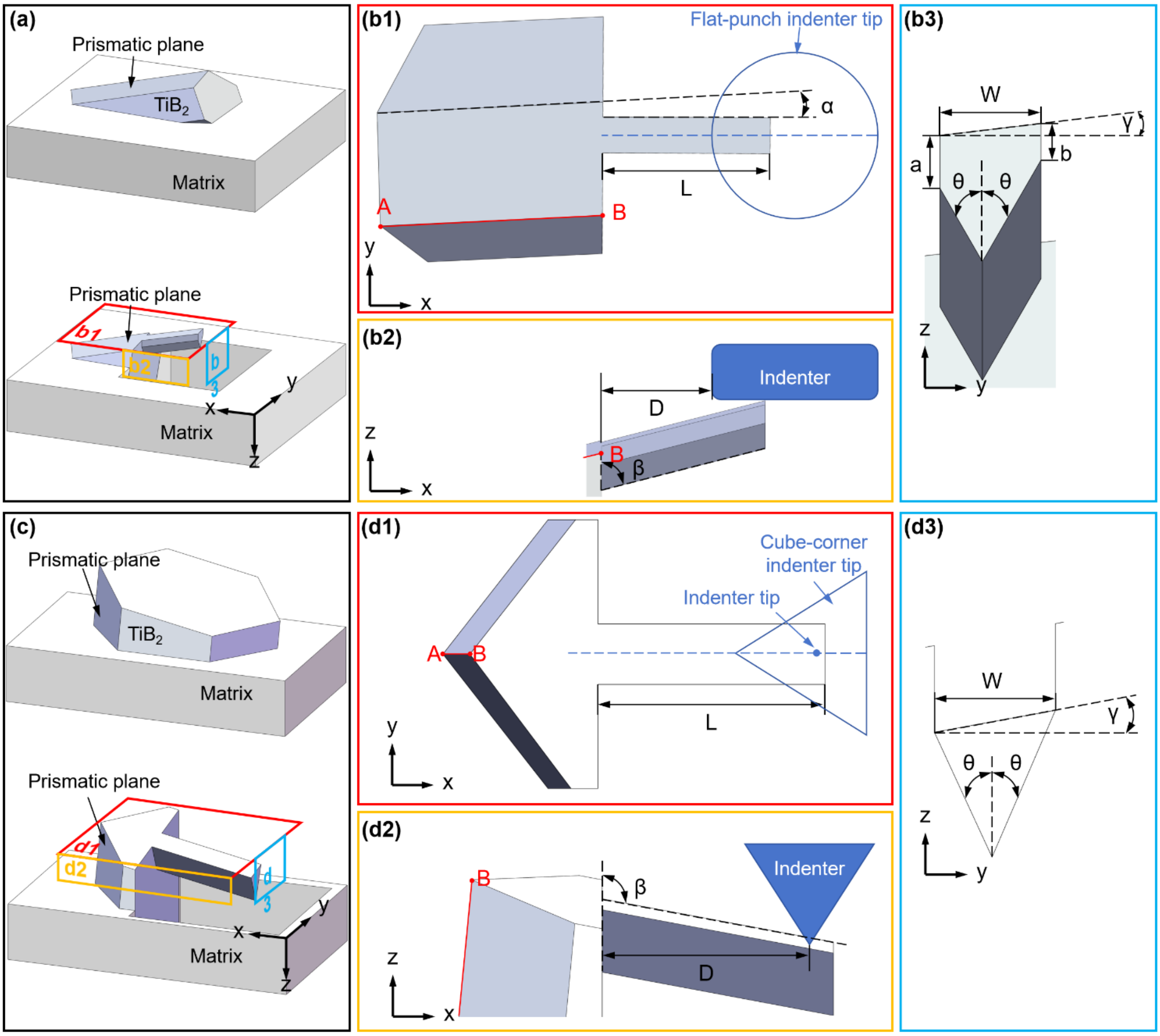

Supplementary Figure 2. Schematic of single crystal TiB_2 particles partially embedded in a steel matrix and the resulting microcantilever geometries after FIB milling: (a) type I and (c) type II. By design, the top surface of each microcantilever is not exposed to FIB and remains unaffected. (b1–b3) Top, front, and side views of the type I cantilever in (a); (d1–d3) corresponding views for the type II cantilever in (c). Panels (b) and (d) define the distance D, measured from two characteristic indenter positions to the end of the cantilever. For orientation reference in the anisotropy analysis, the side edge of the TiB_2 particle is marked "AB", which coincides with the hexagonal c-axis.

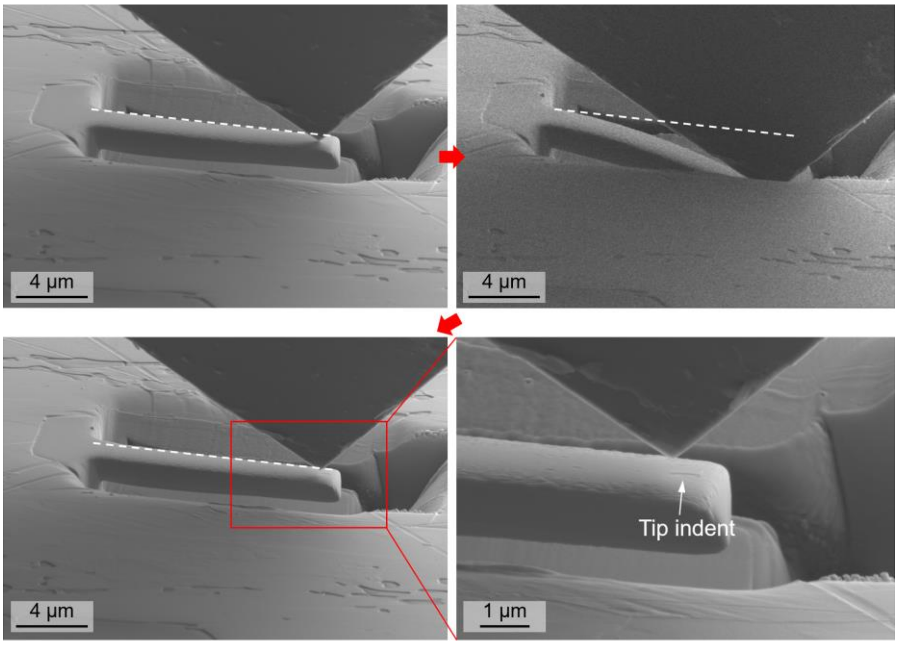

Supplementary Figure 3. SEM micrographs showing the reversible bending deformation of a microcantilever beam during loading and its full recovery upon unloading. The small tip indent is clearly visible.

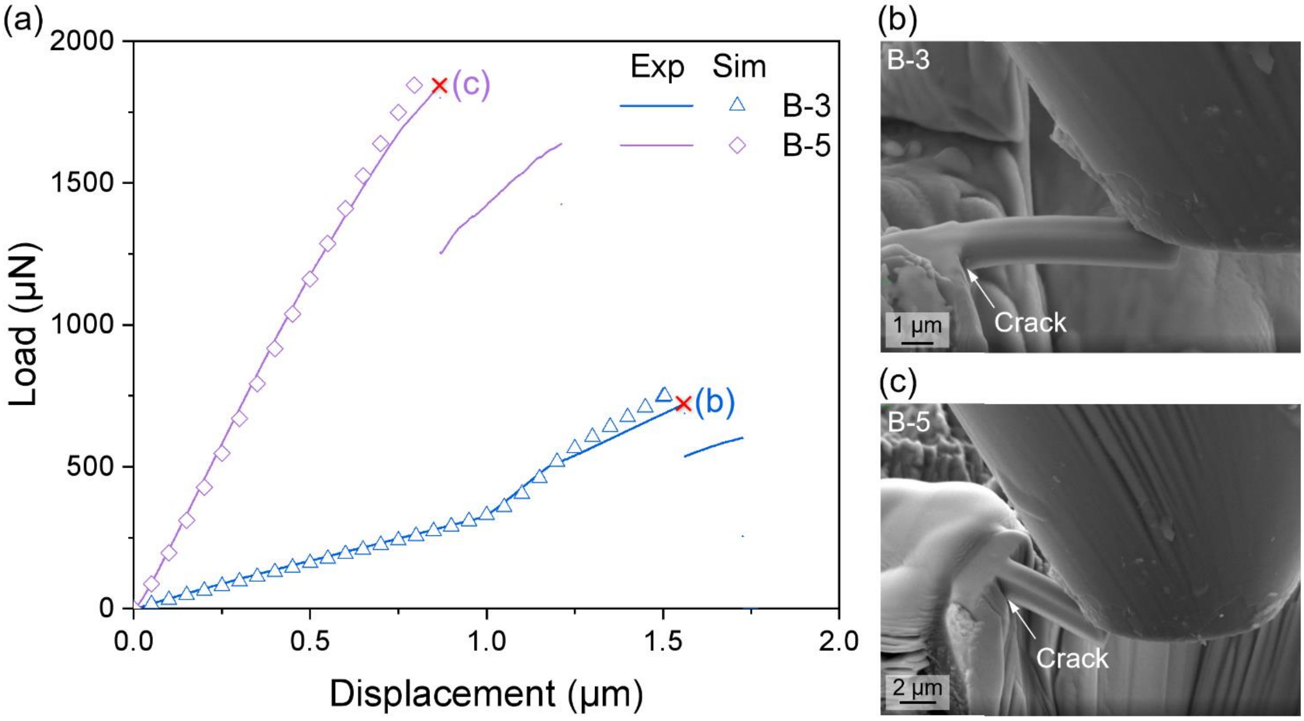

Supplementary Figure 4. Crack initiation on the compressed lower surface in B-3 and B-5 and definition of the failure point used for FEM extraction. (a) Complete experimental force displacement curves (solid lines) compared with FEM simulations (symbols) for B-3 and B-5. The cross symbol marks the displacement corresponding to the last stable increment immediately before the first load drop, which is used to extract $\sigma_{\mathrm{I,max}}$ and $\varepsilon_{\mathrm{I,max}}$. (b,c) Representative SEM video frames captured at the moment when the first detectable microcrack appears near the bottom (compressive) corner close to the fixed end in (b) B-3 and (c) B-5.

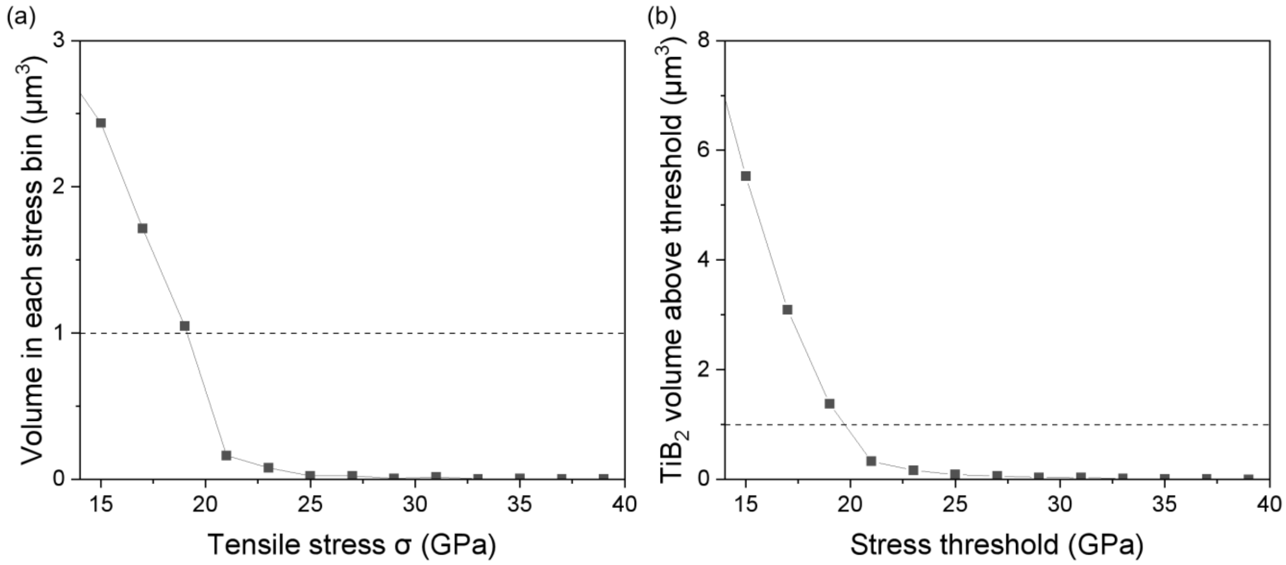

Supplementary Figure 5. Stress volume analysis for the representative cantilever beam B-9. (a) Volume in each stress bin, showing the TiB_2 volume contained within each individual tensile stress interval. (b) TiB_2 volume above threshold, showing the cumulative TiB_2 volume subjected to tensile stress higher than a selected threshold. The analysis shows that the TiB_2 volume subjected to very high tensile stress is already micrometer scale; for example, the cumulative volume above 15 GPa exceeds 5 μm^3 despite the non-uniform bending stress field.

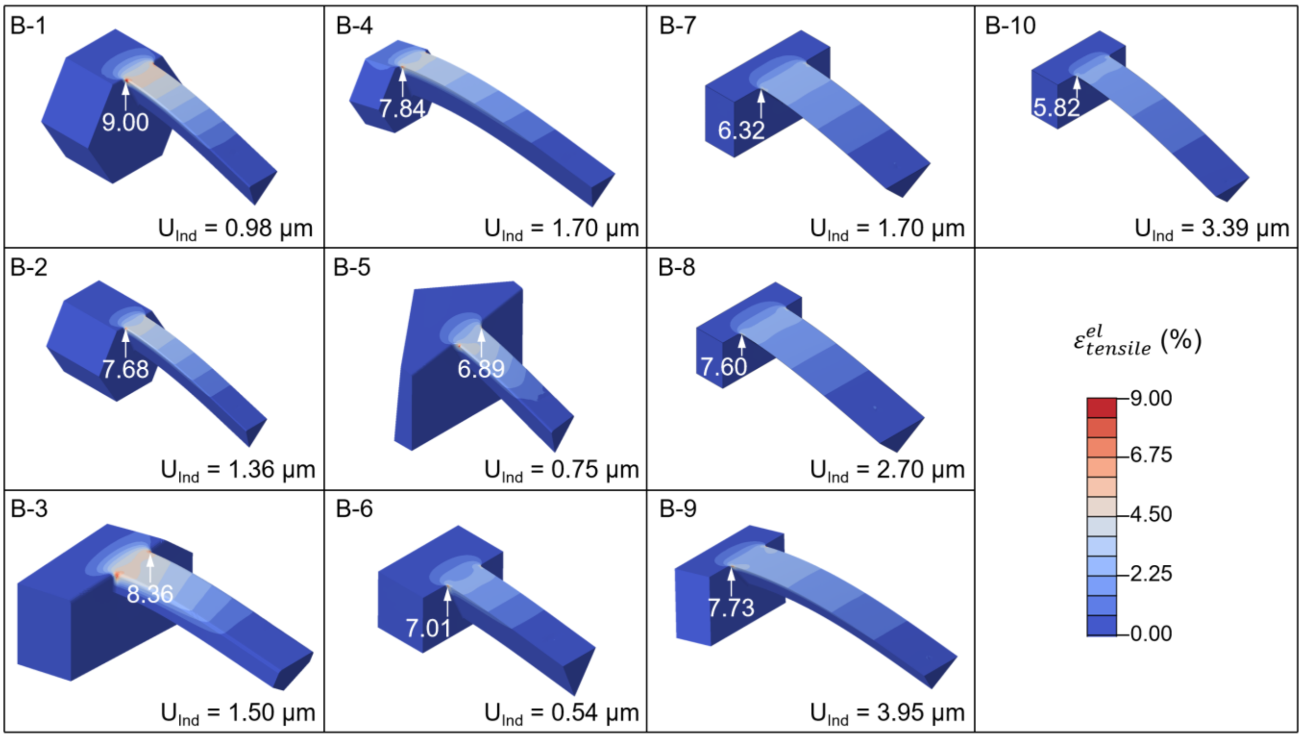

Supplementary Figure 6. Finite element simulation of the bending process showing the tensile strain distribution for specimens B-1 to B-10 prior to failure. U_{ind} is the displacement of indenter. The maximum tensile strain for each specimen is annotated.

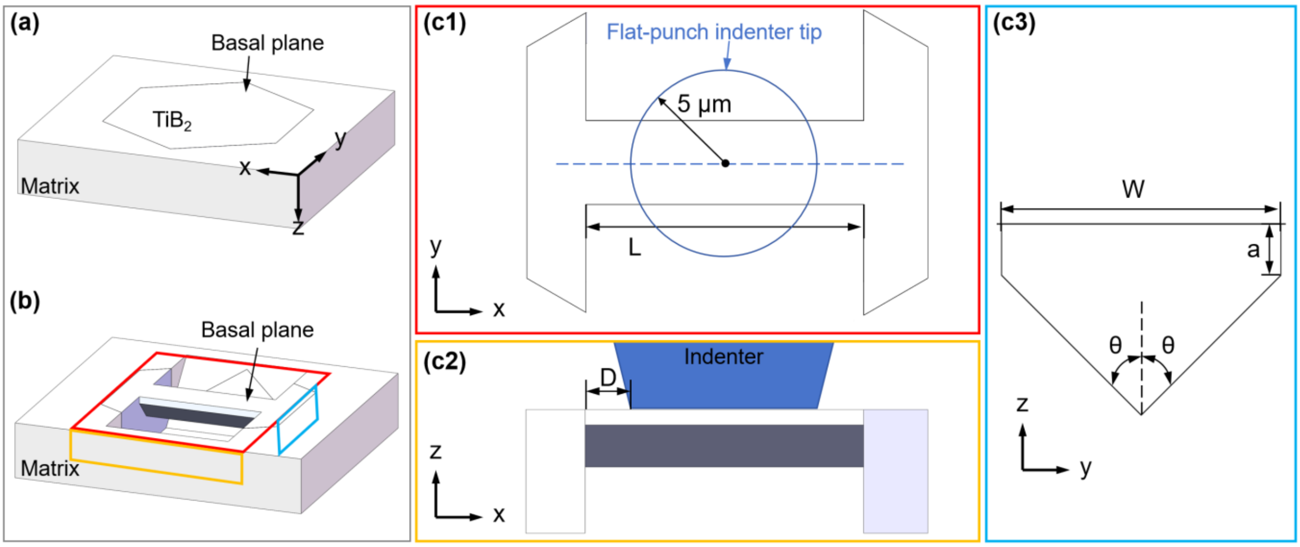

Supplementary Figure 7. Schematic of single crystal TiB_2 particles partially embedded in a steel matrix and the resulting four-point bending beams (a) before and (b) after milling. By design, the top surface of each four-point bending beam is not exposed to FIB and remains unaffected. (c1–c3) Top, front, and side views of the four-point bending beam in (b).

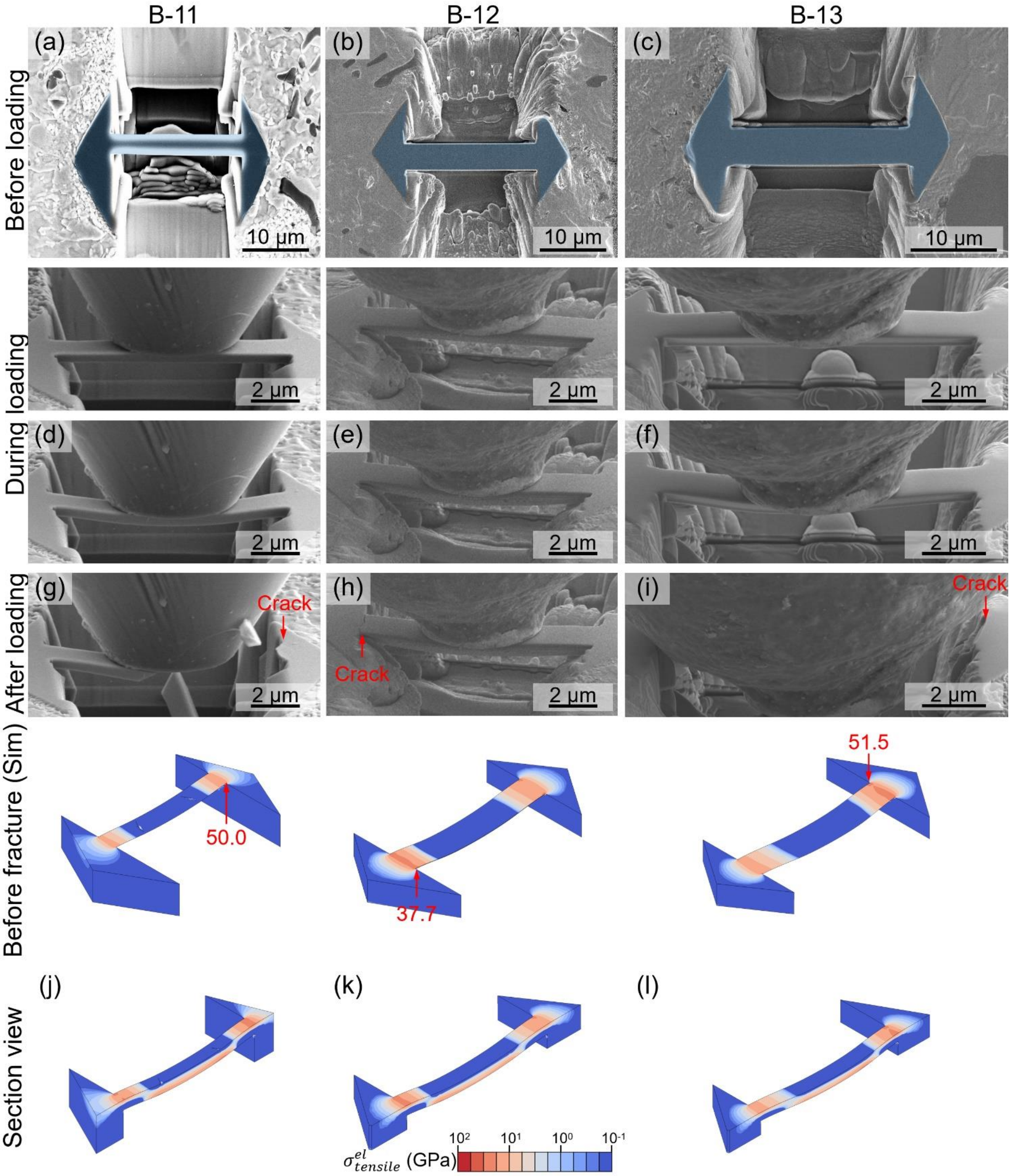

Supplementary Figure 8. Fixed end four-point bending of basal plane oriented TiB_2 beams (B-11 to B-13) and corresponding FEM stress fields. (a-c) SEM images of specimens B-11, B-12, and B-13 in the initial state prior to loading. In all three beams, the basal planes are oriented nearly parallel to the matrix surface and are marked in blue. (d-f) In situ SEM images acquired at the final stable loading increment immediately prior to failure, showing the deformed configurations under four-point

bending type loading with both ends effectively fixed. (g-i) Post-mortem SEM images after fracture, revealing the fracture surfaces. (j-l) Specimen specific finite element simulations reproducing the measured deformation profiles and showing the distribution of tensile (principal) stress on the tensile surface immediately prior to failure. Owing to the fixed end boundary condition, up to three competing tensile stress maxima can develop within a single beam: two near the fixed ends and one within the nominal constant moment region. In each case, the experimentally observed fracture origin coincides with the FEM predicted highest stress location.

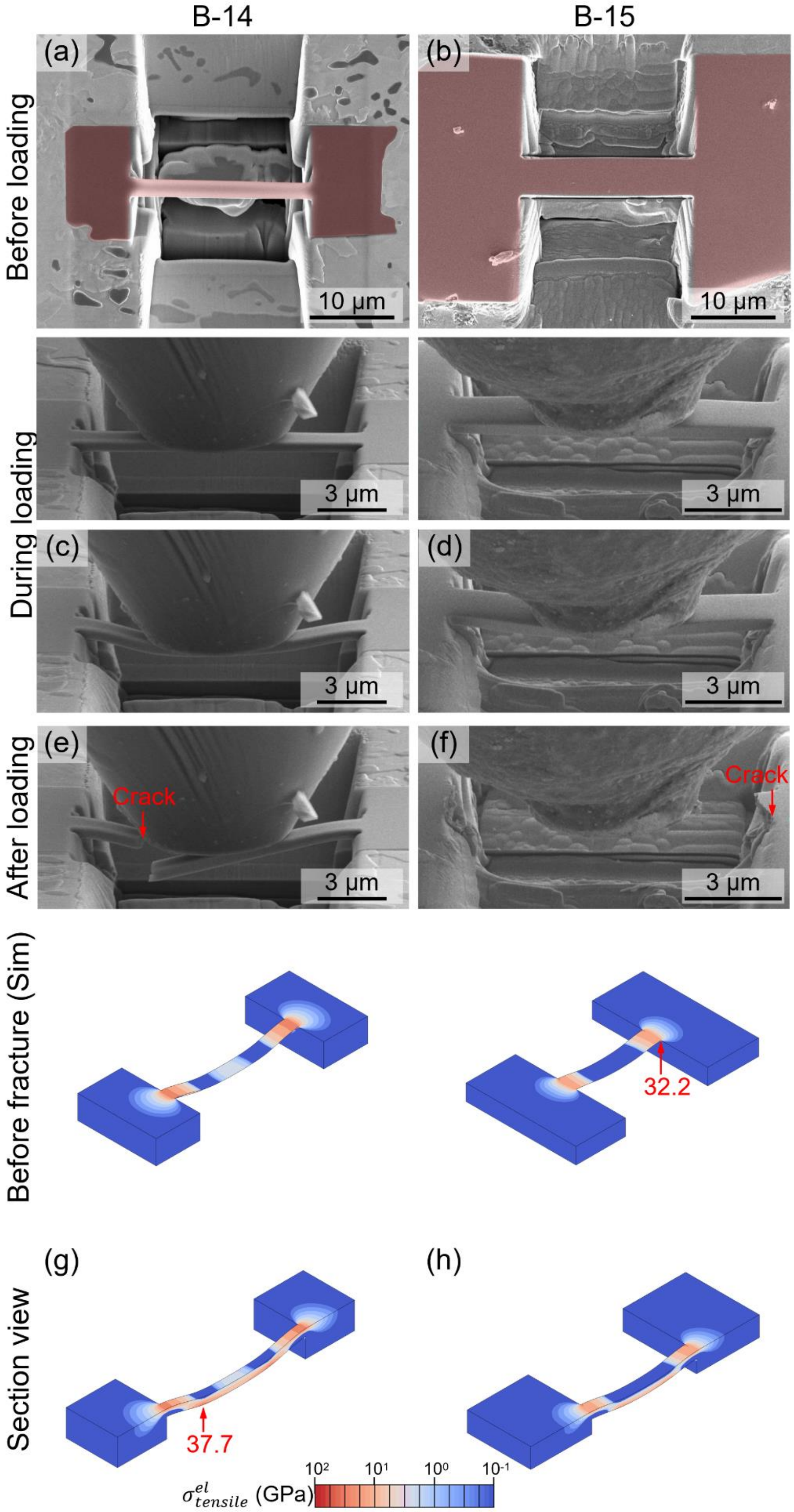

Supplementary Figure 9. Fixed end four-point bending of prismatic plane oriented TiB_2 beams (B-14 and B-15) and corresponding FEM stress fields. (a-b) SEM images of specimens B-14 and

B-15 in the initial state prior to loading. In both beams, the prismatic planes are oriented nearly parallel to the matrix surface and are marked in red. (c-d) In situ SEM images acquired at the final stable loading increment immediately prior to failure, showing the deformed configurations under four-point bending type loading with both ends effectively fixed. (e-f) Post-mortem SEM images after fracture, revealing the fracture surfaces. (g-h) Specimen specific finite element simulations reproducing the measured deformation profiles and showing the distribution of tensile (principal) stress on the tensile surface immediately prior to failure. Owing to the fixed end boundary condition, up to three competing tensile stress maxima can develop within a single beam: two near the fixed ends and one within the nominal constant moment region. In each case, the experimentally observed fracture origin coincides with the FEM predicted highest stress location.

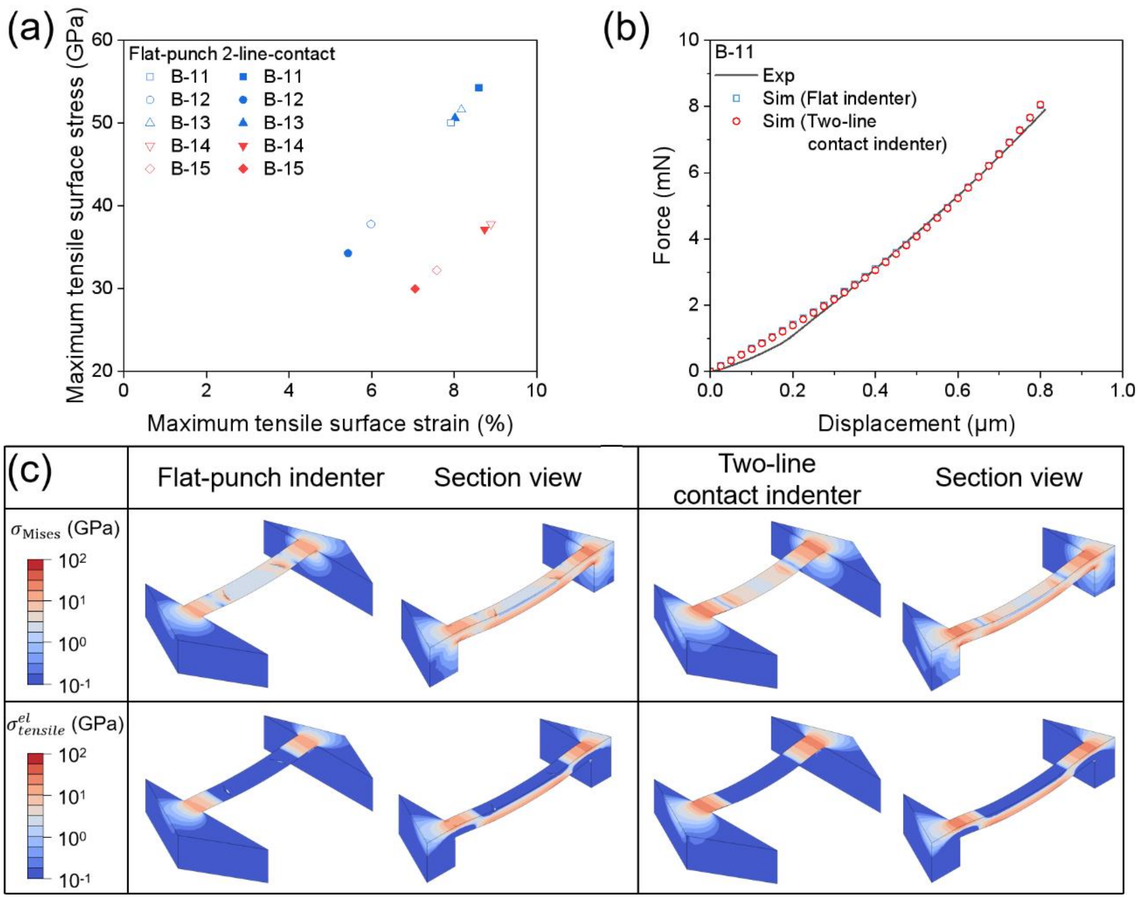

Supplementary Figure 10. FEM validation of the flat punch approximation for microscale four-point bending type loading. Finite element simulations confirm that the stress field generated by the flat punch loading configuration closely matches that of an idealized two-line contact indenter with the same contact spacing (equal to the flat punch diameter). (a) Comparison of the maximum tensile principal surface stress versus maximum tensile principal surface strain for specimens B-11 to B-15 extracted from simulations using the flat punch and two-line contact indenters, showing similar $\sigma_{\mathrm{I,max}}$ and $\varepsilon_{\mathrm{I,max}}$ values. (b) Simulated load-displacement curves for both indenter types compared with the experimental response, demonstrating similarly good agreement. (c) Representative stress fields (von Mises stress and tensile principal stress) immediately prior to failure for both indenter types, revealing nearly identical distributions and the same three competing tensile stress hot spots (two near the clamped ends and one within the nominal constant moment region).

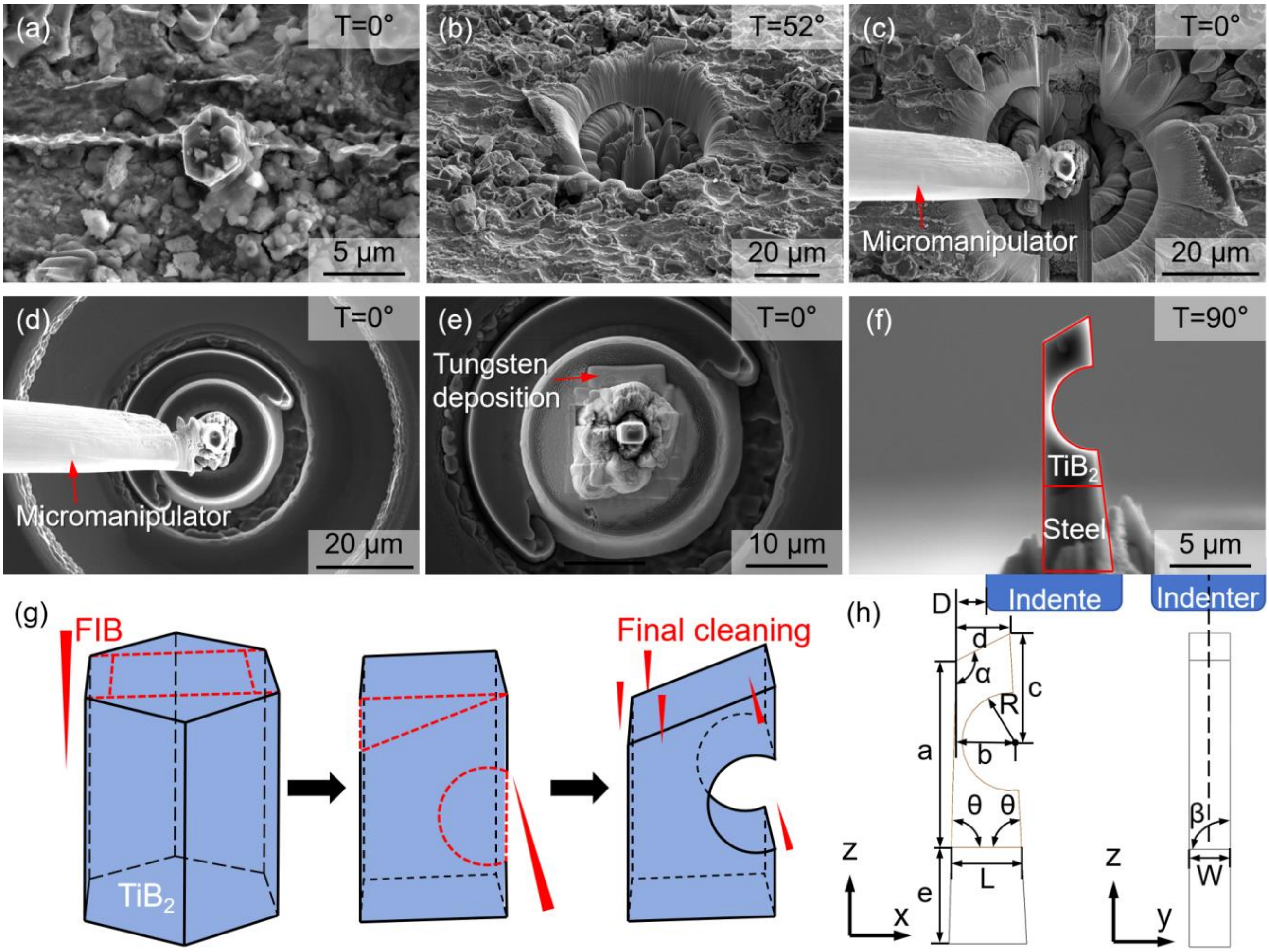

Supplementary Figure 11. Workflow for machining single crystal TiB_2 particles into C-shaped microstructures by FIB. (a) Identification of a single crystal TiB_2 particle partially embedded in the matrix and oriented perpendicular to the sample surface. (b) Removal of the surrounding matrix with a FIB beam normal to the surface. (c) Attachment of the matrix beneath the particle to a micromanipulator, followed by separation of the particle from the sample using FIB milling. (d) Transfer of the particle to a pre-machined cylindrical groove on a polished silicon block using the micromanipulator. (e) Rough shaping of the particle into a cuboid; tungsten was deposited beforehand to firmly secure the target during milling. (f) Introduction of a circular notch and a single sided roof using a FIB beam aligned parallel to the sample surface. (g) Final milling steps to complete the C-shaped geometry, summarizing steps (e) and (f). (h) Schematic front (left) and side (right) views of the C-shaped structure with geometric parameters.

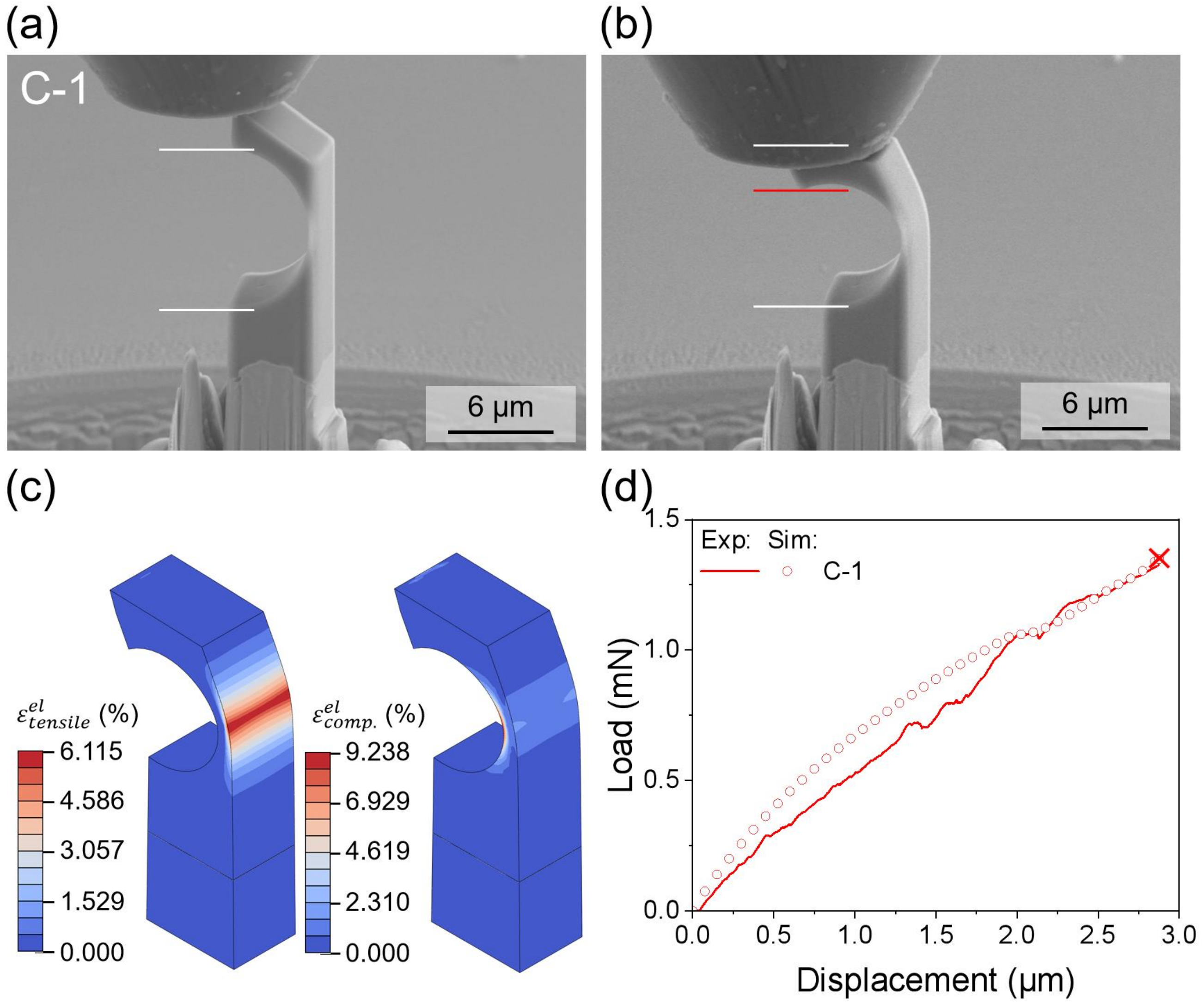

Supplementary Figure 12. High elastic deformation of a C-shaped structure. (a) C-shaped specimen C-1 in the initial state and (b) immediately prior to fracture. (c) Finite element (FE) simulation of the stress distribution corresponding to the state in (b). (d) Experimentally measured force–displacement curve (solid line) compared with FE simulation (symbol) for C-1.

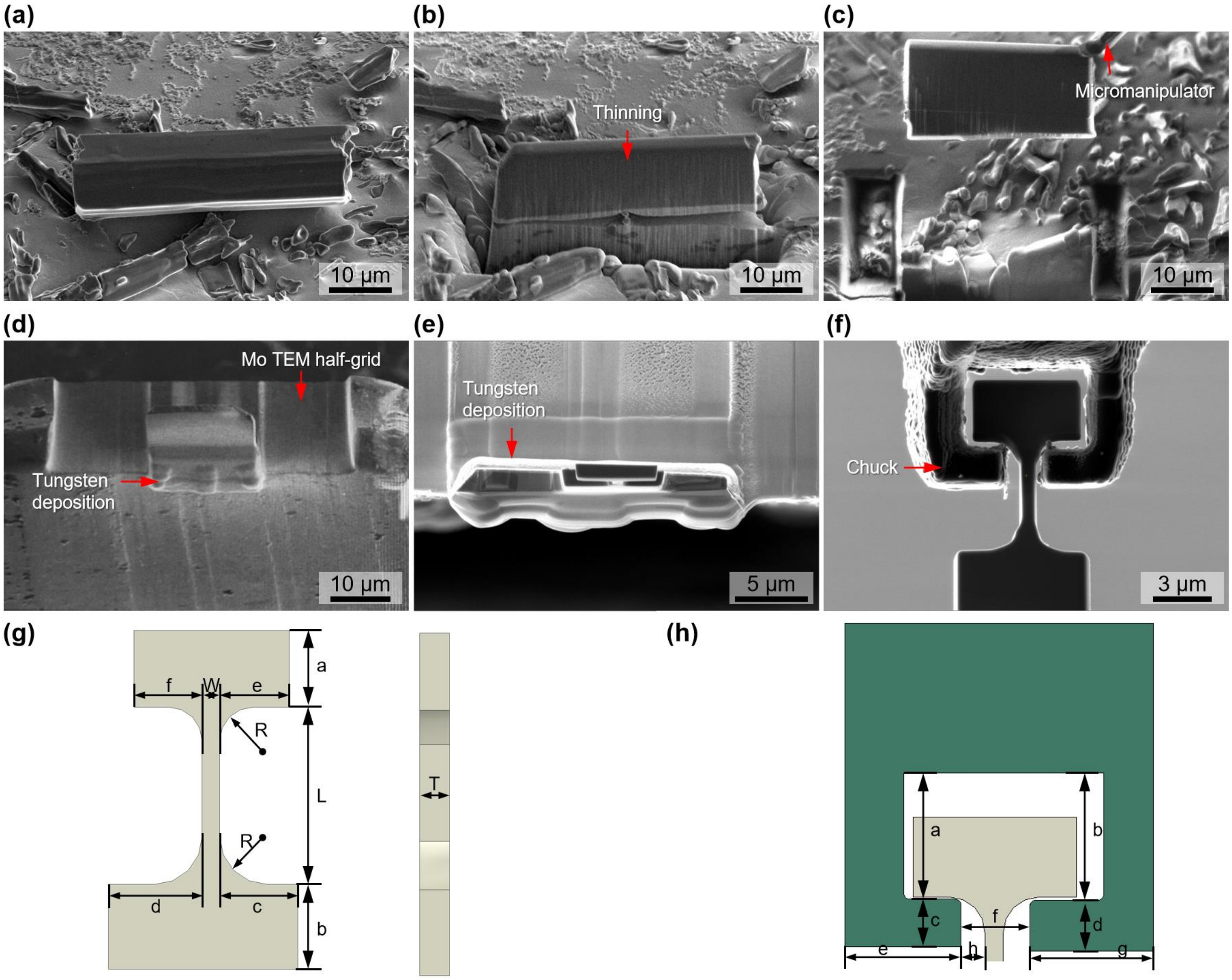

Supplementary Figure 13. Microfabrication process of the TiB_2 tensile samples. (a) SEM image of single crystal TiB_2 particle. (b) A thin lamella was milled using FIB. (c and d) The lamella was cut and transferred onto a Mo TEM half grid, then fixed by W welding. (e) The lamella was then cut into a tensile sample, with monitored by top view imaging. (f) SEM image of the tensile sample after it is connected to the gripper. (g) Schematic front (left) and top (right) views of the tensile sample with geometric parameters. (h) Schematic front views of the diamond gripper with geometric parameters.

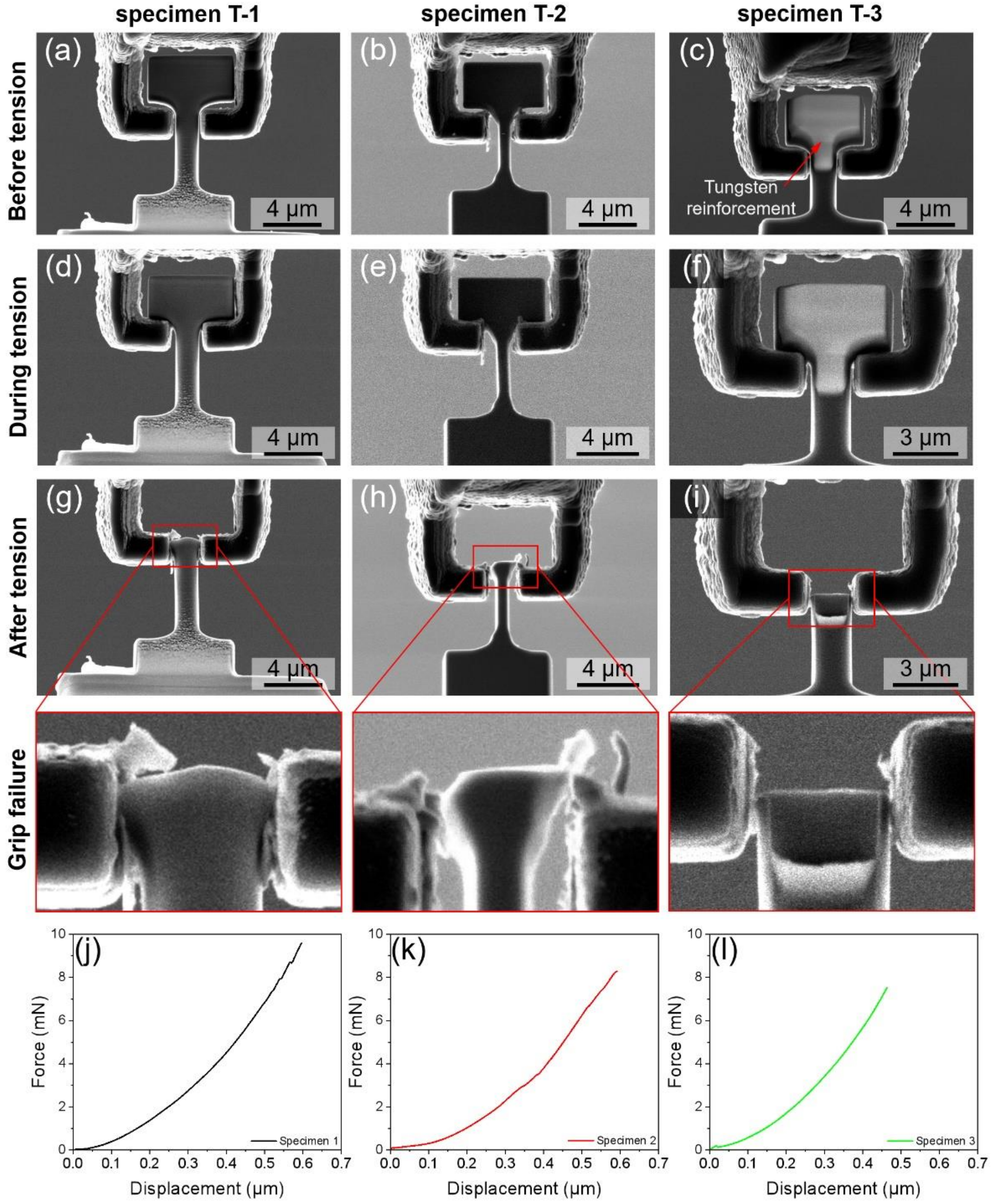

Supplementary Figure 14. Grip edge controlled failure in micrometer scale TiB_2 tensile tests and attempted mitigation strategies. SEM images of the three tensile specimens (T-1 to T-3) in (a-c) the initial state prior to loading, (d-f) during tension, and (g-i) after fracture. In all three tests, fracture initiates reproducibly at the grip edge rather than within the calibrated gauge section, as evidenced by the post-mortem fracture morphologies. (j-l) The corresponding experimental load-displacement curves. To mitigate grip edge failure, we explored two approaches: narrowing the gauge width to delay the onset of a critical grip edge stress concentration (specimen T-2), and reinforcing the gripped region by depositing a protective tungsten layer over the grip section (specimen T-3). Neither strategy eliminated the dominant grip edge stress concentration, and all specimens still failed at the grip edge.

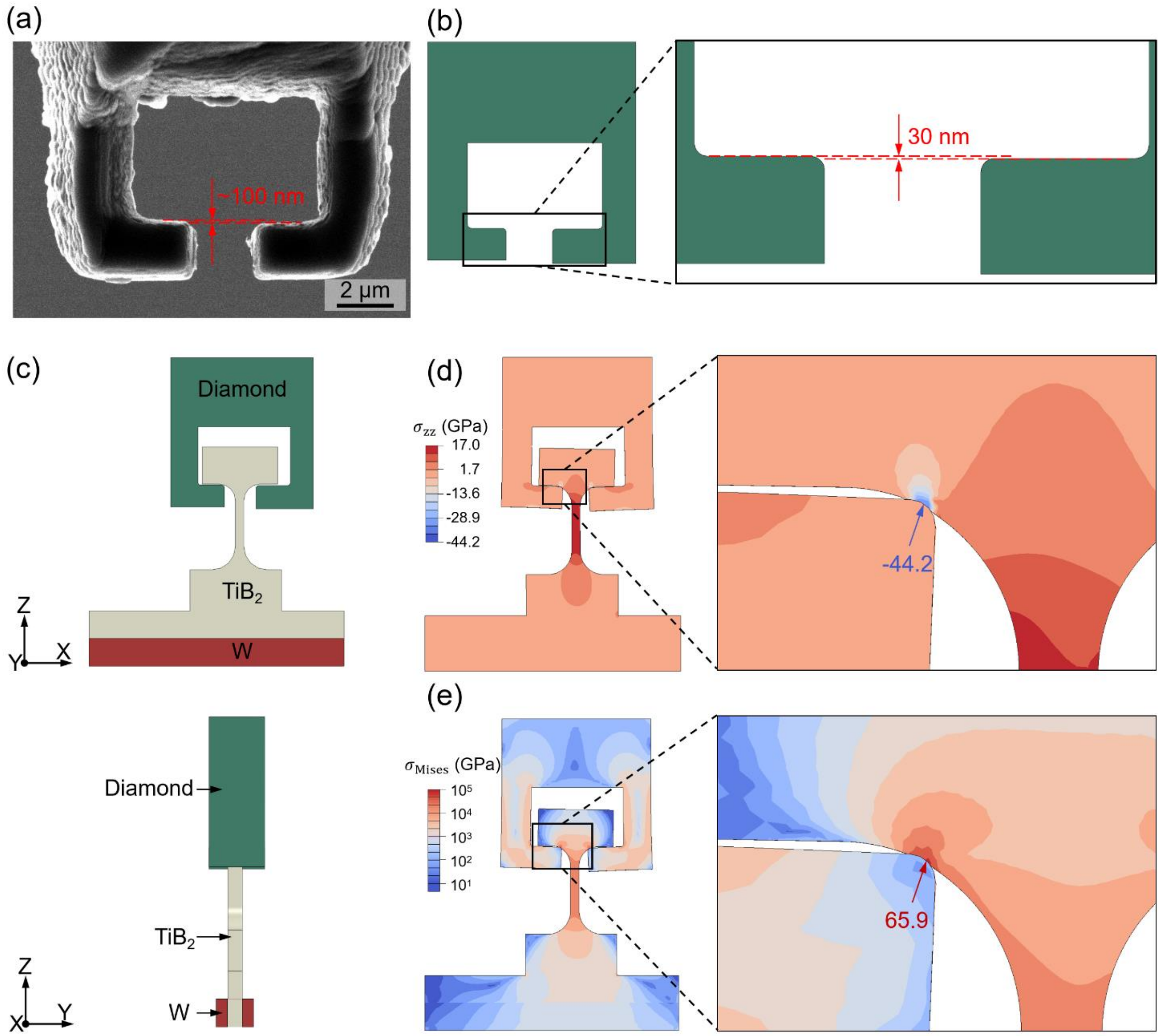

Supplementary Figure 15. FEM verification of grip induced stress concentration in the micrometer scale tensile specimen. (a) SEM image showing a slight misalignment between the two diamond gripping faces, on the order of ~100 nm. (b) Finite element model of the diamond gripper incorporating a smaller conservative misalignment of 30 nm to avoid overestimating the local grip edge stress concentration while still capturing the essential contact asymmetry. (c) One-to-one finite element model of tensile specimen T-2 used for the in situ tensile test. (d,e) Finite element simulations of T-2 immediately prior to fracture, showing (d) axial normal stress (tension/compression) and (e) von Mises stress distributions, highlighting the severe stress concentration at the grip edge.

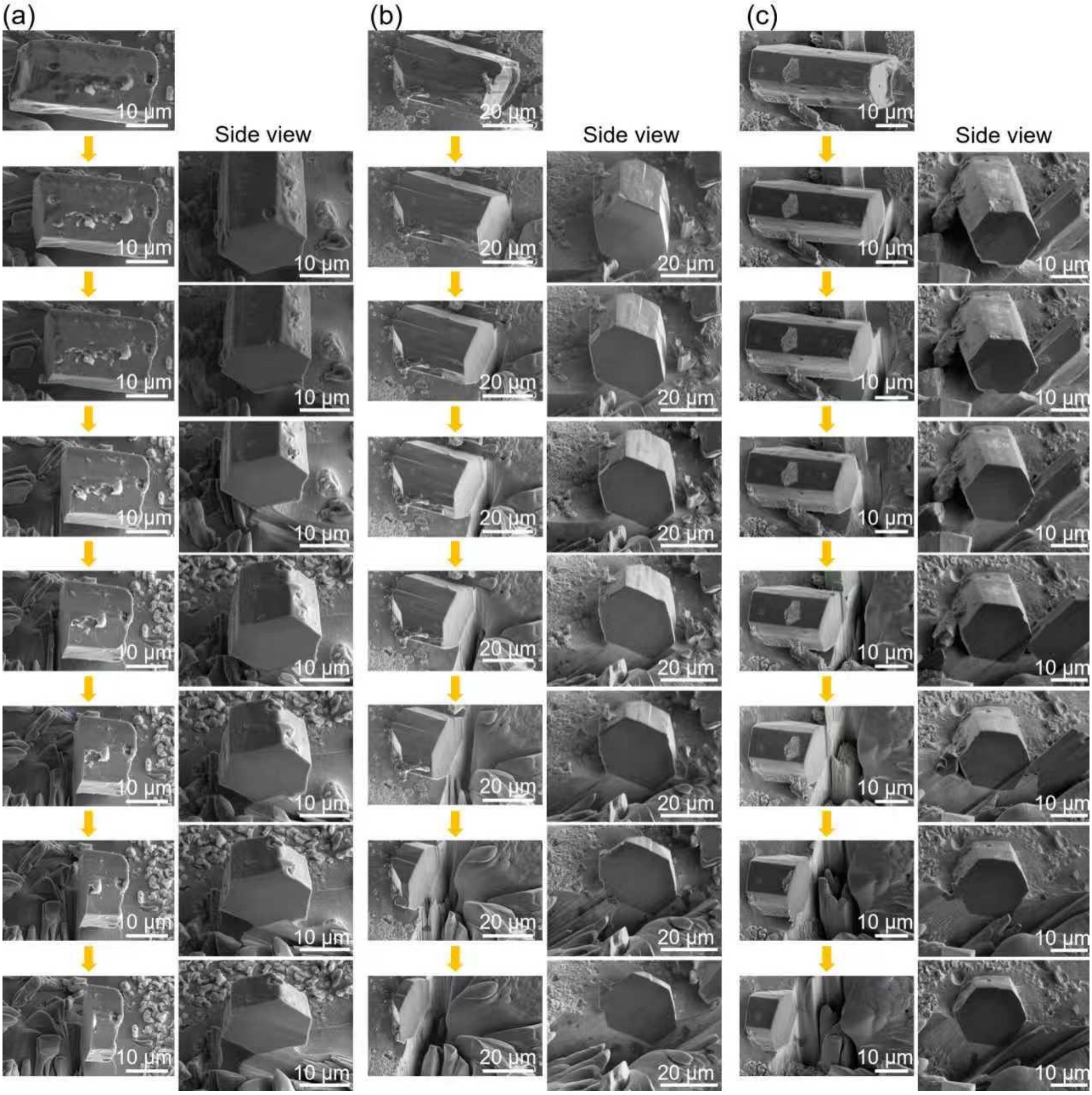

Supplementary Figure 16. Serial FIB cross sectioning confirms a dense, defect free interior in large TiB_2 particles. (a-c) Three representative large single crystal TiB_2 particles were sequentially sectioned by FIB along the [0001] direction. For each particle, the left row shows the newly exposed surface after successive sectioning steps and the right row shows the corresponding side view images of the cross section. No internal pores or microcracks are observed throughout the sectioned volumes. Any small irregularities are limited to the near surface region and are consistent with redeposition related roughness/edge faceting rather than intrinsic defects formed during eutectic growth.

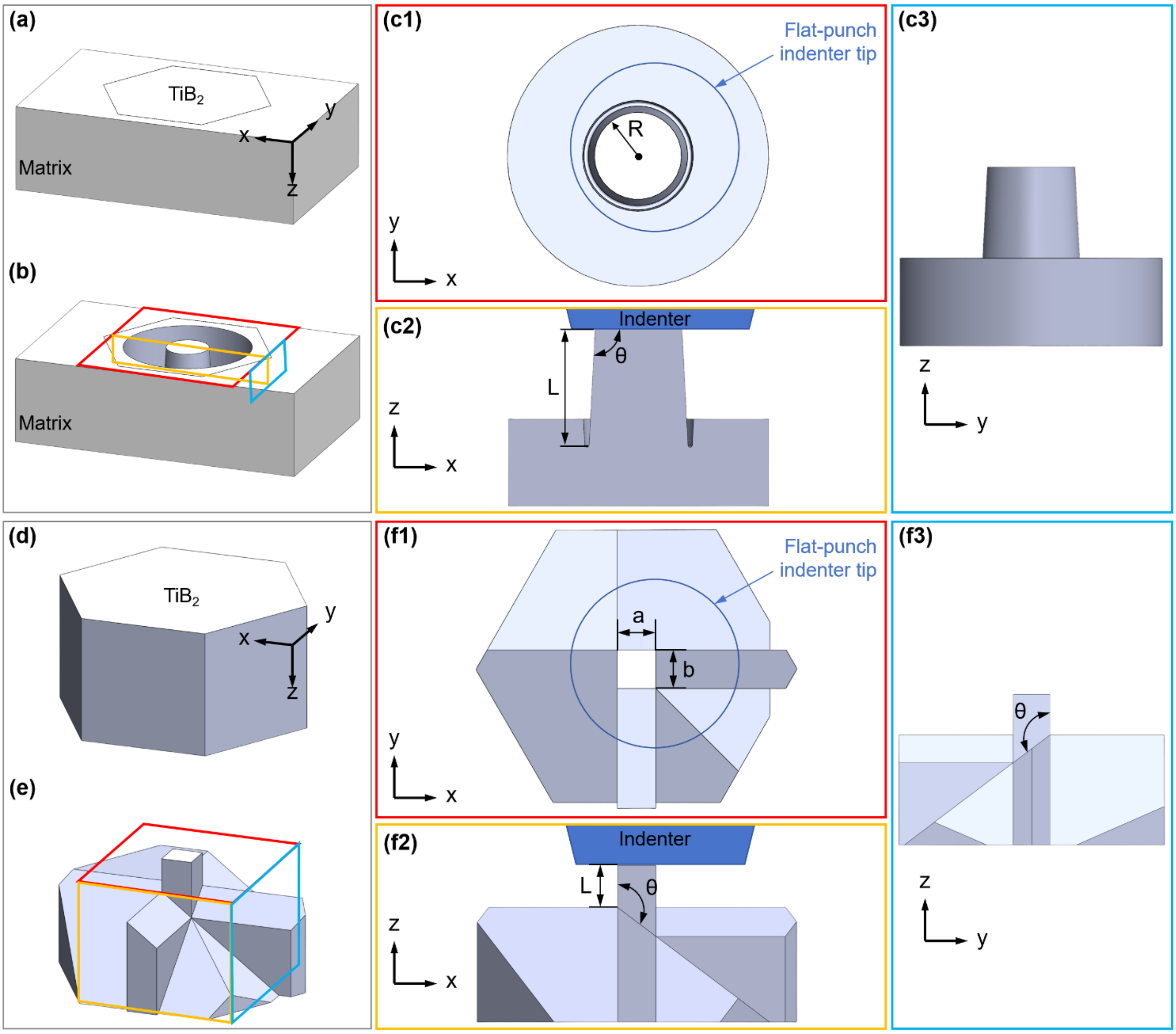

Supplementary Figure 17. Schematic of single crystal TiB_2 particles embedded in a steel matrix and the resulting micropillar geometries after FIB milling. (a,b) Cylindrical micropillar and (d,e) cuboid micropillar. (c1-c3) Top, front, and side views of the cylindrical pillar shown in (b); (f1-f3) corresponding views of the cuboid pillar shown in (e).

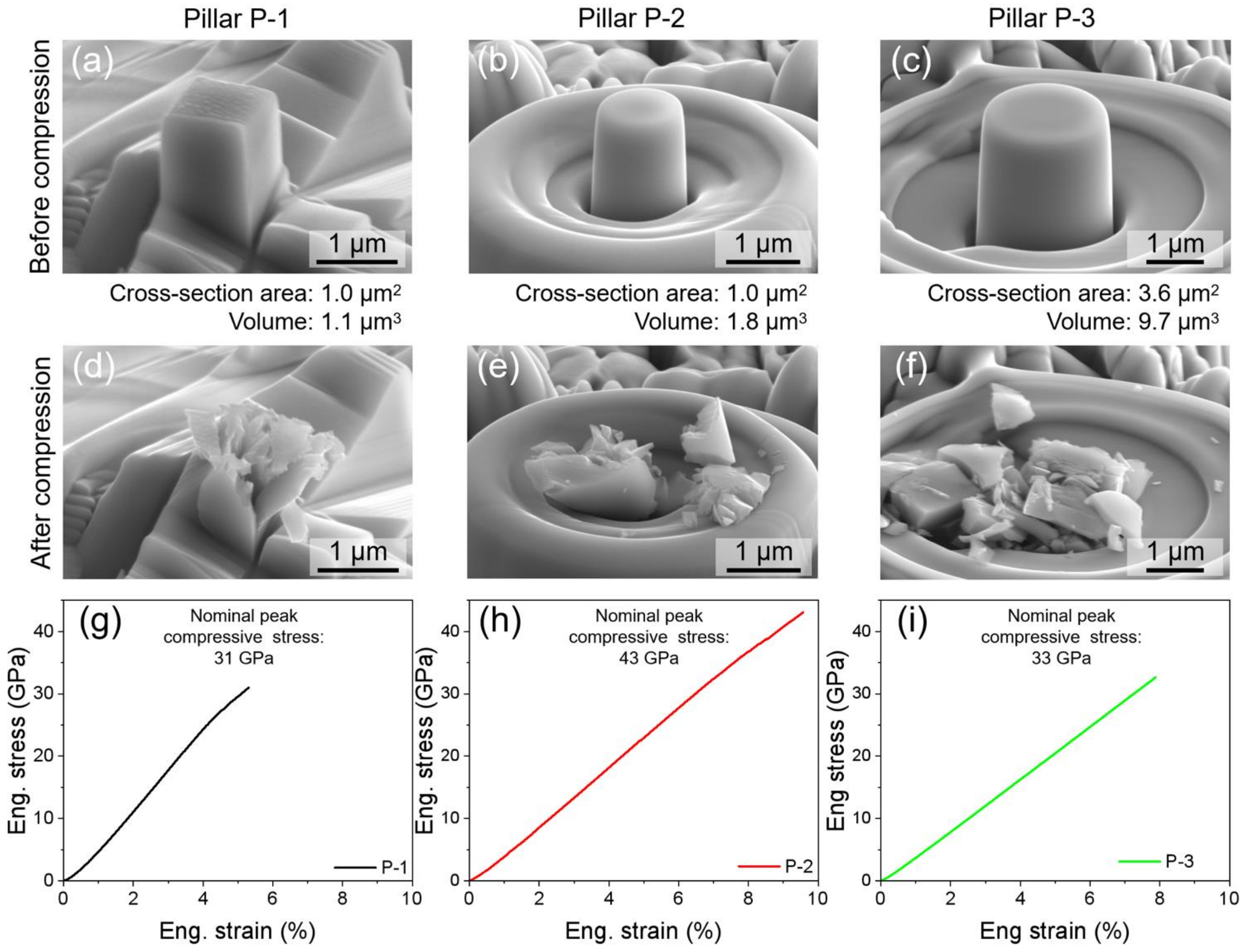

Supplementary Figure 18. Micropillar compression tests on micrometer scale single crystal TiB_2. (a-c) SEM images of the three FIB fabricated TiB_2 micropillars, P-1 to P-3, prior to loading. P-1 is a cuboid pillar, whereas P-2 and P-3 are cylindrical pillars. (d-f) SEM images of P-1 to P-3 after catastrophic fracture following compression. (g-i) Engineering (nominal) stress-strain curves for P-1 to P-3, respectively. The nominal peak compressive stresses are 31 GPa for P-1, 43 GPa for P-2, and 33 GPa for P-3.

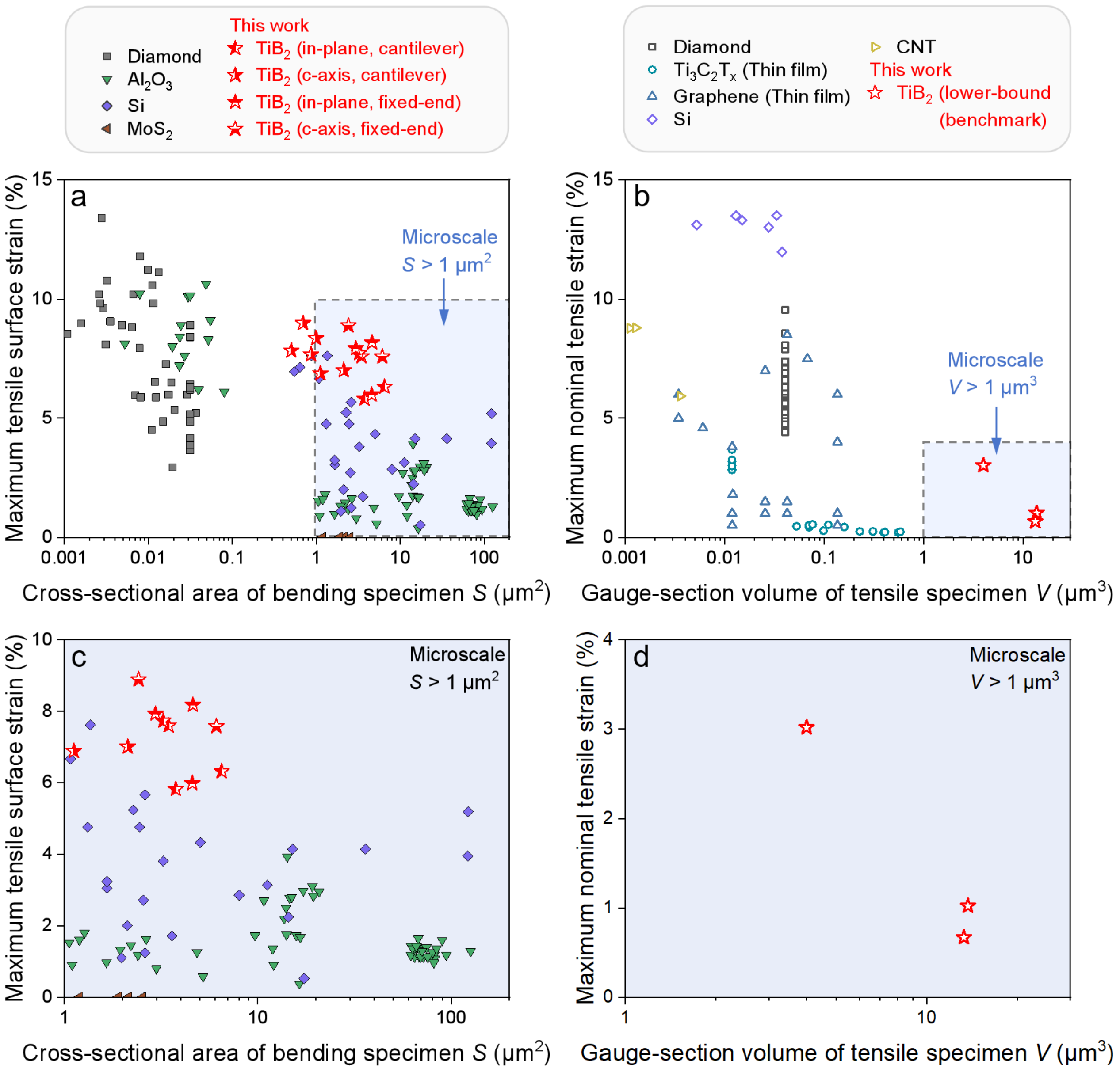

Supplementary Figure 19. Elastic strain limits of brittle solids as a function of specimen cross-sectional area, separated by loading mode to avoid mixing strain definitions. (a) Bending type datasets reported as the maximum tensile side surface strain at failure, extracted using the methodology of the original references (FEM or beam theory). Here, the cross-sectional area is used only as a geometric size descriptor, since the tensile strain is non-uniform over the cross section. (b) True uniaxial tensile datasets reported as nominal gauge fracture strain (or maximum uniform tensile strain prior to failure), plotted against gauge section volume. (c) Enlarged view of panel (a) for the micrometer scale regime with the x-axis starting at 1 μm^2. (d) Enlarged view of panel (b) for the micrometer scale regime with the x-axis starting at 1 μm^3. The literature sources and the corresponding loading modes are listed in Supplementary Table 10.

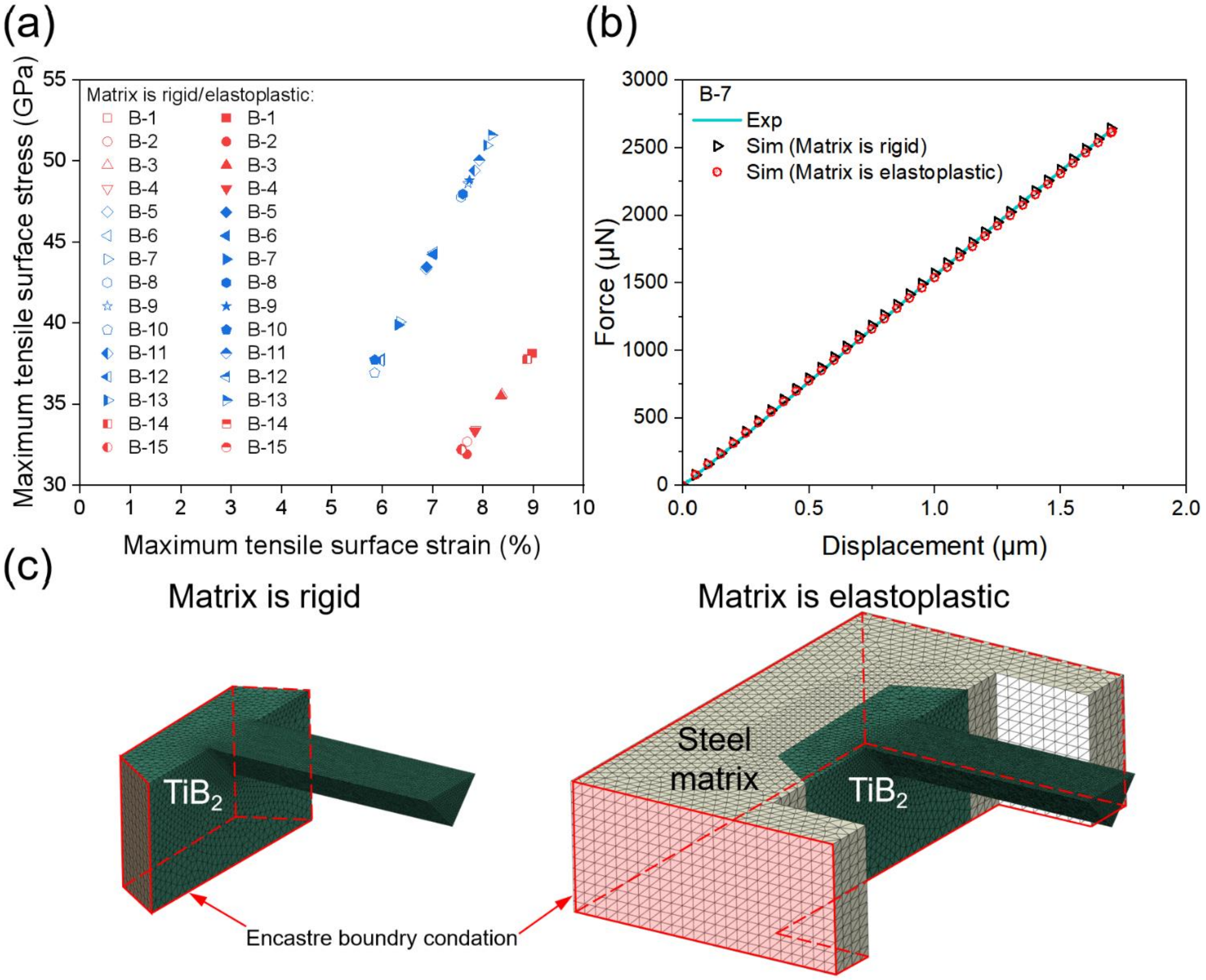

Supplementary Figure 20. Effect of matrix compliance on FEM extracted local maximum tensile stress and strain. (a) Comparison of $\sigma_{\mathrm{I,max}}$ and $\varepsilon_{\mathrm{I,max}}$ extracted from specimen specific FEM when the underlying matrix is modeled as rigid versus elastoplastic. (b) FE simulation results of the microcantilever specimens B-7 with different matrix properties. (c) Example of FE setup used in B-7. The matrix is modeled as isotropic elastoplastic with E = 168 GPa (HMS-1) or 210 GPa (HMS-2) and a yield strength of 700 MPa (HMS-1) or 350 MPa (HMS-2). Simulations showed no plastic deformation of the steel matrix during bending of the cantilevers, fixed end beams, and C-shaped structures. The resulting change in extracted $\sigma_{\mathrm{I,max}}$ and $\varepsilon_{\mathrm{I,max}}$ is within ±1%, forming one component of the error bars in Fig. 3.

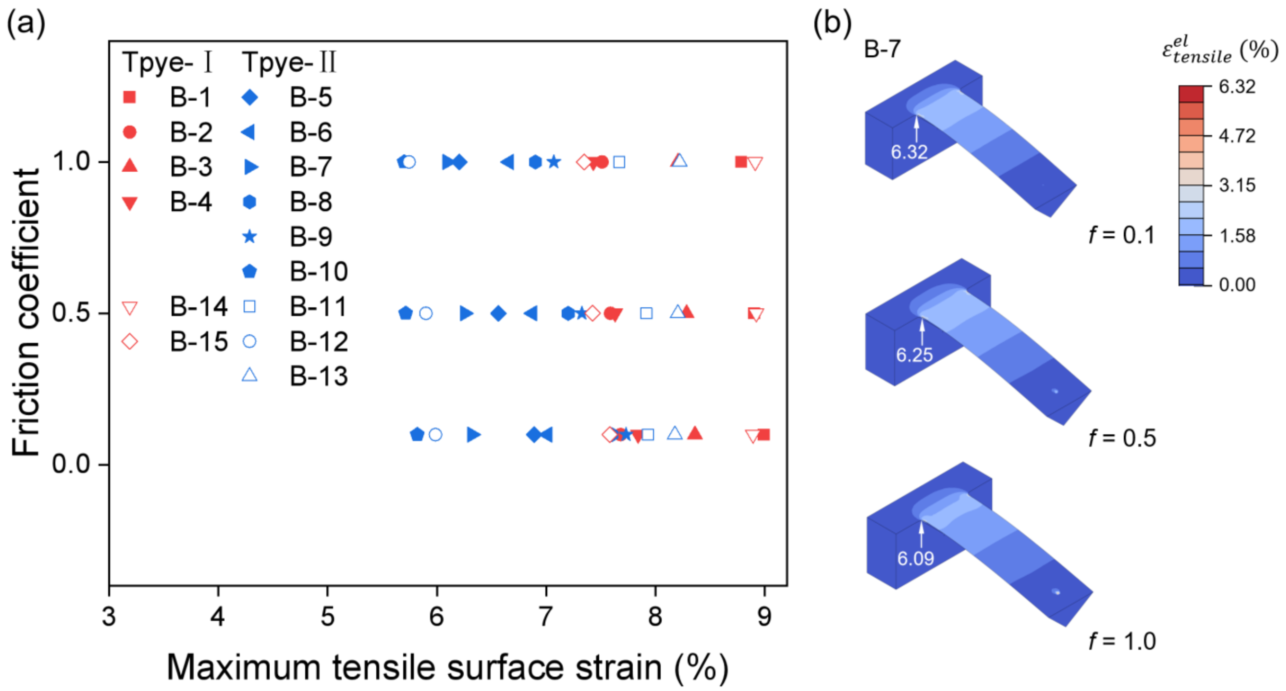

Supplementary Figure 21. Effect of indenter specimen friction coefficient on FEM extracted local maximum tensile strains. (a) $\varepsilon_{\mathrm{I,max}}$ extracted from specimen specific FEM using Coulomb friction coefficient f = 0.1, 0.5, and 1.0 at the indenter specimen interface. (b) Representative tensile principal strain fields for a microcantilever (B-7) at the experimentally applied displacement for different f. The resulting variation in extracted $\varepsilon_{\mathrm{I,max}}$ is within ±4%, forming one component of the error bars in Fig. 3.

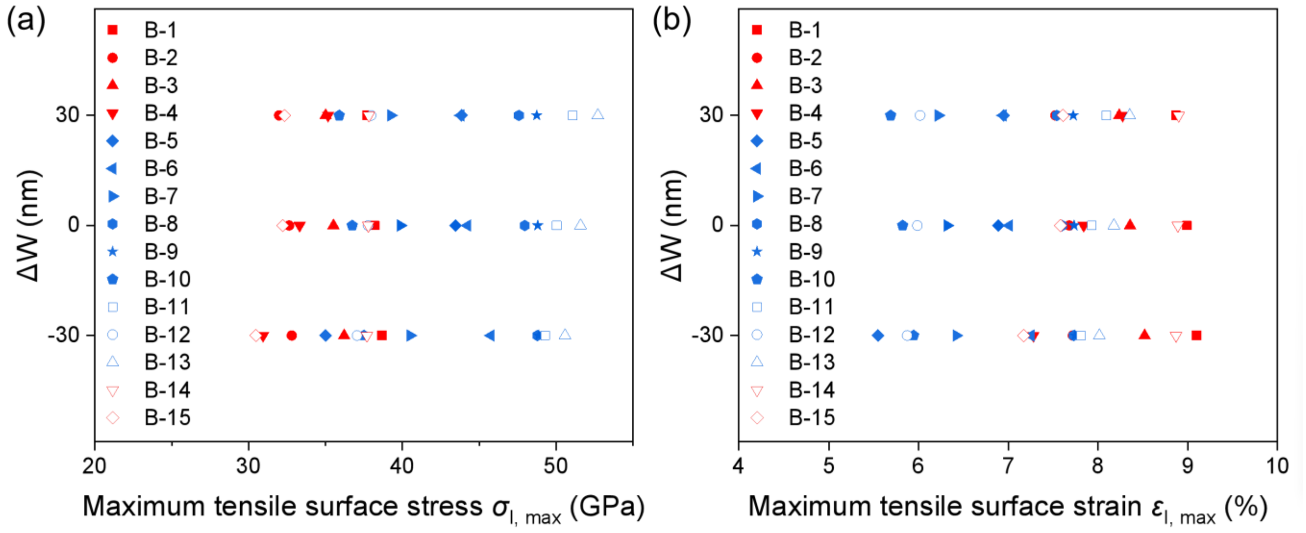

Supplementary Figure 22. Effect of SEM dimensional measurement uncertainty on FEM extracted local maximum tensile stress and strain. Sensitivity analysis in which the measured beam width (W) are independently perturbed by ±30 nm (SEM edge definition uncertainty), and the resulting changes in (a) $\sigma_{\mathrm{I,max}}$ and (b) $\varepsilon_{\mathrm{I,max}}$ are obtained through the specimen specific FEM. The resulting variation is within ±3%, forming one component of the error bars in Fig. 3.

Supplementary Table 1: Dimensions of FIB milled type I microcantilevers, listing geometric parameters L to γ (as defined in Supplementary Figure 2) and the distance D from the edge of the indenter to the fixed end of the microcantilever.

ID	L (μm)	W (μm)	a (μm)	b (μm)	θ (°)	α (°)	β (°)	γ (°)	D (μm)	Preparation method
B-1	5.10	1.18	0.00	0.00	27	0	90	0.0	3.8	Etching (HMS-1)
B-2	7.00	1.24	0.00	0.00	24	0	90	0.0	5.7	Etching (HMS-1)
B-3	5.80	1.22	0.63	0.44	47	3	76	6.6	4.6	Etching (HMS-1)
B-4	6.73	0.88	0.00	0.00	21	0	78	0.0	5.8	Etching (HMS-1)

Supplementary Table 2: Dimensions of FIB-milled type II microcantilevers, listing geometric parameters L to γ (as defined in Supplementary Figure 2) and the distance D from the edge of the indenter to the fixed end of the microcantilever.

ID	L (μm)	W (μm)	Unit vector of AB	θ (°)	β (°)	γ (°)	D (μm)	Preparation method
B-5	5.40	1.40	(0.067, 0.704, 0.707)	24	101	10	3.8	Etching (HMS-1)
B-6	7.10	1.90	(0,0,1)	23	90	0	5.3	Polishing (HMS-1)
B-7	16.30	5.10	(0,0,1)	45	90	0	13.1	Polishing (HMS-2)
B-8	10.58	3.72	(0,0,1)	45	90	0	9.6	Polishing (HMS-2)
B-9	14.49	3.60	(0,0,1)	46	90	0	13.4	Polishing (HMS-2)
B-10	16.80	3.94	(0,0,1)	45	90	0	16.0	Polishing (HMS-2)

Supplementary Table 3: Anisotropic material parameters of TiB_2 obtained from finite element (FE) inverse analysis of in situ nanoindentation data. Literature values from first principles calculations and prior experiments are included for comparison.

Parameters		This work	Other methods		
			Exp. [1]	DFT [2]	Exp. [3]
E_1	Young's modulus in c-axis (GPa)	425	407	432	454.5
E_2	Young's modulus in basal plane (GPa)	631	639	588	631.2
v_1	Poisson's ratio in c-axis	0.11			0.111
v_2	Poisson's ratio in basal plane	0.11			
G_1	Shear modulus in c-axis (GPa)	191			
G_2	Shear modulus in basal plane (GPa)	284		284.4	262.6
X_t	Maximum tensile surface stress in c-axis (GPa)	32~38			
X_c	Maximum compressive surface stress in c-axis (GPa)	35~40			
Y_t	Maximum tensile surface stress in basal plane (GPa)	38~49			
Y_c	Maximum compressive surface stress in basal plane (GPa)	40~51			

Supplementary Table 4: Dimensions of FIB milled four-point bending beams, listing geometric parameters L to D (as defined in Supplementary Figure 7) and the distance D from the edge of the indenter to the one end of the beam.

ID	L (μm)	W (μm)	a (μm)	θ (°)	D (μm)	Preparation method
B-11	19.95	3.17	0.00	50	5.30	Polishing (HMS-2)
B-12	19.98	3.87	0.05	59	4.65	Polishing (HMS-2)
B-13	21.25	4.00	0.00	60	6.60	Polishing (HMS-2)
B-14	21.4	2.36	0.00	49	5.45	Polishing (HMS-2)
B-15	21.1	4.60	0.00	60	5.45	Polishing (HMS-2)

Supplementary Table 5: Dimensions of FIB machined C-shaped structures, listing geometric parameters L to β as defined in Supplementary Figure 11h.

ID	L (μm)	W (μm)	a (μm)	b (μm)	c (μm)	d (μm)	e (μm)	R (μm)	θ (°)	α (°)	β (°)	D (μm)	Material
C-1	5.55	4.00	12.30	4.75	6.98	5.20	5.00	4.05	89.5	119.5	90.0	1.39	HMS-2
C-2	4.40	2.25	10.20	4.50	6.08	3.60	10.00	3.50	89.0	121.0	90.0	1.15	HMS-1

Supplementary Table 6: Dimensions of FIB machined tensile specimens, listing geometric parameters L to R as defined in Supplementary Figure 13.

ID	L (µm)	W (µm)	T (µm)	a (µm)	b (µm)	c (µm)	d (µm)	e (µm)	f (µm)	R (°)	Material
T-1	6.20	1.58	1.40	3.26	2.32	2.38	2.81	2.38	1.91	1.08	HMS-2
T-2	6.20	0.62	1.04	2.68	2.97	2.73	3.28	2.44	2.10	1.37	HMS-2
T-3	5.02	1.72	1.54	2.65	2.70	3.38	3.47	1.86	1.64	0.78	HMS-2

Supplementary Table 7: Dimensional parameters of the PFIB machined diamond gripper used for tensile specimens T-1 to T-3, including geometric parameters a-h and positioning parameters i-j as defined in Supplementary Figure 13h.

ID	a (μm)	b (μm)	c (μm)	d (μm)	e (μm)	f (μm)	g (μm)	h (μm)	i (μm)	j (μm)	Material
T-1	4.22	4.25	1.58	1.7	2.26	2.62	4.39	1.79	4.63	0.11	HMS-2
T-2	4.22	4.25	1.58	1.7	2.26	2.62	4.39	1.79	4.63	0.61	HMS-2
T-3	4.22	4.25	1.58	1.7	2.26	2.62	4.39	1.79	4.63	0.04	HMS-2

Supplementary Table 8: Dimensions of FIB milled micropillars, listing geometric parameters L to θ as defined in Supplementary Figure 17.

ID	L (μm)	a (μm)	b (μm)	R (μm)	θ (°)	Preparation method
P-1	1.10	1.00	1.00		127	Polishing (HMS-2)
P-2	1.81			1.14	92	Polishing (HMS-2)
P-3	2.68			2.14	92	Polishing (HMS-2)

Supplementary Table 9: Chemical compositions (wt.%) of the two high modulus steels, HMS-1 and HMS-2.

Composite	C+N	Si	Mn	Al	Ti	B	Fe
HMS-1	0.03	3.07	25.00	3.10	5.03	2.09	Bal.
HMS-2	0.03	0.00	0.00	0.00	5.70	2.50	Bal.

Supplementary Table 10: The present TiB_2 results and literature datasets compiled in Fig. 7 and Supplementary Fig. 19, including the specific stress/strain definition (e.g., local tensile side surface maxima in bending versus nominal gauge stress/strain in tension) and value, the cross-sectional area of specimen (*S*) or the gauge volume of specimen (*V*), and the loading mode (bending type versus true uniaxial tension).

Composition	σ_t (GPa)	ε_t (%)	S (μm^2)	V (μm^3)
TiB_2 (this work)	31.9 ~ 38.1 (local)	7.8 ~ 9.0 (local)	0.5 ~ 6.5 (Bending)	
TiB_2 (this work)	2.8 ~ 12.8 (nominal)	0.45 ~ 2.03 (nominal)		4.0~13.7 (Tension)
Diamond[4]	29.3 ~ 147.4 (local)	2.93 ~ 13.4 (local)	4.6×10^{-4} ~ 0.036 (Bending)	
Diamond[5]	40.1 ~ 98.1 (local)	3.6 ~ 8.9 (local)	0.031 (Bending)	
Diamond[6]	40.9 ~ 79.4 (nominal)	4.4 ~ 9.6 (nominal)		0.04 (Tension)
Graphene[7]	50.0 ~ 60.0 (nominal)	5.0 ~ 6.0 (nominal)		3.4×10^{-3} (Tension)
Graphene[8]	14.4 ~ 44.3 (nominal)	1.8 ~ 4.6 (nominal)		6.0×10^{-3} ~ 0.012 (Tension)
Graphene[9]	4.8 ~ 102.0 (nominal)	0.5 ~ 8.5 (nominal)		0.011 ~ 0.136 (Tension)
Carbon nanotubes[10]	35.0 ~ 98.0 (nominal)	14.7 ~ 49.0 (nominal)		2.9×10^{-4} ~ 3.5×10^{-3} (Tension)
$Ti_3C_2T_x$[11]	15.4 (nominal)	3.2 (nominal)		0.012 (Tension)
$Ti_3C_2T_x$[12]	0.04 ~ 0.7 (nominal)	0.2 ~ 2.3 (nominal)		0.098 ~ 0.11 (Tension)
Al_2O_3[13]	3.9 ~ 6.6 (local)	1.0 ~ 1.6 (local)	62 ~ 130 (Bending)	
Al_2O_3[14]	1.5 ~ 15.7 (local)	0.4 ~ 3.9 (local)	4.8 ~ 21 (Bending)	
Al_2O_3[15]	3.2 ~ 7.2 (local)	0.8 ~ 1.8 (local)	1.1 ~ 3.0 (Bending)	
Al_2O_3[16]	28.1 ~ 48.9 (local)	6.1 ~ 10.6 (local)	5.3×10^{-3} ~ 0.08 (Bending)	

Si[17]	1.1 ~ 11.9 (local)	0.5 ~ 5.7 (local)	2.5 ~ 120 (Bending)	
Si[18]	2.3 ~ 16.0 (local)	1.1 ~ 7.6 (local)	0.55 ~ 2.5 (Bending)	
Si[19]	14.7 ~ 20.0 (nominal)	12.0 ~ 13.1 (nominal)		5.2×10^{-3} ~ 0.012 (Tension)
MoS_2[20]	35 ~ 110 (local)	5.9 ~ 10.5 (local)	1.7×10^{-4} ~ 1.9×10^{-3} (Bending)	

Supplementary References

1. Spoor P, Maynard J, Pan M, Green DJ, Hellmann J, Tanaka T. Elastic constants and crystal anisotropy of titanium diboride. *Applied physics letters* **70**, 1959-1961 (1997).
2. Zhang X, Luo X, Li J, Hu P, Han J. The ideal strength of transition metal diborides TMB2 (TM=Ti, Zr, Hf): Plastic anisotropy and the role of prismatic slip. *Scripta Materialia* **62**, 625-628 (2010).
3. Okamoto NL, Kusakari M, Tanaka K, Inui H, Otani S. Anisotropic elastic constants and thermal expansivities in monocrystal CrB2, TiB2, and ZrB2. *Acta Materialia* **58**, 76-84 (2010).
4. Nie A, *et al.* Approaching diamond's theoretical elasticity and strength limits. *Nat Commun* **10**, 5533 (2019).
5. Banerjee A, *et al.* Ultralarge elastic deformation of nanoscale diamond. *Science* **360**, 300-302 (2018).
6. Dang C, *et al.* Achieving large uniform tensile elasticity in microfabricated diamond. *Science* **371**, 76-78 (2021).
7. Cao K, *et al.* Elastic straining of free-standing monolayer graphene. *Nat Commun* **11**, 284 (2020).
8. Feng S, *et al.* Experimentally measuring weak fracture toughness anisotropy in graphene. *Communications Materials* **3**, (2022).
9. Jaddi S, *et al.* Definitive engineering strength and fracture toughness of graphene through on-chip nanomechanics. *Nat Commun* **15**, 5863 (2024).
10. Peng B, *et al.* Measurements of near-ultimate strength for multiwalled carbon nanotubes and irradiation-induced crosslinking improvements. *Nature nanotechnology* **3**, 626-631 (2008).
11. Rong C, *et al.* Elastic properties and tensile strength of 2D Ti(3)C(2)T(x) MXene monolayers. *Nat Commun* **15**, 1566 (2024).
12. Firestein KL, *et al.* Young's Modulus and Tensile Strength of Ti(3)C(2) MXene Nanosheets As Revealed by In Situ TEM Probing, AFM Nanomechanical Mapping, and Theoretical Calculations. *Nano Lett* **20**, 5900-5908 (2020).
13. Žagar G, Pejchal V, Mueller MG, Rossoll A, Cantoni M, Mortensen A. The local strength of microscopic alumina reinforcements. *Acta Materialia* **100**, 215-223 (2015).
14. Pejchal V, Fornabaio M, Žagar G, Mortensen A. The local strength of individual alumina particles. *Journal of the Mechanics and Physics of Solids* **109**, 34-49 (2017).
15. Feilden E, *et al.* Micromechanical strength of individual Al2O3 platelets. *Scripta Materialia* **131**, 55-58 (2017).
16. Wang S, *et al.* An improved loop test for experimentally approaching the intrinsic strength of alumina nanoscale whiskers. *Nanotechnology* **24**, 285703 (2013).
17. Mueller MG, Fornabaio M, Žagar G, Mortensen A. Microscopic strength of silicon particles in an aluminium–silicon alloy. *Acta Materialia* **105**, 165-175 (2016).
18. Mueller MG, Žagar G, Mortensen A. In-situ strength of individual silicon particles within an aluminium casting alloy. *Acta Materialia* **143**, 67-76 (2018).
19. Zhang H, *et al.* Approaching the ideal elastic strain limit in silicon nanowires. *Science advances* **2**, e1501382 (2016).

20. Colas G, Serles P, Saulot A, Filleter T. Strength measurement and rupture mechanisms of a micron thick nanocrystalline MoS2 coating using AFM based micro-bending tests. *Journal of the Mechanics and Physics of Solids* **128**, 151-161 (2019).
