# Supplementary material for: Achieving near-theoretical strength and high elasticity in micrometer scale TiB2 ceramics
Source: Nat Commun. 2026 Jul 1;17:5672. doi: 10.1038/s41467-026-74750-1 (PMC13323769; doi:10.1038/s41467-026-74750-1)
Supplement: Supplementary file 2 — Description of Additional Supplementary Files [file 41467_2026_74750_MOESM2_ESM.pdf]

Description of Additional Supplementary Files

Supplementary Movie 1 | In-situ bending test of B-1 (Type I), with final fracture.

Supplementary Movie 2 | In-situ bending test of B-3 (Type I), with final fracture.

Supplementary Movie 3 | In-situ bending test of B-5 (Type II), with final fracture.

Supplementary Movie 4 | In-situ bending test of B-6 (Type II), with final fracture.

Supplementary Movie 5 | In-situ bending test of B-14 (Type I), with final fracture.

Supplementary Movie 6 | In-situ bending test of C-1 (shown in Supplementary Fig. 5), with final fracture.

Supplementary Movie 7 | In-situ loading-unloading test of C-2, with fully recoverable flexural deformation (shown in Fig. 4a, b). The extracted maximum local (tensile) elastic strain is the highest (~6.6%) among all C-shaped specimens.

Supplementary Movie 8 | In-situ tensile test of T-2, with final fracture.

These videos, presented at a speed six times faster than the originally recorded video (i.e., the real flat tip displacement rate is 5 nm/s) aim to show the different responses observed during the tests.
