# Supplementary material for: Achieving near-theoretical strength and high elasticity in micrometer scale TiB2 ceramics
Source: Nat Commun. 2026 Jul 1;17:5672. doi: 10.1038/s41467-026-74750-1 (PMC13323769; doi:10.1038/s41467-026-74750-1)
Supplement: Supplementary file 11 — Transparent Peer Review file [file 41467_2026_74750_MOESM11_ESM.pdf]

nature portfolio

Peer Review File

Achieving near-theoretical strength and high elasticity in micrometer scale TiB2 ceramics

Corresponding Author: Professor Wei Xu

This file contains all reviewer reports in order by version, followed by all author rebuttals in order by version.

Version 0:

Reviewer comments:

Reviewer #1

(Remarks to the Author)
I find the goal of reaching theoretical strength of materials very valuable. This work attempts to reach theoretical strength of TiB2 at the micrometer scale. This is a well-written paper. I found the in-situ synthesis of TiB2 particles and studying their properties by FIB sample lifting very interesting and novel. I think this is a clever way of synthesizing large TiB2 particles with minimal defects. However, my main concern with this article is the claim of approaching near theoretical strength values at the micrometer volumes. The authors employed bend testing on the FIB samples. Bend testing, by its nature, localizes the stresses to only very small volumes and the authors measure strength from the low volume regions. This will obviously increase the strength of the materials by minimizing the chances of finding defects in a smaller volume. The authors should perform small-scale tensile testing or 4-point bend testing to truly support their claims of approaching theoretical strength at the micrometer scale. I can not recommend publication of this paper without addition of new tests and data. I think this is the major promise of this paper and the current methodology of bend testing, unfortunately, does not provide enough evidence to support the main conclusion of this paper.

Besides my main concern of reporting strength values from bend testing, I have following questions and suggestions:
- Please provide microstructure characterization results from the TiB2 particles to prove that they are actually defect-free. I think FIB cross section of several particles will show us the defects like tiny crack or pores or their absence of in the TiB2 particles.
- I am not sure if it is correct to use cross-sectional area of a bend testing specimen to report the tensile fracture strain of that sample. Again, the tensile stresses are only concentrated at a small area on the top surface, and stresses are not equally distributed over the entire cross-section of the specimens reported in Figure 5. I am not sure about the data from the literature, but if they are from actual tensile tests, the comparison in figure 5 is not valid.
- What are the error bars in Figure 3 represent? My understanding is there are only 10 samples. Are the error bars in single sample correspond to measurement error?
- Please provide more details on the eutectic reactions forming the TiB2 particles in-situ. This is one of the novel parts of this paper but there are not enough details to reproduce this method.
- In videos of Samples B3 and B5, there is cracking near the bottom attachment point of the samples close the fracture. I think the samples should be considered as failed at this point, and the stresses at the crack initiation instance should be reported as the fracture strengths. Can you please clarify what is happening in these samples?

Reviewer #2

(Remarks to the Author)
The authors employ eutectic solidification in steel metallurgy to synthesize a rigid, micrometer-scale TiB_2 ceramic phase. Mechanical properties are evaluated through micrometer-scale bending tests and compared with measurements from cantilever and C-shaped specimens. The experiments are carefully conducted; however, several aspects of the interpretation require clarification.
1. The expression “tensile fracture strength” is potentially misleading. The tensile stress reported here is derived from FEM simulations of bending, rather than from direct tensile loading. Because bending introduces a complex stress state, the actual tensile component is uncertain and may be overestimated. It would be more accurate to describe the reported value as the “maximum surface stress estimated by FEM.”

2. Bending tests combined with FEM analysis have previously been applied to micro-sized diamond, revealing tensile strains up to ~9% and stresses of ~89–98 GPa (Science 360, 300–302, 2018). The authors should better articulate the novelty and significance of their findings on TiB_2 ceramics in comparison with these prior studies.
3. The term “ideal strength” is not appropriate in this context. It specifically refers to the theoretical strength of a defect-free crystal, which is not the case in the present work.

Version 1:

Reviewer comments:

Reviewer #1

(Remarks to the Author)
I thank the authors for their very detailed, elaborate, and well-written response letter. I think this is the best response letter I have ever seen. I really appreciate the new tests, data, and discussion. All my comments and suggestions are addressed successfully. I just have a minor question on the Supplementary note S2 and supplementary figure 5. I guess I did not understand the procedure, but to me the binned volume should be bigger than the cumulative stressed volume. A clarification regarding this issue will be helpful to have in the supplementary file.

Reviewer #2

(Remarks to the Author)
The revised manuscript addresses most of the reviewer’s comments; however, several critical issues remain unresolved:
1. Tensile testing methodology.
In the tensile experiments, fracture reproducibly initiates at the grip edge rather than within the gauge section. This behavior suggests that the specimen geometry is not optimal and should be modified, for example by increasing the gauge width to reduce stress concentration at the grips.
Under the current configuration, it is questionable whether the tensile results can reliably represent the intrinsic mechanical properties of the TiB_2 ceramic. Accordingly, the statement that “the peak von Mises stress at the grip edge reaches ~65.9 GPa immediately prior to fracture” is disputable. In fact, the specimen sustains a nominal tensile stress of only ~13 GPa before fracture, which is significantly lower than the stress measured in bending tests (>30 GPa, Fig. 3).
2. Need for compression testing.
Given the uncertainties associated with the tensile results, compression testing is recommended. Compared with bending, compression provides a less complex stress state and would allow a more reliable assessment of the mechanical properties of the TiB_2 ceramic.

Version 2:

Reviewer comments:

Reviewer #1

(Remarks to the Author)
I thank the authors once again for their detailed response. My question is answered and addressed. I don't have any further comments or questions.

Reviewer #2

(Remarks to the Author)
The revision has addressed my concerns, and publication is recommended.

Open Access This Peer Review File is licensed under a Creative Commons Attribution 4.0 International License, which permits use, sharing, adaptation, distribution and reproduction in any medium or format, as long as you give appropriate credit to the original author(s) and the source, provide a link to the Creative Commons license, and indicate if changes were made.
In cases where reviewers are anonymous, credit should be given to 'Anonymous Referee' and the source.
The images or other third party material in this Peer Review File are included in the article's Creative Commons license, unless indicated otherwise in a credit line to the material. If material is not included in the article's Creative Commons license and your intended use is not permitted by statutory regulation or exceeds the permitted use, you will need to obtain permission directly from the copyright holder.
To view a copy of this license, visit https://creativecommons.org/licenses/by/4.0/

Response to Reviewers

Dear Editor and Reviewers,

We are grateful for your reviews and feedback on our manuscript NCOMMS-25-71116 "Approaching ideal strength and ultra-large elasticity in micrometer-scale TiB_2 ceramics". We are happy to submit a revised manuscript addressing your comments. The reviewers have each raised several points and we are sure that by addressing these points the manuscript has been significantly improved.

Each of the reviewers' comments are copied below in *italic*. Our responses are in blue, and revision to the manuscript are highlighted in red.

Reviewer #1:

General comment: *I find the goal of reaching theoretical strength of materials very valuable. This work attempts to reach theoretical strength of* TiB_2 *at the micrometer scale. This is a well-written paper. I found the in-situ synthesis of* TiB_2 *particles and studying their properties by FIB sample lifting very interesting and novel. I think this is a clever way of synthesizing large* TiB_2 *particles with minimal defects. However, my main concern with this article is the claim of approaching near theoretical strength values at the micrometer volumes.*

Response: Thank you for your positive opinion and for recognizing the novelty and value of our work. We also appreciate your careful evaluation of our central claim regarding near-theoretical strength at micrometer-scale volumes. To directly address this concern, we have performed extensive additional experiments and analyses, as detailed below.

Comment 1: *The authors employed bend testing on the FIB samples. Bend testing, by its nature, localizes the stresses to only very small volumes and the authors measure strength from the low volume regions. This will obviously increase the strength of the materials by minimizing the chances of finding defects in a smaller volume. The authors should perform small-scale tensile testing or 4-point bend testing to truly support their claims of approaching theoretical strength at the micrometer scale. I can not recommend publication of this paper*

without addition of new tests and data. I think this is the major promise of this paper and the current methodology of bend testing, unfortunately, does not provide enough evidence to support the main conclusion of this paper.

Response: Thank you for this very important point. We fully agree with the core concern: brittle fracture can exhibit a “weakest-link” effect, and because bending produces a non-uniform stress field, it is essential to consider the stressed volume when interpreting strength measured from micro-bending tests. In fact, **this consideration applies universally to all the bending-based strength measurements in the prior literature**. To directly address this point, **we followed the reviewer’s suggestion and performed (i) micrometer-scale tensile tests, and (ii) micrometer-scale four-point bending tests**, as described below and incorporated into the revised manuscript and Supplementary Information.

Before discussing these additional experiments, we would like to clarify why our original bending results are significant and distinct from most prior nanostructure studies. Bending (combined with FEM) has been the most widely used approach to probe ultrahigh strength in nanostructures (e.g., Science 360, 300–302, 2018; Nat. Commun. 10, 5533, 2019). However, in these works the tested specimens typically have **nanometer-scale cross-sections**, even when their lengths are micrometric. Importantly, in bending the material volume subjected to high tensile stress is governed primarily by the cross-section, rather than by the specimen length. **Increasing cross-sectional area increases the volume exposed to high tensile stress** and therefore increases the probability of sampling a critical flaw (i.e., imposes a stronger weakest-link constraint). This is precisely why increasing specimen cross-section is necessary when claiming “large-volume” near-theoretical strength, and it **motivates our emphasis on micrometer-scale cross-sections** in the present work. To make this stressed-volume issue explicit and quantitative, we performed a quantitative stressed-volume analysis based on the FE stress field for the beam B-9 in Fig. 2, showing that the TiB_2 volume experiencing very high tensile stress (e.g., > 15 GPa) already **exceeds 5 μm^3**. This supports the conclusion that the in-situ grown TiB_2 particles contain very few (if any) critical, strength-limiting defects

even at micrometer-scale stressed volumes. In short, the key advance here is a true increase in characteristic specimen size (cross-section/stressed volume), rather than micrometer length alone. We also clarify that, in the revised manuscript, our bending-derived results are compared only with bending-derived literature data (Fig. 7a), ensuring a direct and methodologically consistent comparison.

Regarding the new experiments, to the best of our knowledge, true micrometer-scale tension and four-point bending tests on brittle ceramics are rarely reported, especially the micrometer-scale tension (**absent from the literature**). Through our attempts we found that implementing these idealized configurations at the microscale is exceptionally challenging, and the **realized boundary conditions can deviate from textbook assumptions**. Consequently, these tests **do not yield a perfectly "clean" measurement of a large-volume uniaxial tensile fracture strength** in the strictest sense.

Nevertheless, **they provide highly relevant and mechanistically decisive evidence for the reviewer's key question**. Specifically, the new data show that: (i) in microscale tension, **the micrometer-scale gauge section sustains very high, nearly uniform tensile stress without fracture** prior to grip-edge failure, demonstrating an exceptionally low defect population within a large stressed volume; and (ii) in microscale four-point bending, multiple spatially separated tensile-stress hot spots exist within a single specimen, creating several competing sites where a critical defect (if present) could trigger fracture. The **one-to-one correspondence between the FE-predicted maximum tensile-stress locations and the experimentally observed fracture origins across all specimens** supports a stress-controlled failure criterion, rather than failure governed by randomly distributed critical defects, again consistent with extremely low flaw populations in micrometer-scale TiB_2.

We describe these new experiments and analyses in detail below, and the corresponding figures and discussion have been incorporated into the revised manuscript and Supplementary Information.

(1) Micrometer-scale tensile tests:

Following the reviewer's suggestion, we firstly conducted microscale tensile testing of single-crystal TiB_2. Because commercial fixtures for tensile testing of **micrometer-sized** brittle ceramics are not available, we PFIB-machined a custom diamond tensile gripper (Supplementary Fig. 13f; Supplementary Table 7), and prepared three single-crystal TiB_2 tensile specimens by lamella lift-out and FIB shaping (Supplementary Fig. 13a-e; Supplementary Table 6).

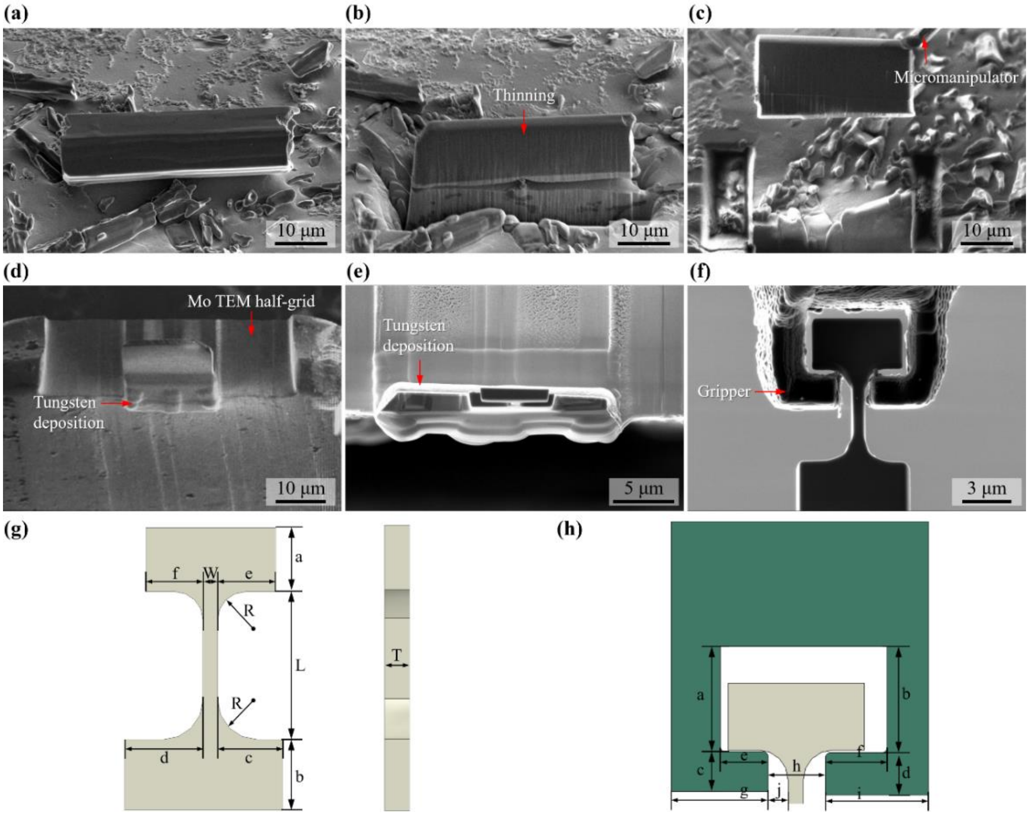

Supplementary Figure 13: Microfabrication process of the TiB_2 tensile samples. (a) SEM image of single crystal TiB_2 particle. (b) A thin lamella was milled using FIB. (c and d) The lamella was cut and transferred onto a Mo TEM half-grid, then fixed by W welding. (e) The lamella was then cut into a tensile sample, with monitored by top-view imaging. (f) SEM image of the tensile sample after it is connected to the gripper. (g) Schematic front (left) and top (right) views of the tensile sample with geometric parameters. (h) Schematic front views of the diamond gripper with geometric parameters.

Supplementary Table 6: Dimensions of FIB-machined tensile specimens, listing geometric parameters L to R as defined in Supplementary Figure 13.

ID	L (μm)	W (μm)	T (μm)	a (μm)	b (μm)	c (μm)	d (μm)	e (μm)	f (μm)	R (°)	Material
T-1	6.20	1.58	1.40	3.26	2.32	2.38	2.81	2.38	1.91	1.08	HMS-2
T-2	6.20	0.62	1.04	2.68	2.97	2.73	3.28	2.44	2.10	1.37	HMS-2
T-3	5.02	1.72	1.54	2.65	2.70	3.38	3.47	1.86	1.64	0.78	HMS-2

Supplementary Table 7: Dimensional parameters of the PFIB-machined diamond gripper used for tensile specimens T-1 to T-3, including geometric parameters a-h and positioning parameters i-j as defined in Supplementary Figure 13h.

ID	a (μm)	b (μm)	c (μm)	d (μm)	e (μm)	f (μm)	g (μm)	h (μm)	i (μm)	j (μm)	Material
T-1	4.22	4.25	1.58	1.7	2.26	2.62	4.39	1.79	4.63	0.11	HMS-2
T-2	4.22	4.25	1.58	1.7	2.26	2.62	4.39	1.79	4.63	0.61	HMS-2
T-3	4.22	4.25	1.58	1.7	2.26	2.62	4.39	1.79	4.63	0.04	HMS-2

In all three tests, **fracture occurred reproducibly at the grip edge rather than within the calibrated gauge section** (Supplementary Fig. 14), indicating that the measured failure is governed by unavoidable grip-induced stress concentrations rather than the intrinsic tensile strength of the gauge. This conclusion is supported by both the SEM fracture morphologies and the similar maximum loads sustained by the specimens despite their different cross-sectional areas. To diagnose the origin, we built an FE model of the as-fabricated specimen-gripper assembly (Supplementary Fig. 15). The simulation reveals a severe stress concentration at the grip edge: for specimen T-2, the peak Von Mises stress at the grip edge reaches ~**65.9 GPa** immediately prior to fracture, while the gauge region sustains a nearly uniform tensile stress of ~**13 GPa**. Because the maximum load is similar across specimens (Supplementary Fig. 14j-l), the uniform gauge stress decreases with increasing cross-sectional area.

This severe stress concentration arises from the hard contact between the diamond gripping corner and the TiB_2 grip section, and is further complicated by an unavoidable sub-micron height offset between the two diamond gripping faces (a gap of several tens of nanometers). At this length scale, even such small misalignment strongly affects the contact condition and **cannot be eliminated when machining/aligning diamond micro-grippers**. To mitigate grip-edge failure, we explored two approaches: narrowing the gauge width to delay the onset of a critical grip-edge stress concentration (specimen T-2), and reinforcing the gripped region by depositing a protective tungsten layer over the grip section (specimen T-3). Neither strategy eliminated the dominant grip-edge stress concentration, and all specimens still failed at the grip edge.

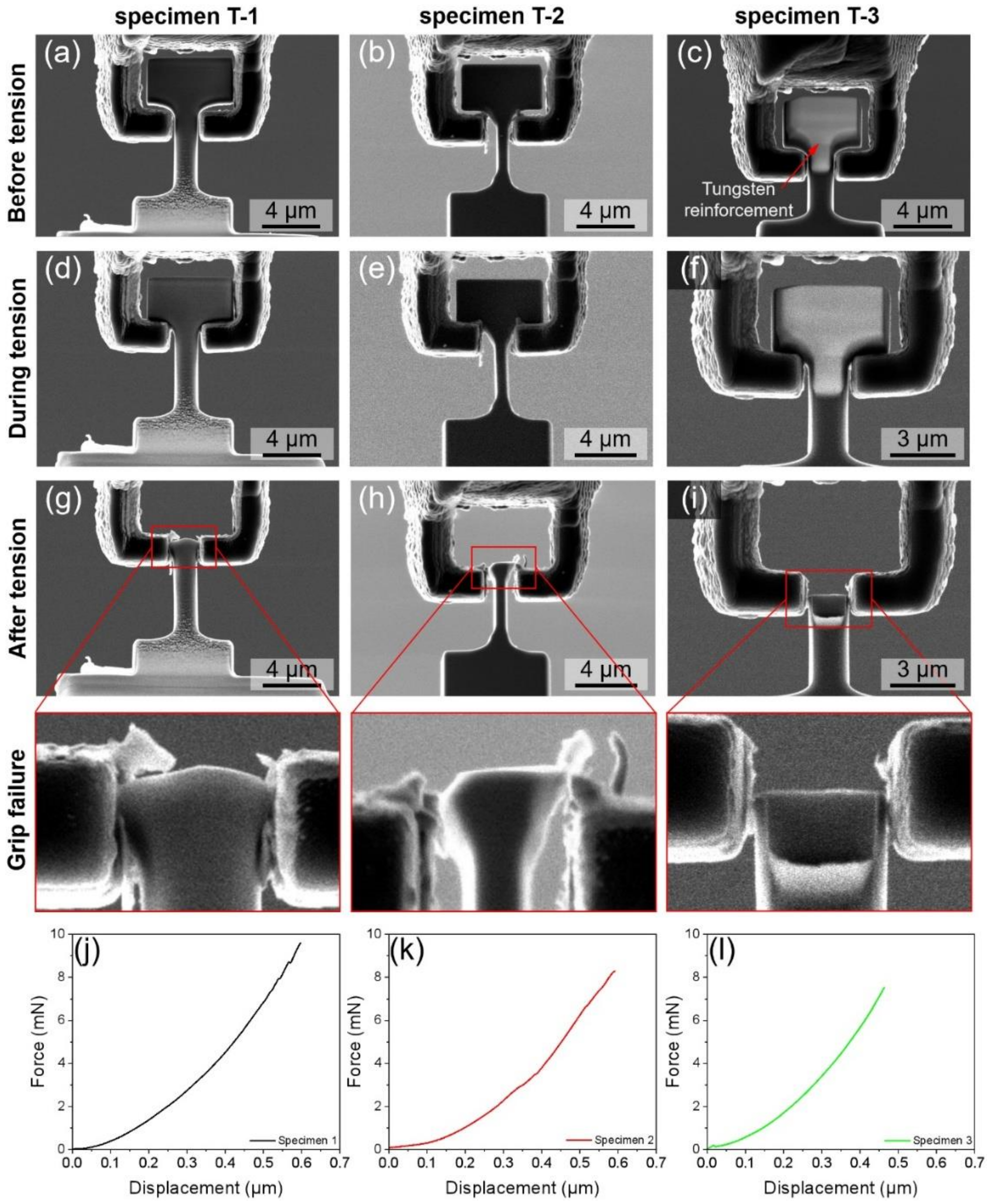

Supplementary Figure 14: **Grip-edge–controlled failure in micrometer-scale TiB_2 tensile tests and attempted mitigation strategies**. SEM images of the three tensile specimens (T-1 to T-3) in (a-c) the initial state prior to loading, (d-f) during tension, and (g-i) after fracture. In all three tests, fracture initiates reproducibly at the grip edge rather than within the calibrated gauge section, as evidenced by the post-mortem fracture morphologies. (j-l) The corresponding experimental load-displacement curves. To mitigate grip-edge failure, we explored two approaches: narrowing the gauge width to delay the onset of a critical grip-edge stress concentration (specimen T-2), and reinforcing the gripped region by depositing a protective tungsten layer over the grip section (specimen T-3). Neither strategy eliminated the dominant grip-edge stress concentration, and all specimens still failed at the grip edge.

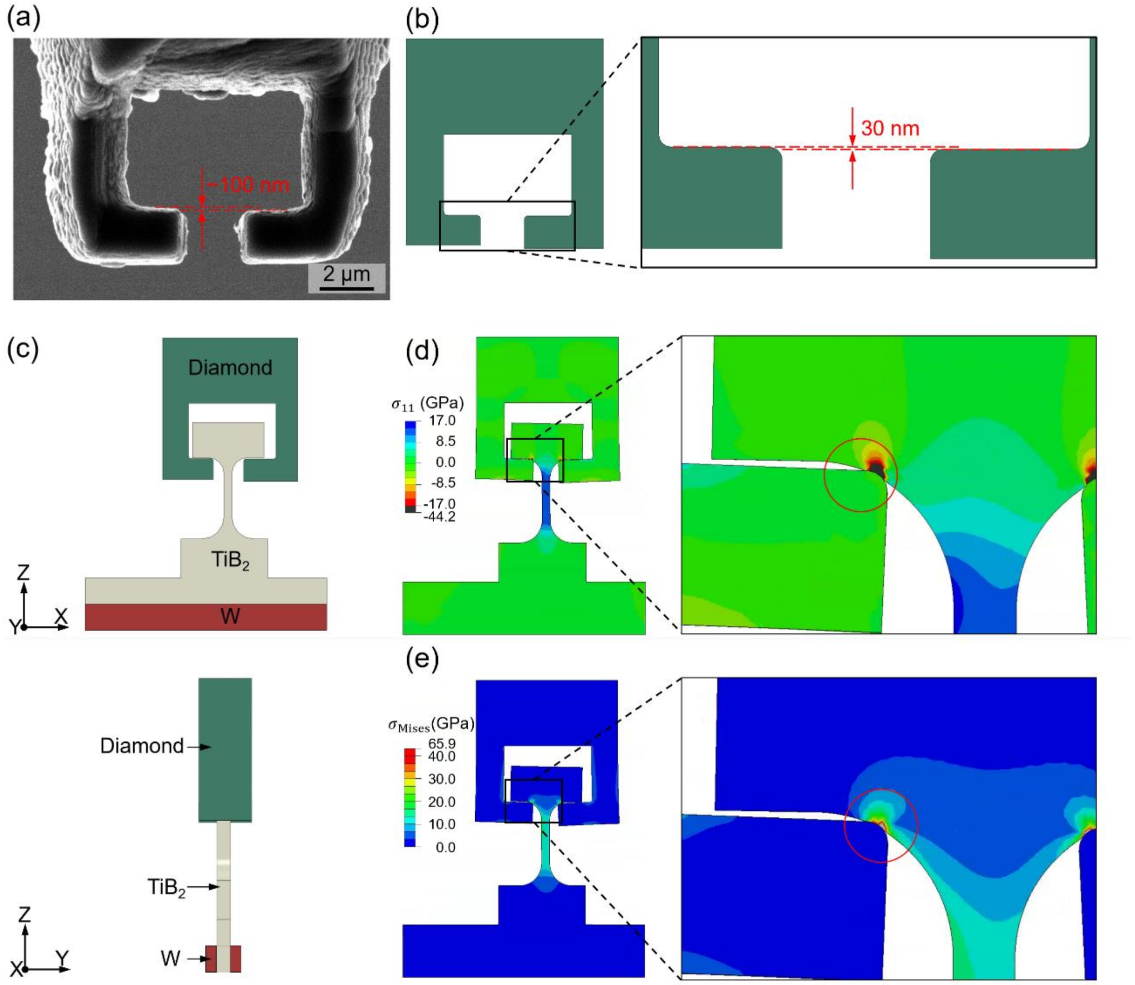

Supplementary Figure 15: FEM verification of grip-induced stress concentration in the micrometer-scale tensile specimen. (a) SEM image showing a slight misalignment between the two diamond gripping faces, on the order of ~100 nm. (b) Finite-element model of the diamond gripper incorporating a conservative misalignment of 30 nm. (c) One-to-one finite-element model of tensile specimen T-2 used for the in-situ tensile test. (d,e) Finite-element simulations of T-2 immediately prior to fracture, showing (d) axial normal stress (tension/compression) and (e) von Mises stress distributions, highlighting the severe stress concentration at the grip edge.

Because brittle single-crystal TiB_2 cannot tolerate such localized compressive stress concentrations of this magnitude, fracture invariably initiates at the grip before the gauge

reaches its intrinsic tensile limit. **We therefore report these microscale tensile experiments as a clear demonstration of a current experimental limitation (grip-controlled failure)**, not as a direct measurement of intrinsic microscale tensile strength. Consistent with this limitation, prior in-situ tensile tests on brittle single crystals have, to our knowledge, been demonstrated only at the nanoscale[1, 2, 3], and successful microscale implementations have not yet been reported.

Importantly, prior to grip-edge failure, **the gauge section in each specimen sustains a nearly uniform, multi-gigapascal tensile stress (up to ~13 GPa) over a micrometer-scale volume (> 4 μm^3) without fracture** (Fig. 6). In other words, even at this large stressed volume, the gauge remains exceptionally strong and intact. These tensile tests therefore indicate that **the gauge region contains no strength-limiting defects at the micrometer scale**, further supporting the effectiveness of our in-situ synthesis route for producing large TiB_2 particles with minimal flaw populations. It is therefore reasonable to expect that, if the gripping-induced stress concentration could be eliminated, the gauge section could be loaded to substantially higher tensile stresses, potentially enabling a direct measurement of the intrinsic microscale tensile limit. In this sense, **the present microscale tensile tests serve as a stringent "screening" step: they rule out gauge-defect-controlled failure and confirm that the observed limitation is imposed by the gripper rather than by the TiB_2 gauge itself**.

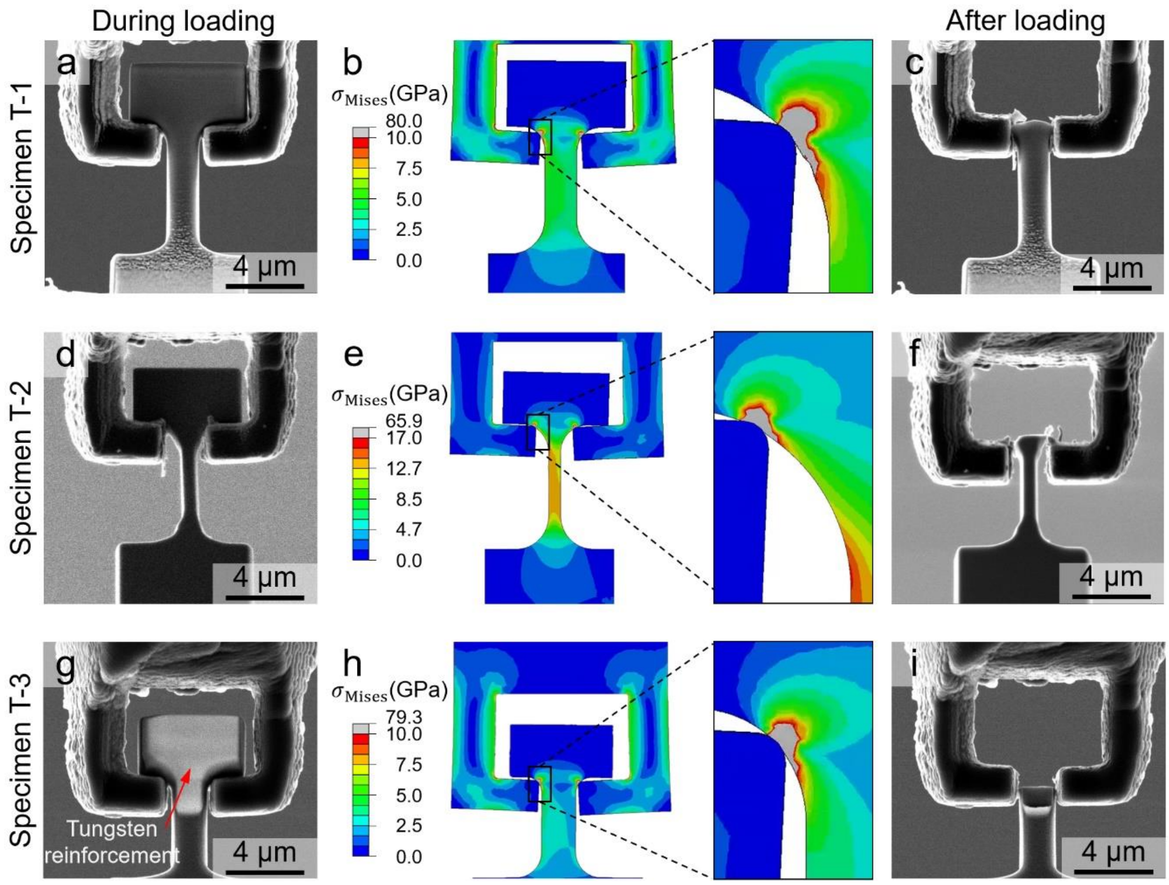

Figure 6. Microscale uniaxial tensile tests on TiB_2 tensile specimens and corresponding FEM analyses. (a-c) Specimen T-1, (d-f) specimen T-2, and (g-i) specimen T-3. Each row corresponds to one specimen. (a, d, g) In-situ SEM images at the final stable loading increment immediately prior to fracture at the grip edge. (b, e, h) Corresponding specimen-specific FEM maps of von Mises stress at the same applied displacement. (c, f, i) Post-mortem SEM images after failure. In all three tests, FEM reveals a strong stress concentration at the clamp edge, consistent with the experimentally observed fracture location, while the gauge section remains crack-free up to the onset of clamp-edge failure.

(2) Microscale four-point bending:

We further performed microscale four-point bending by preparing five FIB-machined TiB_2 beams (Supplementary Figs. 8-9). The beam geometry is schematically illustrated in Supplementary Fig. 7 and summarized in Supplementary Table 4. Here, we adopted a **clamped-clamped design**, in which both beam ends remain anchored to the larger TiB_2 body

that is still embedded in the surrounding matrix. This configuration ensures that the deformation during loading is **dominated by elastic bending of the TiB_2 beam**, while avoiding any contribution from matrix plasticity that would otherwise complicate and bias the FEM-based stress analysis.

To approximate four-point bending, we used a flat-punch indenter to generate a near-constant bending-moment region between the contact edges, thereby producing a more uniform tensile-stress field over a finite gauge length. Finite-element simulations (Supplementary Fig. 10) confirm that the stress distribution generated by the flat-punch configuration closely matches that of an idealized two-line-contact (two-point) four-point-bending setup (which is rarely available in micromechanics experiments), indicating that **our experimental configuration provides a reliable approximation to four-point bending** for stress analysis.

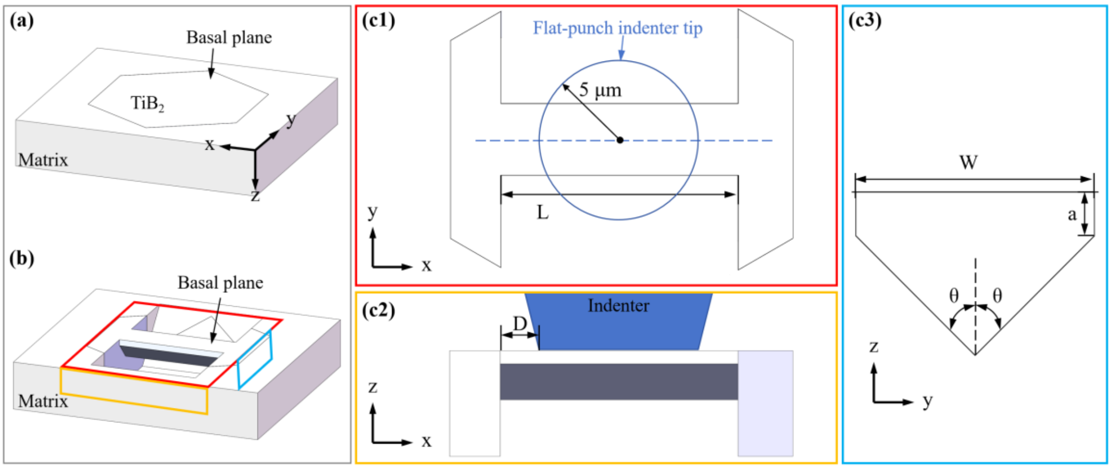

Supplementary Figure 7: Schematic of single-crystal TiB_2 particles partially embedded in a steel matrix and the resulting four-point bending beams (a) before and (b) after milling. By design, the top surface of each micro-cantilever is not exposed to FIB and remains unaffected. (c1–c3) Top, front, and side views of the four-point bending beam in (b).

Supplementary Table 4: Dimensions of FIB-milled four-point bending beams, listing geometric parameters L to D (as defined in Figure R5) and the distance D from the edge of the indenter to the one end of the beam.

ID	L (μm)	W (μm)	a (μm)	θ (°)	D (μm)	Preparation method
B-11	19.95	3.17	0.00	50	5.30	Polishing (HMS-2)
B-12	19.98	3.87	0.05	59	4.65	Polishing (HMS-2)
B-13	21.25	4.00	0.00	60	6.60	Polishing (HMS-2)
B-14	21.4	2.36	0.00	49	5.45	Polishing (HMS-2)
B-15	21.1	4.60	0.00	60	5.45	Polishing (HMS-2)

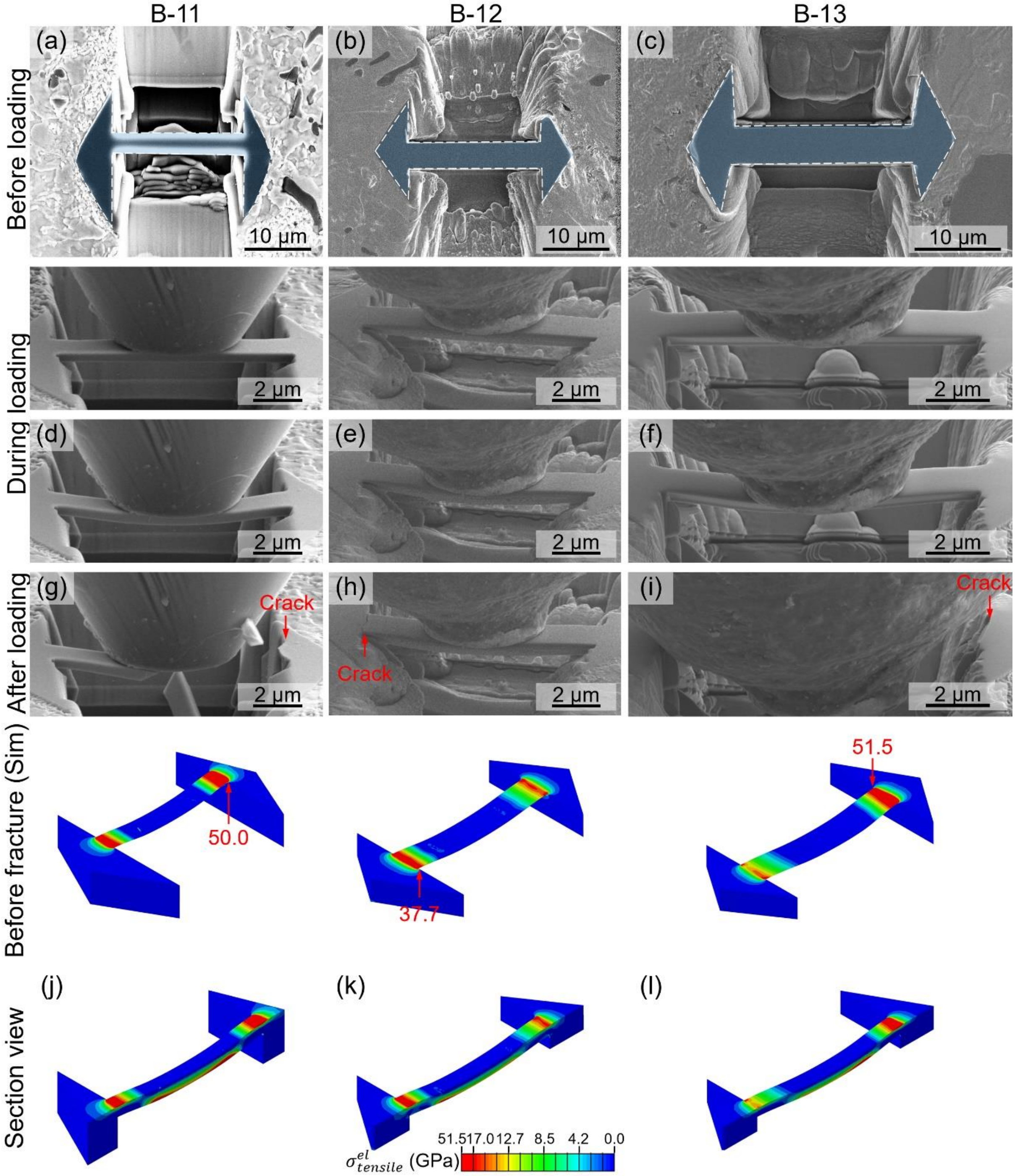

Supplementary Figure 8. Fixed-end four-point bending of basal-plane-oriented TiB_2 beams (B-11 to B-13) and corresponding FEM stress fields. (a-c) SEM images of specimens B-11, B-12, and B-13 in the initial state prior to loading. In all three beams, the basal planes are oriented nearly parallel to the matrix surface. (d-f) In-situ SEM images acquired at the final stable loading increment immediately prior to failure, showing the deformed configurations under four-point-bending-type loading with both ends effectively fixed. (g-i) Post-mortem SEM images after fracture, revealing the fracture surfaces. (j-l) Specimen-specific finite-element simulations reproducing the measured deformation profiles and showing the distribution of tensile (principal) stress on the tensile surface immediately prior to failure. Owing to the fixed-end boundary condition, up to three competing tensile-stress maxima can develop within a single beam: two near the fixed ends and one within the nominal constant-moment region. In each case, the experimentally observed fracture origin coincides with the FEM-predicted highest-stress location.

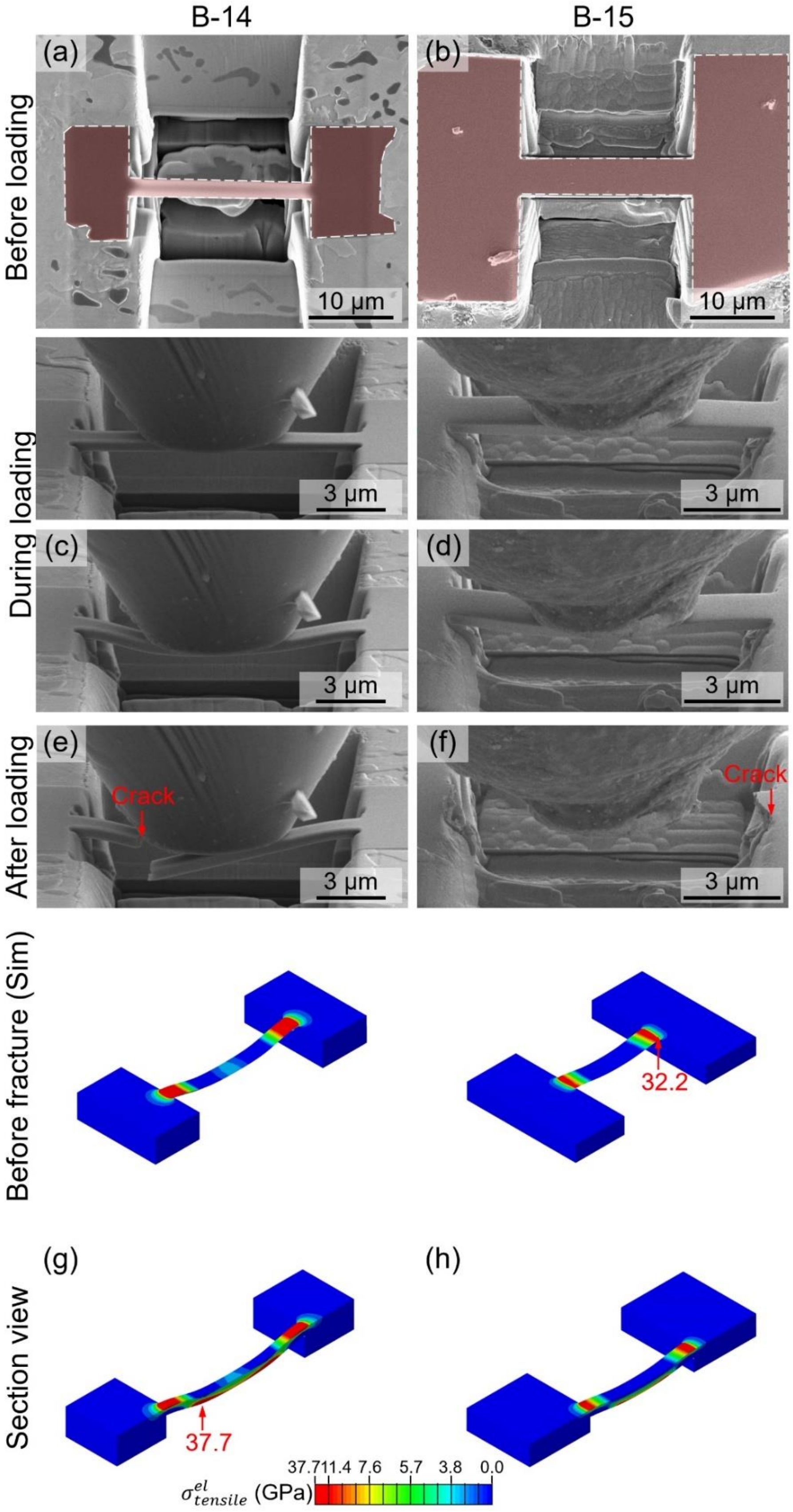

Supplementary Figure 9. Fixed-end four-point bending of prismatic-plane-oriented TiB_2 beams (B-14 and B-15) and corresponding FEM stress fields. (a-b) SEM images of

specimens B-14 and B-15 in the initial state prior to loading. In both beams, the prismatic planes are oriented nearly parallel to the matrix surface. (c-d) In-situ SEM images acquired at the final stable loading increment immediately prior to failure, showing the deformed configurations under four-point-bending-type loading with both ends effectively fixed. (e-f) Post-mortem SEM images after fracture, revealing the fracture surfaces. (g-h) Specimen-specific finite-element simulations reproducing the measured deformation profiles and showing the distribution of tensile (principal) stress on the tensile surface immediately prior to failure. Owing to the fixed-end boundary condition, up to three competing tensile-stress maxima can develop within a single beam: two near the fixed ends and one within the nominal constant-moment region. In each case, the experimentally observed fracture origin coincides with the FEM-predicted highest-stress location.

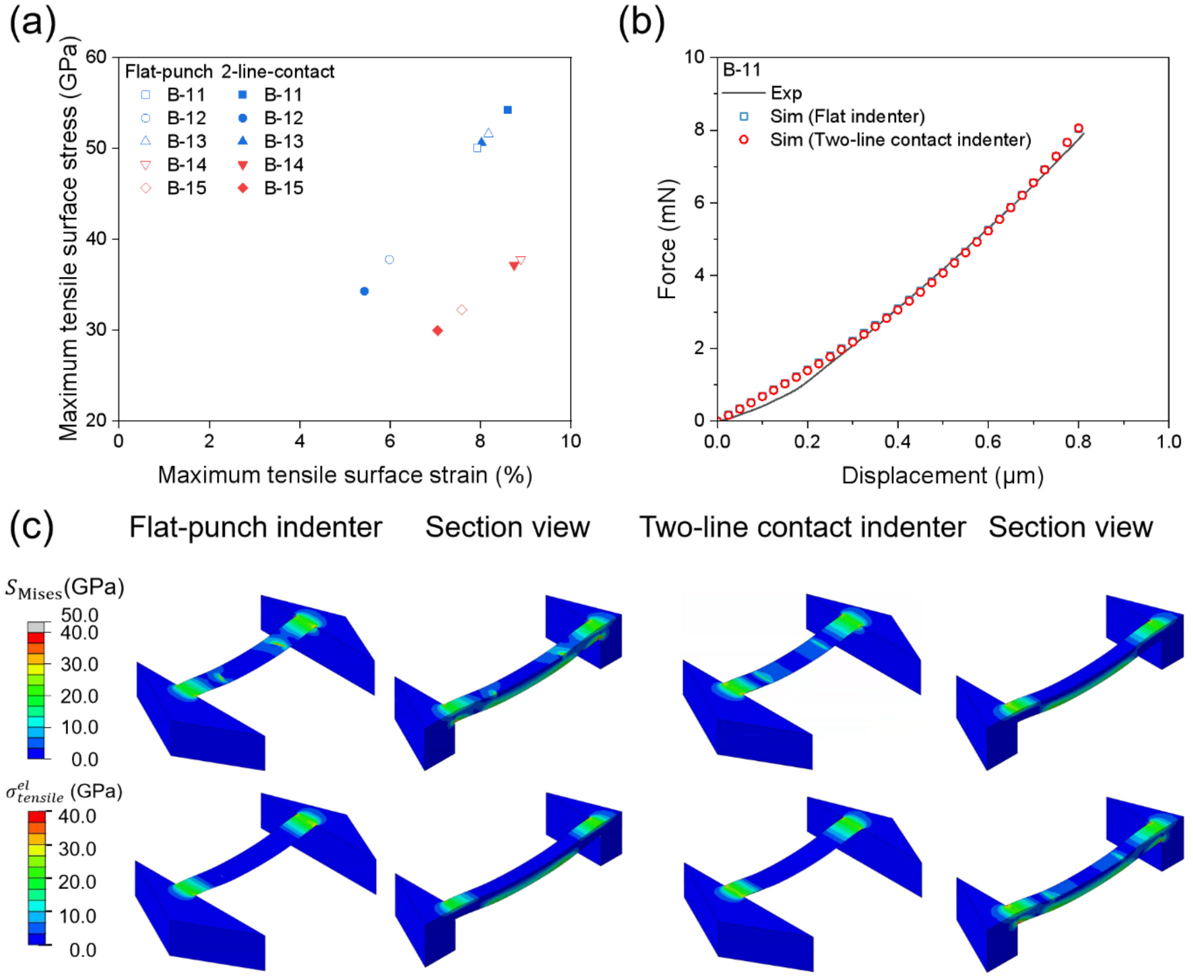

Supplementary Figure 10: FEM validation of the flat-punch approximation for microscale four-point-bending-type loading. Finite-element simulations confirm that the

stress field generated by the flat-punch loading configuration closely matches that of an idealized two-line-contact indenter with the same contact spacing (equal to the flat-punch diameter). (a) Comparison of the maximum tensile principal surface stress versus maximum tensile principal surface strain for specimens B-11 to B-15 extracted from simulations using the flat-punch and two-line-contact indenters, showing similar $\sigma_{\mathrm{I,max}}$ and $\varepsilon_{\mathrm{I,max}}$ values. (b) Simulated load-displacement curves for both indenter types compared with the experimental response, demonstrating similarly good agreement. (c) Representative stress fields (von Mises stress and tensile principal stress) immediately prior to failure for both indenter types, revealing nearly identical distributions and the same three competing tensile-stress hot spots (two near the clamped ends and one within the nominal constant-moment region).

As shown in Supplementary Figs. 8-9, multiple spatially separated tensile-stress hot spots develop within a single specimen, creating several competing sites where a critical defect (if present) could trigger fracture. These hot spots include two at the upper-surface beam edges near the clamped ends and one in the lower, mid-span region. This multi-peak stress field arises from the clamped-clamped boundary condition, which introduces end constraints and elevates tensile stresses near the fixed ends. Four of the five beams (B-11, B-12, B-13, B-15) exhibit their globally maximum tensile stress at the upper-surface beam edges near the clamped ends. In all these five cases, fracture initiated **exactly at these FE-predicted maximum-stress locations**. The remaining specimen (B-14) shows the maximum tensile stress located on the l**ower surface** within the constant-moment region, and the experiment again confirmed fracture initiating at that same lower-surface location.

This **one-to-one correspondence** between the FE-predicted tensile-stress maxima and the experimentally observed fracture origins indicates that **failure is governed by the local maximum tensile stress, rather than by randomly distributed critical flaws** that would be expected to trigger fracture at locations not associated with the computed stress peaks. Therefore, although our microscale four-point bending does not produce a perfectly uniform large-volume tensile-stress field in every specimen, it serves as **strong independent evidence** supporting the central conclusion of this work: in-situ grown micrometer-scale TiB_2 exhibits

an exceptionally low population of strength-limiting defects and can sustain very high tensile stresses up to the local fracture limit.

Consistent with this stress-controlled fracture behavior, the maximum tensile surface stresses extracted from cantilever and four-point-bending geometries are highly consistent, as shown in revised Fig. 3.

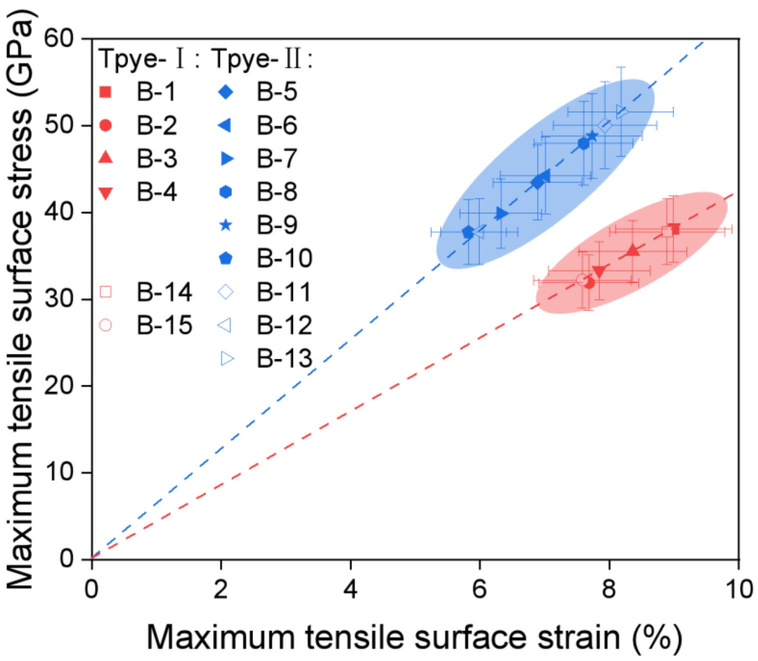

Figure 3. Plot of maximum tensile surface stress at failure versus corresponding maximum tensile surface strain for Type-I and Type-II micro-cantilever and four-point bending beams, revealing only moderate anisotropy. Each symbol corresponds to one test. $\sigma_{\mathrm{I,max}}$ and $\varepsilon_{\mathrm{I,max}}$ are the maximum tensile surface stress and strain at the failure point, extracted from specimen-specific FEM calibrated by the measured load-displacement curve. Error bars represent the estimated specimen-specific analysis/measurement uncertainty (±8%), obtained by propagating the sensitivities to (i) matrix-support compliance, (ii) indenter-specimen friction, and (iii) SEM dimensional measurement uncertainty (Supplementary Note S3; Supplementary Figs. 18-20).

(3) Quantitative stressed-volume analysis of micro-cantilever beams:

Finally, we re-examined our original cantilever-bending geometry to quantify the stressed volume associated with the high tensile surface stresses. Rather than relying on qualitative statements about a “small stressed region,” we now make the stress-volume issue explicit by reporting the stress-volume distribution (histogram) for the representative beams B-9 shown in Fig. 2.

Using the FEM stress fields, we compute the specimen volume exceeding a given tensile-stress threshold. The resulting stress-volume histogram shows that the material volume experiencing very high tensile stress (e.g., > 15 GPa) already **exceeds 5 μm³** in this beam (Supplementary Fig. 5). This analysis demonstrates that, even in cantilever bending where the stress field is non-uniform, a micrometer-scale TiB_2 volume can sustain very high tensile stresses. The result is consistent with an exceptionally low density of critical, strength-limiting flaws in the in-situ grown TiB_2.

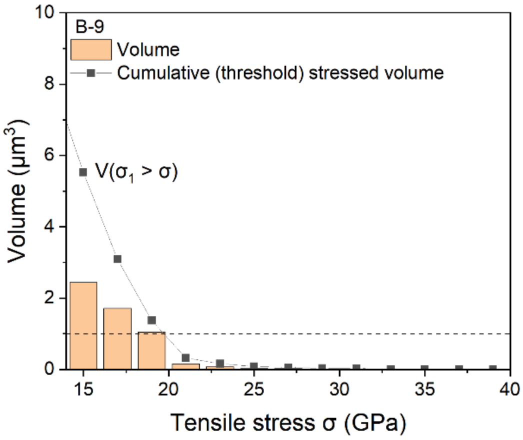

Supplementary Figure 5: Stress-volume distributions quantify micrometer-scale high-tensile-stress volumes in cantilever bending. Using specimen-specific FEM fields for the representative beam (B-9), we compute the cumulative stressed volume $V(\sigma_I > 15$ GPa$)$ and the binned volume histogram $h(\sigma_I)$, where σ_I is the maximum principal tensile stress. The analysis shows that the TiB_2 volume subjected to very high tensile stress is already micrometer-scale (e.g., $V(\sigma_I > 15$ GPa$) > 1$ μm^3), despite the non-uniform bending stress field.

Summary:

With these additions, the revised manuscript no longer relies on micro-cantilever bending alone. Instead, the central conclusion of "micrometer-scale TiB_2 produced by our route is effectively free of strength-limiting defects" is supported by three independent and mutually consistent lines of evidence:

1) **Cross-geometry, multi-specimen validation of a stress-controlled failure criterion**. Across **twenty specimens** spanning four distinct test geometries (cantilever, C-shaped beams, microscale tension, and microscale four-point bending), fracture consistently initiates at the FE-predicted **maximum tensile-stress locations**, demonstrating that failure is governed by the local tensile stress field rather than by randomly distributed critical defects.

2) **Direct microscale tensile loading establishes a conservative lower bound on intrinsic tensile strength in a large gauge volume**. In tensile tests, the large gauge volume (> 4 μm^3) sustains up to ~13 GPa of nearly uniform tensile stress prior to the grip-edge failure, providing strong evidence that critical defects are absent within a micrometer-scale volume under high tensile loading.

3) **Quantified high-stress volume in cantilever bending demonstrates micrometer-scale stressed volumes**. For the cantilever beam B-9, a substantially larger material volume experiences very high tensile stresses; fracture origins again coincide with FE-predicted tensile-stress maxima. Moreover, we now explicitly quantify the stress-volume distribution, directly addressing concerns about stress localization and demonstrating that the volume subjected to >15 GPa tensile stress can exceed 5 μm^3.

Accordingly, we believe the reviewer's central concern, that the exceptionally high values might arise simply because bending probes only a very small stressed volume and therefore could "miss" defects, has now been addressed directly and quantitatively through the added micrometer-scale tensile and four-point-bending experiments, together with the explicit stressed-volume analysis of the cantilever beam. **We would be grateful for any further**

suggestions the reviewer may have, and we are fully willing to refine the manuscript further to ensure that the conclusions are presented as rigorously and convincingly as possible.

Revisions in Main Text and Supplementary Information:

Finally, we thoroughly revised the manuscript to integrate all newly added experiments and results while preserving a clear and logical narrative. The key revisions are summarized as follows:

1. In the revised main text, we added new sentences in the Abstract to briefly describe the additional experiments and highlight their key results.

…In-situ micromechanical testing of twenty specimens with distinct geometries—including cantilevers, four-point bending beams, and C-shaped structures—combined with specimen-specific finite element analysis, consistently reveal strengths in the tens of gigapascals, approaching the near-theoretical limit for this ceramic. Control experiments show that these ultra-high strengths are robust across matrix chemistries and surface preparation, and orientation-dependent measurements indicate only modest anisotropy. Microscale tensile experiments further show that a large gauge volume (~4 μm^3) can sustain very high, nearly uniform tensile stress of ~13 GPa without fracture, indicating an exceptionally low population of strength-limiting defects within a micrometer-scale stressed volume…

2. In the revised main text, we added new sentences in the Introduction to clarify the “micrometer-scale stressed-volume” motivation and to note the added independent tensile benchmark.

(Page 3, line 3)

In this study, we report the first experimental evidence of near-theoretical strength in

micrometer-sized ceramics, using single-crystalline TiB_2 formed via eutectic reaction in steel as a model system. This easily synthesized ceramic phase achieves maximum tensile surface stresses at failure exceeding 30 GPa and tensile elastic limits up to 9.0% in large specimens with micrometer-scale cross-sections under bending, distinct from the commonly reported micron-long nanoneedles, whose cross-sections (and thus stressed volumes) remain nanoscale. While comparable tensile strains in cross-section-wise microscale specimens were previously attained only in defect-minimized silicon via advanced wafer technologies (also probed by bending), TiB_2 combines such strains with markedly higher strength due to its superior elastic modulus. Using in-situ micromechanical testing of crystallographically oriented cantilevers, fixed-end beams, and C-shaped specimens with specimen-specific finite-element analyses, we quantify the intrinsic anisotropic elasticity of TiB_2 and its tensile-surface failure stress, which approaches near-theoretical limits. In addition, microscale uniaxial tensile tests on TiB_2 specimens show that the gauge sections can sustain up to ~13 GPa of near-uniform tensile stress without detectable cracking. Although final failure occurs at the gripping edge due to unavoidable stress concentration, these tests provide an independent tensile benchmark and support the conclusion that the in-situ grown TiB_2 is effectively free of strength-limiting defects at the micrometer scale.

3. We added a new opening paragraph in the Results and discussion to clearly show the rationale and logic for testing the four geometries (cantilever, four-point bending, C-shape, and tension), so readers can understand why the additional experiments were performed and how each geometry provides complementary evidence.

(Page 3, line 29)

…Because approaching near-theoretical strength at the micrometer scale critically depends on suppressing strength-limiting defects, we designed a hierarchy of test geometries that progressively varies the stress state and specimen volume for robust failure analysis. We first use crystallographically oriented micro-cantilever bending to quantify the intrinsic anisotropic elasticity and tensile-side fracture stress of TiB_2, with specimen-specific FEM

mapping the maximum surface tensile stress/strain at failure. The cantilever results suggest that the ultrahigh stresses attained are consistent with an exceptionally low population of critical defects in in-situ-grown TiB_2. We then test this inference using two complementary "defect-sensitivity-probing" geometries. A microscale four-point-bending-type configuration generates multiple spatially separated tensile-stress hot spots within a single beam, creating several competing sites where a randomly located critical flaw could trigger premature failure. Additionally, C-shaped structures provide an independent loading geometry, serving as an internal consistency check for the anisotropic elastic parameters extracted from the beams and also enabling cyclic loading-unloading with unobstructed views to probe whether damage accumulates from subcritical flaws. Finally, to probe larger, more uniformly stressed volumes under nominal uniaxial loading, we perform direct tensile tests, with FEM quantifying unavoidable grip-edge stress concentrations, and further assess internal flaw populations by serial FIB sectioning of large TiB_2 particles. Together, these experiments establish a consistent picture in which TiB_2 failure is governed by the local tensile-stress maximum rather than by a weakest-link distribution of pre-existing critical defects.

4. We added a new paragraph in the Results and discussion that explicitly quantifies the stressed volume for beam B-9 to obtain a stress-volume distribution.

(Page 8, line 18)

Finally, to make the stressed-volume issue explicit, we post-processed the specimen-specific FEM fields to obtain the stress-volume distribution for this beam (B-9), showing that the TiB_2 volume experiencing very high tensile stress (e.g., > 15 GPa) is already micrometer-scale (Supplementary Note S2 and Supplementary Fig. 5). This highlights the importance of increasing the specimen cross-section to ensure that a substantial material volume is subjected to high tensile stress.

5. We added two new paragraphs in the Results and discussion describing the additional fixed-end microscale four-point-bending experiments (multiple competing tensile-stress hot

spots within one specimen) and the micrometer-scale tensile tests (nearly uniform gauge stress prior to grip-edge failure), respectively.

(Page 10, line 1)

The observation that micrometer-scale TiB_2 beams reproducibly reach ultrahigh $\sigma_{I,max}$ implies that strength-limiting defects are exceedingly scarce within the TiB_2 particles. If failure were dominated by a conventional weakest-link population of randomly distributed critical flaws (pores/microcracks), one would expect crack initiation to occur at other locations different from the ones with stress maxima, together with markedly larger specimen-to-specimen scatter even for similar orientations and sizes. To test this inference more stringently, we designed a microscale four-point bending configuration (Fig. 4a, Supplementary Fig. 7) that generates multiple spatially separated tensile-stress hot spots within a single specimen, thereby providing several competing sites where a critical defect, if present, could trigger failure. Specifically, we fabricated five clamped-clamped TiB_2 beams (Supplementary Figs. 8-9) with dimensions summarized in Supplementary Table 4 and loaded them using a flat punch to approximate the constant-moment region of four-point bending (Fig. 4b). Finite-element analysis (Supplementary Fig. 10) confirms that the stress field produced by the flat-punch approach closely matches that of an idealized two-line-contact configuration, which is rarely available in micromechanics experiments. Further specimen-specific simulations (Fig. 4d, Supplementary Figs. 8-9), whose accuracy is validated by the excellent agreement between experimental and simulated load-displacement curves (Fig. 4e), show that the resulting boundary condition produces up to three competing tensile-stress maxima (two near the fixed ends and one within the nominal constant-moment region), so that defect-controlled fracture could, in principle, initiate at any of these locations. Experimentally, however, all five beams fractured exactly at the FEM-predicted highest-stress location for that specimen (Fig. 4c, Supplementary Figs. 8-9). In five beams, $\sigma_{I,max}$ occurred at the upper-surface edges near the fixed ends due to end constraints, and fracture initiated precisely at those edges; the remaining beam exhibited the expected constant-moment stress state with the maximum tensile surface stress located on the lower surface, and fracture

initiated accordingly. This one-to-one correspondence between predicted $\sigma_{I,max}$ locations and observed fracture origins across distinct hot spots indicates that fracture is stress-controlled rather than defect-controlled, consistent with an exceptionally low population of strength-limiting defects in the in-situ-grown TiB_2.

(Page 13, line 11)

Finally, we attempted direct micrometer-scale tensile testing to probe a larger, nearly uniformly stressed gauge volume under nominal uniaxial loading. Three micrometer-sized TiB_2 tensile specimens (T-1 to T-3) were FIB-fabricated and pulled using PFIB-machined diamond micro-grippers (Supplementary Fig. 13). In all three tests, fracture occurred reproducibly at the grip edge rather than within the calibrated gauge section (Fig. 6, Supplementary Fig. 14), indicating that failure was governed by unavoidable stress concentration introduced by the gripping condition. This conclusion is supported by both the SEM fracture morphologies and the similar maximum loads sustained by the specimens despite their different cross-sectional areas (Supplementary Fig. 14). FEM analysis of the specimen-gripper assembly reveals a severe grip-edge stress concentration: for specimen T-2, the peak Von Mises stress at the grip edge reaches ~65.9 GPa immediately prior to fracture, while the gauge region sustains a nearly uniform tensile stress of ~13 GPa (Fig. 6, Supplementary Fig. 15). Because the maximum load is similar across specimens, the uniform gauge stress decreases with increasing cross-sectional area. This dominant stress concentration could not be eliminated by either narrowing the gauge width (T-2) or reinforcing the gripped region via deposition of a protective tungsten layer over the grip section (T-3). Although this grip-controlled failure prevents a direct measurement of the intrinsic micrometer-scale tensile fracture strength, the gauge section (> 4 μm^3 in volume) nonetheless survives up to ~13 GPa of near-uniform tensile stress without cracking, providing a conservative lower-bound tensile benchmark and further supporting the scarcity of strength-limiting defects in our TiB_2 at the micrometer scale. To directly examine whether large TiB_2 particles contain hidden internal flaws, we serially sectioned three large TiB_2

particles by FIB and imaged each newly exposed cross-section by SEM. Across multiple slices along the [0001] direction, the TiB_2 interior remains fully dense and exhibits uniform contrast, with no pores, microcracks, or discontinuities that persist from slice to slice (Supplementary Fig. 16a-c). This provides direct microstructural evidence that the measured ultrahigh strengths are governed by the applied local tensile-stress maximum rather than pre-existing internal flaw populations.

6. In the paragraph describing the C-shaped specimen, we added a sentence clarifying its complementary role relative to the cantilever and fixed-end four-point bending tests, thereby smoothing the logic and strengthening the overall comparison.

(Page 11, line 16)

…Beyond providing a distinct stress state, this configuration offers an internal consistency check for the anisotropic elastic parameters extracted from the cantilever beams and, importantly, enables cyclic loading-unloading with an unobstructed view of the highly stressed region, thereby allowing us to probe whether any subcritical flaws accumulate damage under repeated high-stress exposure…

7. We have replaced the original terminology of "tensile fracture strength", which may be misinterpreted as nominal tensile strength obtained by tensile tests, by the more accurate phrase "maximum tensile (principal) surface stress at failure" at first use, and we explicitly define it as $\sigma_{\mathrm{I,max}}$. We also explicitly state that $\sigma_{\mathrm{I,max}}$ should not be interpreted as a nominal tensile stress averaged over the full cross-section of a uniaxial tensile specimen. We have made the stress definitions unambiguous throughout the main text, figure captions, and axis labels.

8. In the Methods, we extensively expanded the section "Fabrication of micromechanical TiB_2 specimens" to explicitly state that four complementary geometries were employed

(cantilevers, fixed-end four-point-bending beams, C-shaped structures, and tensile specimens) and to direct readers to the corresponding schematics and dimensions in the Supplementary Information.

(Page 18, line 25)

…TiB_2 is generated during casting by eutectic/near-eutectic solidification in the Fe–Ti–B system. During cooling, Ti and B exhibit limited solubility in Fe and partition to form Ti-B-enriched liquid regions; TiB_2 precipitates from these regions as primary faceted crystals. As solidification proceeds, the remaining Fe-rich liquid transforms into the steel matrix while preserving the already-formed TiB_2 (Supplementary Note S1), yielding the partially embedded hexagonal-prismatic TiB_2 morphology observed after deep etching (Supplementary Fig. 1). Sample surfaces were prepared by sequential grinding (80-3000 grit), polishing with diamond suspensions, and final vibratory polishing with a 50-nm SiO_2 colloidal suspension. TiB_2 was then exposed by deep-etching in 33 vol% HCl.

To effectively vary the stress state and specimen volume for robust failure analysis, we employed four complementary specimen geometries, including (i) micro-cantilever beams (B-1 to B-10), (ii) clamped-clamped (fixed-end) microscale four-point-bending beams (B-11 to B-15), (iii) C-shaped structures (C-1 and C-2), and (iv) microscale tensile specimens (T-1 to T-3). Focused ion beam (FIB) milling was performed using a Ga+ ion source at 30 kV in a Helios 5UC SEM/FIB system. The region of interest was first isolated with an ion beam current of 2-5 nA. Rough milling was conducted at 300-700 pA, followed by fine milling at 100 pA to smooth surfaces and minimize ion-induced damage. Schematics and geometric parameters for the cantilevers, four-point-bending beams, C-shaped structures, and tensile specimens are provided in Supplementary Figs. 2, 7, 11, and 13, with dimensions summarized in Supplementary Tables 1-2 and 4-7. For the four-point-bending beams and cantilevers, the top tensile surface was intentionally preserved from direct FIB exposure (Supplementary Figs. 2 and 7) to minimize ion-induced surface flaws in the most highly stressed region.

9. In the Methods, we expanded the section "In-situ micro-mechanical testing" to explicitly describe the testing procedures for all the four geometries (cantilever, fixed-end four-point bending, C-shaped compression, and tensile tests).

(Page 19, line 23)

Mechanical testing was performed on a micro/nano-mechanical platform (FT-NMT04, FemtoTools) equipped with flat-punch and cube-corner indenter tips. Unless otherwise stated, tests were conducted under displacement control at 5 nm/s, with load and displacement recorded at a sampling rate of 3 kHz. Real-time imaging was used to track indenter position and specimen response, enabling correlation of mechanical data with the onset and location of cracking/fracture.

For bending tests (cantilevers and fixed-end four-point-bending beams), loading was applied normal to the specimen surface. Beams B-1 to B-6 were loaded with a flat punch to maintain full-face contact. For micro-cantilever beams B-7 to B-10 machined on smooth surfaces, a cube-corner indenter was used to precisely define the loading distance (Supplementary Fig. 2d2), eliminating the edge-position ambiguity of a flat punch. For fixed-end beams B-11 to B-15, flat-punch loading was used to approximate a constant-moment region in the four-point-bending geometry. For C-shaped structures, flat-punch loading enabled monotonic loading to fracture (Supplementary Fig. 12) and cyclic loading-unloading with unobstructed views to probe damage accumulation under repeated high-stress exposure. For microscale tensile tests, TiB_2 tensile specimens were pulled using a PFIB-machined diamond gripper design (Supplementary Fig. 13). SEM images of the tensile specimens and representative fracture morphologies are provided in Supplementary Fig. 14.

10. In the Methods, we also expanded the section "FEM analyses" to (i) define unambiguously the reported fracture strength in bending as the FE-resolved maximum principal tensile surface stress at failure (rather than a nominal uniaxial tensile stress) and (ii) describe the stress-volume post-processing used to quantify the high-stress volume in

bending.

(Page 20, line 15)

Finite element (FE) simulations were performed in Abaqus/CAE for each tested specimen geometry (cantilever, fixed-end four-point bending, C-shaped, and tension) using three-dimensional models discretized with 10-node quadratic tetrahedra elements (C3D10). Each model contained ~10^6 elements with mesh refinement in high-gradient regions (e.g., contact and fillet zones). Model dimensions were extracted from SEM measurements of each specimen. Single-crystal TiB_2 was modeled as a transversely isotropic, linearly elastic solid. The anisotropic elastic constants were determined by fitting simulated and experimental load-displacement responses of the cantilever beams and then applied without further adjustment to the other geometries (four-point bending and C-shaped), providing an internal consistency check. During FIB fabrication the TiB_2 particles retained sufficient supporting volume; consequently, elastic deformation of the surrounding matrix had a negligible influence on the beam's load-bearing stress-strain response (Supplementary Fig. 18). Indenter tips were modeled as rigid bodies, and surface-to-surface contact was used between the indenter and TiB_2. To capture potential friction effects in bending, surface-to-surface contact between the indenter tip and specimen was defined with Coulomb friction (f varied from 0.1 to 1). The extracted $\sigma_{\mathrm{I,max}}$ and $\varepsilon_{\mathrm{I,max}}$ were insensitive to f over this range (Supplementary Fig. 19).

To estimate the uncertainty shown as error bars in Fig. 3, we quantified the sensitivity of the FEM-extracted $\sigma_{\mathrm{I,max}}$ and $\varepsilon_{\mathrm{I,max}}$ to three dominant inputs: (i) compliance of the supporting matrix beneath the TiB_2 beam (rigid versus elastoplastic substrate), (ii) the indenter-specimen Coulomb friction coefficient (f = 0.1-1.0), and (iii) SEM-based dimensional measurement uncertainty of beam width and height (±30 nm edge-definition uncertainty). The resulting variations were ±1%, ±4%, and ±3%, respectively (Supplementary Figs. 18-20), which we conservatively summed to obtain an overall ±8% uncertainty per specimen (Fig. 3).

For bending-based tests (cantilevers and fixed-end four-point bending), the reported "fracture strength" corresponds to the maximum tensile (principal) surface stress at the failure point, defined as the peak value of the maximum principal tensile stress on the tensile surface at the final stable loading increment immediately prior to the first cracking event. This is a local, FE-resolved surface stress in bending and should not be interpreted as a nominal uniaxial tensile stress averaged over the full cross-section. The corresponding maximum tensile strain was extracted from the same location and loading increment. For microscale tensile specimens, FE simulations incorporating the measured grip alignment were used to quantify the stress distribution and to identify the nearly uniform tensile stress sustained in the gauge section immediately prior to grip-edge failure (Supplementary Fig. 14).

To explicitly quantify the stressed volume associated with high tensile stresses in bending, we post-processed FE stress fields to compute the cumulative TiB_2 volume exceeding a chosen tensile-stress threshold by integrating element volumes as a function of maximum principal tensile stress. This stress-volume analysis was performed for representative cantilever beams with the largest cross-sections to directly assess the micrometer-scale volume subjected to ultrahigh tensile stress.

11. In the revised Supplementary Information, we added Supplementary Note S2 and Supplementary Fig. S5 to explicitly quantify the stressed volume in cantilever bending via stress–volume distributions. We also added/updated the Supplementary figures/tables documenting the microscale four-point-bending geometry and FEM validation (Supplementary Figs. 7-10; Supplementary Table 4) and the microscale tensile specimen/gripper fabrication and FE stress analysis (Supplementary Fig. 13 and related tables/figures), providing full experimental and modeling details supporting the new validations included in the main text.

Comment 2: *Please provide microstructure characterization results from the TiB2 particles to prove that they are actually defect-free. I think FIB cross section of several particles will show us the defects like tiny crack or pores or their absence of in the* TiB_2 *particles.*

Response: Thank you for this constructive suggestion. To directly verify the internal integrity of the large TiB_2 particles, we performed serial FIB sectioning followed by cross-sectional SEM imaging, which provides a straightforward assessment of internal pores and microcracks, as suggested by the reviewer. The new results are included as Supplementary Fig. 16, where three randomly selected large TiB_2 particles are sectioned sequentially along the [0001] direction.

These observations provide direct evidence that the TiB_2 is essentially defect-free:

1) The interior of TiB_2 remains fully dense, with uniform contrast and no micro-cracks, pores, or internal flaws. The absence of any discontinuities persisting across slices strongly indicates that there are no internal flaw populations that could act as critical crack initiation sites within the sampled micrometer-scale volume.

2) The only minor irregularities we observe are confined to the near-surface region and do not extend into the interior. Based on their morphology, we attribute these shallow features to redeposition-related roughness, rather than intrinsic defects formed during eutectic growth. After near-complete TiB_2 formation, local depletion of Ti and B terminates growth. This termination can lead to slight surface non-uniformities (e.g., faceting/edge roughness) on the external surface, but importantly these features are not internal cracks or pores and do not compromise the dense single-crystal interior.

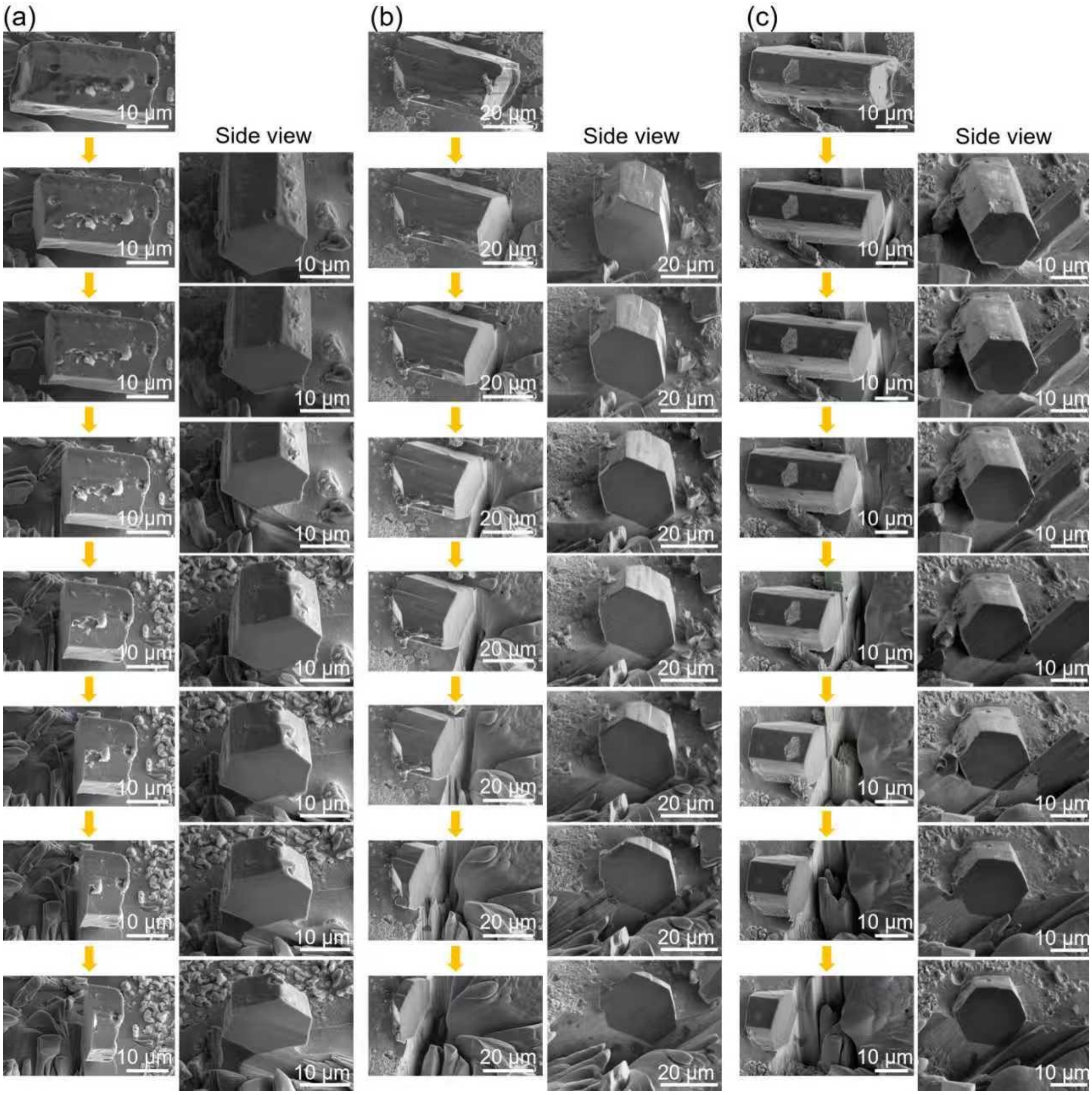

Supplementary Figure 16: Serial FIB cross-sectioning confirms a dense, defect-free interior in large TiB_2 particles. (a–c) Three representative large single-crystal TiB_2 particles were sequentially sectioned by FIB along the [0001] direction. For each particle, the left row shows the newly exposed surface after successive sectioning steps and the right row shows the corresponding side-view images of the cross-section. No internal pores or microcracks are observed throughout the sectioned volumes. Any small irregularities are limited to the near-surface region and are consistent with redeposition-related roughness/edge faceting rather than intrinsic defects formed during eutectic growth.

We appreciate the reviewer's suggestion, as it strengthens the manuscript by providing a direct microstructural confirmation of the defect-free nature of the large TiB_2 particles. Accordingly, we revised the main text by explicitly pointing readers to the new serial-sectioning evidence:

(Page 14, line 19)

…To directly examine whether large TiB_2 particles contain hidden internal flaws, we serially sectioned three representative large TiB_2 particles by FIB and imaged each newly exposed cross-section by SEM. Across multiple slices along the [0001] direction, the TiB_2 interior remains fully dense and exhibits uniform contrast, with no pores, microcracks, or discontinuities that persist from slice to slice (Supplementary Fig. 16a-c). This provides direct microstructural evidence that the measured ultrahigh strengths are governed by the applied local tensile-stress maximum rather than pre-existing internal flaw populations.

We also added one new subsection and its paragraph in the Methods entitled "Cross-sectional sectioning to assess internal flaws", describing the procedure and directing readers to the new figure:

(Page 22, line 2)

To evaluate whether large TiB_2 particles contain internal pores or microcracks that could act as strength-limiting defects, three representative large single-crystal TiB_2 particles were randomly selected and serially sectioned by FIB along the [0001] direction and imaged by SEM (Supplementary Fig. 16).

Comment 3: *I am not sure if it is correct to use cross-sectional area of a bend testing specimen to report the tensile fracture strain of that sample. Again, the tensile stresses are only concentrated at a small area on the top surface, and stresses are not equally distributed over the entire cross-section of the specimens reported in Figure 5. I am not sure about the data from the literature, but if they are from actual tensile tests, the comparison in figure 5 is*

not valid.

Response: Thank you for raising this very important point. We agree with the reviewer that our original Fig. 5 could be misleading because it combined data from **tensile tests** and **bending tests** in a single plot. In uniaxial tension, the reported cross-sectional area is the load-bearing area under (approximately) uniform axial stress, whereas in bending the stress is non-uniform across the section and the cross-sectional area serves only as a **geometric size descriptor**, not a stressed-area measure. Mixing these two types of datasets in one figure can therefore confuse the physical meaning of the x-axis and compromise the validity of the comparison.

Accordingly, we have substantially revised the original Fig. 5 (now Fig. 7) and the associated text to remove this ambiguity and to ensure that **comparisons are made only between datasets obtained under consistent loading modes and stress definitions**:

1) **We separate bending-based and tensile-based datasets into two sub-figures (Fig. 7a and Fig. 7b).** This avoids the previous logical inconsistency of comparing bending and tensile results on a shared "cross-sectional area" axis, and ensures that each panel contains data that share the same loading condition and corresponding definition of reported strength.

2) **Fig. 7a now includes only bending-derived values** from the literature, reported consistently as the **maximum surface tensile stress** (or the equivalent tensile-side fracture stress) extracted using the methodology of the original references, together with our micro-cantilever bending results reported using the same definition. In this bending-only panel, the x-axis "cross-sectional area" is retained solely as a **practical size descriptor** to enable comparison across different specimen geometries. Importantly, for brittle failure under bending, the cross-sectional area serves as a reasonable indicator for the "effective stressed size": **as the cross-sectional area increases, the volume/area of material experiencing high tensile stress also increases, and therefore the probability that the high-stress region contains a critical defect correspondingly increases** (i.e., a

weakest-link size effect). This is precisely why cross-sectional area is used here as a convenient size descriptor in small-scale bending datasets. In contrast, alternatives such as **length or volume introduce greater ambiguity** because the literature employs different specimen shapes, aspect ratios, and boundary conditions, making those descriptors less comparable across studies.

3) **Fig. 7b now includes only tensile-derived values** from the literature (nanoscale tensile tests), together with our **microscale tensile results**. The stress is now plotted against the **gauge-section volume**. Specifically, we add our **conservative lower-bound values up to ~13 GPa** obtained from our micro-tensile experiments, where the gauge section reproducibly remains intact prior to grip-edge failure. While these lower-bound values do not represent the tensile fracture strength (due to premature failure at the grip), it provides an unambiguous tensile-loading-based **benchmark** demonstrating that micrometer-scale TiB_2 volumes can sustain exceptionally high uniform tensile stress without fracture, and it also highlights the current lack of comparable tensile data at the micrometer scale.

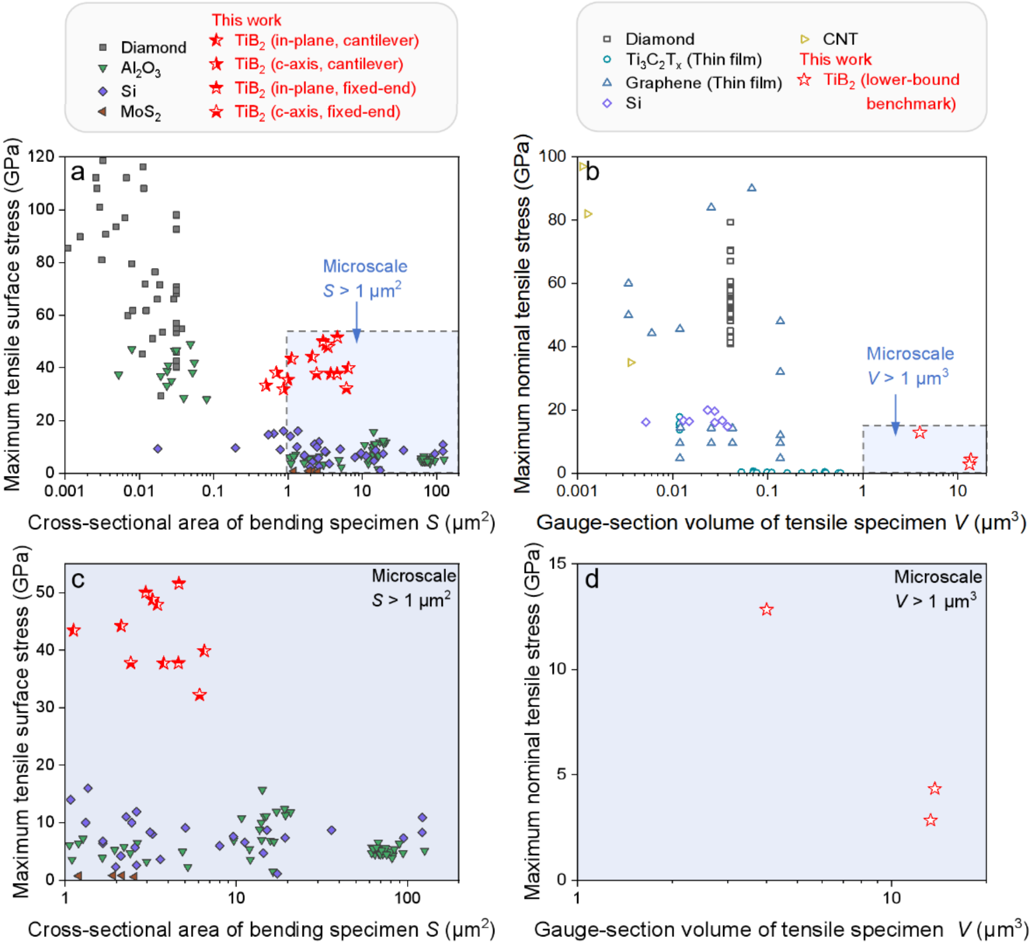

Figure 7. Strength limits of brittle solids as a function of specimen cross-sectional area, separated by loading mode to avoid mixing stress definitions. (a) Bending-derived strength data reported as the maximum tensile surface stress at failure, $\sigma_{I,max}$ (tensile-side fracture stress), plotted against cross-sectional area (used here as a practical length-scale descriptor). Our TiB_2 results are shown for two loading geometries (cantilever and fixed-end four-point bending) and two crystal directions (along the c-axis and within the basal plane). (b) Tensile-derived strength data from true uniaxial tensile tests, reported as the maximum nominal tensile stress plotted against the gauge-section volume. For our micrometer-scale TiB_2, the plotted value corresponds to the nearly uniform tensile stress (up to ~13 GPa) sustained within a large gauge volume (> 4 μm^3) immediately prior to grip-edge failure and therefore represents a conservative lower bound. (c) Enlarged view of panel (a) for the micrometer-scale regime with the x-axis starting at 1 μm^2. (d) Enlarged view of panel (b) for the micrometer-scale regime with the x-axis starting at 1 μm^3. Literature datasets include diamond [14, 15, 16], graphene [10, 11, 12, 13], carbon nanotubes (CNT) [9], $Ti_3C_2T_x$ [24, 25], Al_2O_3 [6, 26, 27, 28],

Si [7, 8, 29, 30] and MoS_2 [31]. Literature sources and the associated loading mode are listed in Supplementary Table 9.

With these changes, the revised Fig. 7 is logically consistent and unambiguous, while preserving its central message: **ultra-high strength can be achieved when TiB_2 is tested at micrometer-scale dimensions**, supported separately by bending-based evidence (Fig. 7a) and **tensile-based evidence** (Fig. 7b). Notably, in bending, our measured maximum surface tensile stress is multiple-fold higher than previously reported values in the same micrometer-scale regime (Fig. 7c). In tension, our measured stress (a conservative lower bound) directly **addresses a key gap** in the literature: no tensile strength data at micrometer-scale dimensions are available. To further enhance transparency, we have added **Supplementary Table 9**, which clearly identifies the testing mode for each literature data.

Accordingly, we added two paragraphs in the Results and discussion immediately before Fig. 7, to make the comparison logically consistent by separating loading modes and clarifying what "cross-sectional area" means in bending. We also clarified how our micro-tensile data are used as a conservative benchmark rather than a true gauge-section fracture strength.

(Page 15, line 12)

We summarize our TiB_2 results and compare them with literature data for brittle solids using consistent loading modes and stress/strain definitions. Accordingly, we separate bending-derived and tensile-derived datasets into two panels (Fig. 7a,b), with the data sources and their testing details summarized in Supplementary Table 9, thereby avoiding ambiguity that arises when "cross-sectional area" is used across fundamentally different stress states. Fig. 7 focuses on stress values for these mode-separated datasets, whereas the corresponding strain values for the same sources is provided in Supplementary Fig. 17. Fig. 7a includes only bending-type measurements, reported as the maximum tensile surface stress at failure, $\sigma_{\mathrm{I,max}}$ (i.e., the tensile-side fracture stress), as defined or extracted in the original references, together with our micro-cantilever and four-point bending results reported using the same

definition. In this bending-only panel, the x-axis "cross-sectional area" is retained solely as a practical length-scale descriptor: for brittle fracture under bending, a larger cross-section generally corresponds to a larger high-tensile-stress region and thus a higher probability of sampling a critical flaw (a weakest-link size effect). Consistent with this expectation, the literature data reveal a clear size effect: $\sigma_{\mathrm{I,max}}$ decreases as cross-sectional area increases, particularly once it exceeds ~1 μm^2. Despite this trend, our in situ-grown single-crystal TiB_2 sustains tensile strains up to 9.0% (Supplementary Fig. 17)—corresponding to $\sigma_{\mathrm{I,max}}$ ≈E/11—even at micrometer-scale cross-sections, thereby approaching the theoretical elastic limit. To date, such large elastic strains at this scale have only been reported for defect-minimized silicon fabricated by advanced semiconductor processing tested by bending[8], yet TiB_2 markedly outperforms silicon in $\sigma_{\mathrm{I,max}}$ by multiple factors due to its far higher modulus (Fig. 7c).

Fig. 7b compiles only tensile-type measurements from true uniaxial tensile tests, together with our micrometer-scale tensile benchmark for TiB_2. Notably, for brittle materials, successful reports of micrometer-scale tensile fracture stress with a truly micrometer-scale gauge volume remain essentially absent (Fig. 7d), largely because unavoidable grip-induced stress concentrations tend to dominate failure. Consistent with this limitation, our TiB_2 tensile specimens fail prematurely at the grip. Nevertheless, the gauge section with a large volume (> 4 μm^3) sustains a nearly uniform, very high tensile stress up to ~13 GPa prior to grip-edge failure. This value therefore provides a conservative lower bound and demonstrates that micrometer-scale TiB_2 volumes can sustain exceptionally high, near-uniform tensile stress without fracture, partially filling a key gap in the literature regarding the attainable tensile stress level for brittle materials at this length scale. Overall, these mode-separated comparisons reinforce a central message: when TiB_2 is formed in situ by eutectic solidification, micrometer-scale specimens can approach near-theoretical elastic limits because strength-limiting defects are exceedingly scarce.

We also added two short paragraphs in the Methods under "FEM analyses" to define the reported fracture strength in bending unambiguously and to explain the stressed-volume

quantification.

(Page 21, line 12)

For bending-based tests (cantilevers and fixed-end four-point bending), the reported “fracture strength” corresponds to the maximum tensile (principal) surface stress at the failure point, defined as the peak value of the maximum principal tensile stress on the tensile surface at the final stable loading increment immediately prior to the first cracking event. This is a local, FE-resolved surface stress in bending and should not be interpreted as a nominal uniaxial tensile stress averaged over the full cross-section. The corresponding maximum tensile strain was extracted from the same location and loading increment. For microscale tensile specimens, FE simulations incorporating the measured grip alignment were used to quantify the stress distribution and to identify the nearly uniform tensile stress sustained in the gauge section immediately prior to grip-edge failure (Supplementary Fig. 14).

To explicitly quantify the stressed volume associated with high tensile stresses in bending, we post-processed FE stress fields to compute the cumulative TiB_2 volume exceeding a chosen tensile-stress threshold by integrating element volumes as a function of maximum principal tensile stress. This stress-volume analysis was performed for representative cantilever beams with the largest cross-sections to directly assess the micrometer-scale volume subjected to ultrahigh tensile stress.

Finally, in the revised Supplementary Information we added Supplementary Note S2 to directly address the “stressed region vs. cross-section” concern. We thank the reviewer again for highlighting this issue; addressing it has significantly improved the logic and clarity of the manuscript.

Comment 4: *What are the error bars in Figure 3 represent? My understanding is there are only 10 samples. Are the error bars in single sample correspond to measurement error?*

Response: Thank you for pointing this out. The error bars in Fig. 3 do not represent specimen-to-specimen scatter. Instead, each data point corresponds to one cantilever test, and the associated error bar represents the **estimated uncertainty in the FE-extracted maximum tensile fracture stress/strain for that specific specimen**. Because our fracture strength is obtained by combining the experimental load–displacement curve with specimen-specific FE modeling, the uncertainty primarily reflects the sensitivity of the extracted peak tensile stress/strain to several independent modeling and measurement inputs. We quantified three dominant contributions to the uncertainty and added the corresponding analyses in the revised Supplementary Information:

1) **Influence of matrix compliance beneath the cantilever**. As shown in Supplementary Fig. 18, retaining sufficient matrix volume beneath the cantilever ensures that TiB_2 carries essentially all of the bending load. Replacing a rigid substrate with an elastoplastic matrix changes the extracted fracture stress by only ±1%, indicating that matrix deformation contributes negligibly to the uncertainty.

2) **Friction coefficient at the indenter–specimen contact**. We performed contact simulations over a broad and conservative range of friction coefficients f = 0.1-1.0; Supplementary Fig. 19). The resulting maximum tensile stress/strain varies by only ±4%, demonstrating that the extracted fracture values are weakly sensitive to reasonable boundary-condition assumptions.

3) Geometrical measurement uncertainty. The specimen width and height are measured from SEM images, with edge-definition uncertainty on the order of ±30 nm. Propagating this dimensional perturbation through the FE analysis changes the extracted fracture strain (and the corresponding stress) by ±3% (Supplementary Fig. 20).

Combining these contributions leads to an overall uncertainty of approximately ±8% in the reported fracture strength/strain for each specimen, which is what is shown as the error bars in Fig. 3.

Accordingly, we added one clarifying sentence in the Results and discussion in the paragraph that introduces Fig. 3, explicitly stating what the error bars mean.

(Page 8, line 30)

The error bars indicate the estimated uncertainty for each individual specimen in the FEM-extracted $\sigma_{\mathrm{I,max}}$ and $\varepsilon_{\mathrm{I,max}}$ (see Supplementary Note S3 for details).

We also revised the Fig. 3 caption by adding one sentence that defines the error bars as specimen-specific propagated uncertainty (not scatter).

(Page 9, line 9)

Figure 3. Plot of maximum tensile surface stress at failure versus corresponding maximum tensile surface strain for Type-I and Type-II micro-cantilever and four-point bending beams, revealing only moderate anisotropy. Each symbol corresponds to one test. $\sigma_{\mathrm{I,max}}$ and $\varepsilon_{\mathrm{I,max}}$ are the maximum tensile surface stress and strain at the failure point, extracted from specimen-specific FEM calibrated by the measured load-displacement curve. Error bars represent the estimated specimen-specific analysis/measurement uncertainty (±8%), obtained by propagating the sensitivities to (i) matrix-support compliance, (ii) indenter-specimen friction, and (iii) SEM dimensional measurement uncertainty (Supplementary Note S3).

In addition, we added one new paragraph in the Methods (under "FEM analyses") that describes how the uncertainty was quantified and combined into ±8%:

(Page 21, line 3)

To estimate the uncertainty shown as error bars in Fig. 3, we quantified the sensitivity of the FEM-extracted $\sigma_{\mathrm{I,max}}$ and $\varepsilon_{\mathrm{I,max}}$ to three dominant inputs: (i) compliance of the supporting

matrix beneath the TiB_2 beam (rigid versus elastoplastic substrate), (ii) the indenter-specimen Coulomb friction coefficient (f = 0.1–1.0), and (iii) SEM-based dimensional measurement uncertainty of beam width and height (±30 nm edge-definition uncertainty). The resulting variations were ±1%, ±4%, and ±3%, respectively (Supplementary Figs. 18-20), which we conservatively summed to obtain an overall ±8% uncertainty per specimen (Fig. 3).

Finally, in the revised Supplementary Information we added Supplementary Note S3 and the associated new sensitivity figures (Supplementary Figs. 18-20) that explicitly document each uncertainty contribution.

We thank the reviewer again for helping us clarify this important point.

Comment 5: *Please provide more details on the eutectic reactions forming the TiB_2 particles in-situ. This is one of the novel parts of this paper but there are not enough details to reproduce this method.*

Response: We thank the reviewer for suggesting additional details on the in-situ formation of TiB_2. In our study, micrometer-scale TiB_2 forms during casting via eutectic/near-eutectic solidification in the Fe–Ti–B system (Ti/B-enriched high-modulus steels, HMS-1 and HMS-2; compositions are provided in the Supplementary Information). The resulting faceted hexagonal-prismatic TiB_2 morphology is shown in Supplementary Fig. 1.

More specifically, the formation pathway is as follows. (i) **Solute enrichment prior to solidification**: after Ti and B are added to the melt, their limited solubility in Fe and strong chemical affinity drive local Ti–B enrichment in the liquid, creating Ti–B–rich liquid pockets as cooling proceeds. (ii) **Primary TiB_2 formation from a hypereutectic liquid**: upon the onset of solidification, primary single-crystal TiB_2 precipitates directly from these Ti–B–rich regions (hypereutectic solidification), and continued TiB2 growth progressively depletes Ti and B from the surrounding melt, rendering the remaining liquid increasingly Fe-rich. This

can be expressed schematically as $L_{(Ti,\ B\ rich)}$ → TiB_2 + $L_{(Fe\ rich)}$. The characteristic hexagonal-prismatic morphology arises from anisotropic growth kinetics dictated by the TiB2 crystal structure: growth along close-packed planes is slow because fewer atomic attachment sites are available, whereas less close-packed (inclined) planes advance faster and are eventually eliminated, leaving the slow-growing basal (0001) and prismatic {10-10} facets as the dominant particle surfaces, consistent with Supplementary Fig. 1. (iii) **Secondary TiB_2 during final solidification**: as the residual Fe-rich liquid solidifies to the steel matrix, Ti and B are rejected to the solidification front and additional fine TiB_2 forms by the terminal eutectic reaction, $L_{(Fe\ rich)}$ → TiB_2 + Fe, completing the in-situ TiB_2 dispersion.

Accordingly, we added one sentence in the Results and discussion to explicitly point readers to the detailed eutectic/near-eutectic formation sequence:

(Page 3, line 24)

We synthesized micrometer-scale TiB_2 via eutectic solidification within Fe-Ti-B-based high-modulus steel systems[21]. In these Ti/B-enriched steels, TiB_2 forms in situ during casting through a Fe-Ti-B eutectic/near-eutectic (often hypereutectic) solidification sequence, which is provided in Supplementary Note S1.

We also added one new paragraph in the Methods, under "Fabrication of micromechanical TiB_2 specimens," to describe the key eutectic/near-eutectic formation pathway in a reproducible manner.

(Page 18, line 25)

…TiB_2 is generated during casting by eutectic/near-eutectic solidification in the Fe-Ti-B system. During cooling, Ti and B exhibit limited solubility in Fe and partition to form Ti-B-enriched liquid regions; TiB_2 precipitates from these regions as primary faceted crystals. As solidification proceeds, the remaining Fe-rich liquid transforms into the steel matrix while preserving the already-formed TiB_2 (Supplementary Note S1), yielding the partially embedded hexagonal-prismatic TiB_2 morphology observed after deep etching

(Supplementary Fig. 1). Sample surfaces were prepared by sequential grinding (80–3000 grit), polishing with diamond suspensions, and final vibratory polishing with a 50-nm SiO_2 colloidal suspension. TiB_2 was then exposed by deep-etching in 33 vol% HCl.

Finally, we added Supplementary Note S1 in the revised Supplementary Information, which lays out the eutectic/near-eutectic sequence explicitly.

Comment 6: In videos of Samples B3 and B5, there is cracking near the bottom attachment point of the samples close the fracture. I think the samples should be considered as failed at this point, and the stresses at the crack initiation instance should be reported as the fracture strengths. Can you please clarify what is happening in these samples?

Response: Thank you for carefully inspecting the videos of B-3 and B-5 and for raising an important point about the cracking observed near the lower (compressed) corner region shortly before final fracture. We agree that this behavior and our strength-definition convention should be clarified more explicitly.

To address this, we added Supplementary Fig. 4, which includes (i) the complete experimental load–displacement curves of B-3 and B-5 and (ii) corresponding SEM snapshots captured at the moment when **the first detectable microcracks** appear near the bottom (compressive) surface close to the fixed end. As shown in this figure, the first cracks in B-3 and B-5 initiate at the compressive side rather than at the tensile surface. This early cracking event produces a **sudden but limited** load drop in the load-displacement curve (i.e., a brief stress relaxation rather than catastrophic beam failure). Mechanistically, this behavior is consistent with the cantilever bending stress state: the region above the neutral plane is in tension, whereas the lower region is in compression, where cracking can occasionally nucleate locally under very high geometric stress concentration at the lower corner. Importantly, compression suppresses crack opening, so such cracks **remain localized** instead

of immediately triggering global tensile failure.

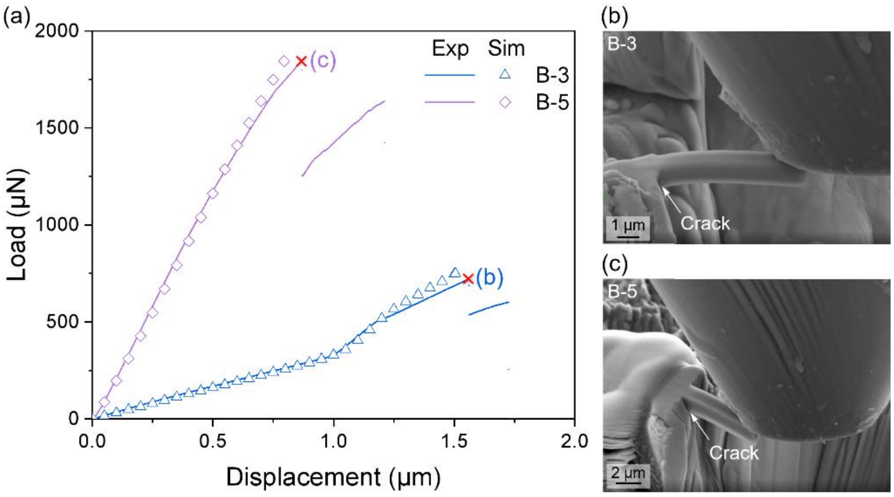

Supplementary Figure 4: Crack initiation on the compressed lower surface in B-3 and B-5 and definition of the failure point used for FEM extraction. (a) Complete experimental force-displacement curves (solid lines) compared with FEM simulations (symbols) for B-3 and B-5. The cross symbol marks the displacement corresponding to the last stable increment immediately before the first load drop, which is used to extract $\sigma_{\mathrm{I,max}}$ and $\varepsilon_{\mathrm{I,max}}$. (b,c) Representative SEM video frames captured at the moment when the first detectable microcrack appears near the bottom (compressive) corner close to the fixed end in (b) B-3 and (c) B-5.

Regarding the reviewer's suggestion that the specimen should be considered failed at the first crack initiation, we clarify that we **indeed take this first crack event as the failure time-point** and extract the stress state at that moment. However, the relevant quantity depends on the failure mode being quantified. In B-3 and B-5, this first event represents a compressive-side damage threshold specific to the highly constrained fixed-end corner, rather than the intrinsic tensile fracture limit that our analysis is designed to quantify. Therefore, in the present work, we report the **maximum tensile stress at the upper surface at the instant immediately preceding the first load drop**, i.e., the peak elastic load sustained prior to any detectable cracking. Using the FE analysis, the corresponding peak tensile stresses are 35.5 GPa (B-3) and 43.4 GPa (B-5).

We report the tensile-side stress rather than the compressive-side stress for three reasons. ***First***, the objective of this micromechanical test series is to quantify the **tensile fracture resistance** of micrometer-scale TiB_2, for which tensile opening is the relevant mode in defect-controlled brittle fracture. In contrast, compressive cracking is strongly geometry- and constraint-dependent and does not directly test our central hypothesis of "large TiB_2 without critical defects." ***Second***, defining the strength at the peak elastic load ensures a **uniform and consistent criterion across all beams**. ***Third***, the absolute compressive stresses at the sharp bottom corner are more prone to **numerical singularity artifacts** in finite-element analysis and thus carries larger uncertainty, whereas the tensile-side stress is extracted more robustly.

Accordingly, throughout the manuscript we define and report "fracture strength" based on the **tensile-side limit** at the onset of the first cracking event. We also acknowledge that for B-3 and B-5, these tensile values should be interpreted as **conservative (lower) estimates** of the intrinsic tensile fracture strength, because the tests were interrupted by compressive-side cracking before a tensile-side fracture event occurred. Nevertheless, these conservative values remain well within the "tens of GPa" regime and are sufficient to support our key conclusion.

In the revised manuscript, we added two sentences in the Results and discussion (in the paragraph describing the load-displacement curves in Fig. 2e,f) to explicitly describe this occasional compressive-side crack initiation in B-3 and B-5, clarified the definition of the failure point while maintaining consistency across all specimens.

(Page 6, line 19)

In addition, two beams (B-3 and B-5) showed a small, abrupt load drop shortly before final failure (Supplementary Fig. 4), coincident with the first appearance of a microcrack at the highly constrained lower corner on the compressive side near the fixed end (Supplementary Videos). Accordingly, throughout this work we define the failure point used for FEM extraction as the last stable loading increment immediately preceding catastrophic fracture, or, if present, preceding the first detectable cracking event (for B-3 and B-5).

In addition, the caption of Supplementary Figure 4 specifies that "The cross symbol marks the displacement corresponding to the last stable increment immediately before the first load drop, which is used to extract $\sigma_{\mathrm{I,max}}$ and $\varepsilon_{\mathrm{I,max}}$".

We also revised the Figure 2 caption to make this criterion explicit in the figure description.

(Page 7, line 6)

…(e, f) Experimentally measured force-displacement curves (solid lines) compared with FEM simulations (symbols) for Type I (e) and Type II (f) beams, plotted up to the failure point (cross symbol). For B-3 and B-5, the failure point is defined by the first detectable cracking event at the compressed lower corner; for all other cantilevers, it corresponds to the onset of catastrophic fracture.

We further added one new paragraph in the Methods (under "FEM analyses") to formalize the same definition and clarify what "fracture strength" means for bending-based tests.

(Page 21, line 12)

For bending-based tests (cantilevers and fixed-end four-point bending), the reported "fracture strength" corresponds to the maximum tensile (principal) surface stress at the failure point, defined as the peak value of the maximum principal tensile stress on the tensile surface at the final stable loading increment immediately prior to the first cracking event.

Finally, in the revised Supplementary Information, we added one paragraph in Supplementary Note S3 to document the B-3 and B-5 crack-onset convention and the resulting tensile-side stresses, which reads: "Furthermore, in two cantilever tests (B-3 and B-5), the first detectable crack appears on the compressed lower surface near the fixed end

and produces a sudden but limited load drop (Supplementary Fig. 4). To apply a uniform and conservative criterion across all beams, the FEM-extracted $\sigma_{\mathrm{I,max}}$ and $\varepsilon_{\mathrm{I,max}}$ for these two specimens are evaluated at the last stable loading increment immediately before this first load drop (i.e., prior to any detectable cracking). Using this convention, the peak tensile-side stresses are 35.5 GPa (B-3) and 43.4 GPa (B-5)."

Reviewer #2:

General comment: *The authors employ eutectic solidification in steel metallurgy to synthesize a rigid, micrometer-scale TiB_2 ceramic phase. Mechanical properties are evaluated through micrometer-scale bending tests and compared with measurements from cantilever and C-shaped specimens. The experiments are carefully conducted; however, several aspects of the interpretation require clarification.*

Response: Thank you for your positive assessment of our manuscript. We agree that several aspects of the interpretation would benefit from clearer explanation. Below are our responses.

Comment 1: *The expression "tensile fracture strength" is potentially misleading. The tensile stress reported here is derived from FEM simulations of bending, rather than from direct tensile loading. Because bending introduces a complex stress state, the actual tensile component is uncertain and may be overestimated. It would be more accurate to describe the reported value as the "maximum surface stress estimated by FEM."*

Response: Thank you for this important suggestion. We agree that the term "tensile fracture strength" can be misinterpreted as a value obtained from a direct uniaxial tensile test, whereas in our work the stress is **calculated from micro-cantilever bending using finite-element method (FEM)**. To remove this ambiguity, we have revised the manuscript by replacing "tensile fracture strength" with "**maximum tensile surface stress at fracture (estimated by FEM)**" throughout the text, Supplementary Information, and the relevant figure labels/captions (e.g., Figs. 3 and 5).

In cantilever bending, the region above the neutral plane is in tension while the region below is in compression. In addition, the tensile stress is heterogeneous and reaches its maximum at the **tensile (upper) surface near the fixed end**. Accordingly, for each micro-cantilever we report the maximum tensile surface stress on the upper surface immediately prior to detectable cracking (i.e., at the peak load defining the onset of failure). We focus on the tensile surface because tensile stress governs crack opening and brittle failure, whereas compressive stress does not promote crack opening. We also clarify in the revised text that compressive stresses at the sharp bottom corner can be more sensitive to local stress gradients and potential numerical singularities in FEM. Therefore, compared to the lower-surface maximum compressive stress, using the upper-surface maximum tensile stress provides a more reliable and conservative estimate of the intrinsic fracture strength. We have added an explicit statement in the Methods/main text emphasizing that bending produces a non-uniform stress state, and that our reported value represents the FEM-estimated maximum tensile surface stress, rather than a uniaxial tensile stress as in a standard tensile test.

To address the reviewer's concern about uncertainty or possible overestimation, we clarify in the revised text that the FEM-based stress extraction is quantitatively constrained by the excellent agreement between simulated and experimental pre-fracture force-displacement curves, obtained using a single, physically consistent set of anisotropic elastic constants (consistent with literature values). Furthermore, the uncertainty in the FEM-estimated maximum tensile surface stress is explicitly reflected in the error bars (Fig. 3), which account for sensitivity to beam geometry, boundary conditions, and load calibration. We also added explicit statements in the Results/figure captions at first mention that the stresses are FEM-estimated surface stresses.

Finally, as described in our response to Reviewer 1, we attempted direct micrometer-scale tensile testing by fabricating single-crystal TiB_2 tensile specimens and loading them using PFIB-machined diamond grips. However, fracture occurred reproducibly at the grip edge due to unavoidable stress concentration, preventing reliable extraction of intrinsic uniaxial tensile strength from the gauge section. This practical limitation motivated our use of

micro-cantilever bending combined with FEM for stress quantification.

Accordingly, we added one explicit clarifying sentence in the Results and discussion to avoid any ambiguity that the reported stress is a nominal tensile strength.

(Page8, line 16)

…Notably, $\sigma_{\mathrm{I,max}}$ is a local, FEM-resolved tensile-side surface stress under bending and should not be interpreted as a nominal tensile stress averaged over the full cross-section of a uniaxial tensile specimen…

In the same paragraph, we also made the definition unambiguous at first use by stating:

(Page8, line 4)

where a maximum (principal) tensile surface strain $\varepsilon_{\mathrm{I,max}}$ of 7.7% corresponds to a maximum (principal) tensile surface stress at failure of $\sigma_{\mathrm{I,max}}$ = 48.8 GPa.

We also updated the figure caption and axis wording so the terminology is consistent throughout, replacing "tensile fracture strength" with the more accurate bending-derived terminology. For instance, the revised caption of Figure 3 now reads:

(Page 9, line 9)

Figure 3. Plot of maximum tensile surface stress at failure versus corresponding maximum tensile surface strain for Type-I and Type-II micro-cantilever and four-point bending beams, revealing only moderate anisotropy. Each symbol corresponds to one test. $\sigma_{\mathrm{I,max}}$ and $\varepsilon_{\mathrm{I,max}}$ are the maximum tensile surface stress and strain at the failure point, extracted from specimen-specific FEM calibrated by the measured load-displacement

curve…

We further added one new paragraph in the Methods (under “FEM analyses”) that formally defines what we report as “fracture strength” in bending and reiterates that it is not a nominal uniaxial tensile stress. The added Methods paragraph reads:

(Page 21, line 12)

For bending-based tests (cantilevers and fixed-end four-point bending), the reported “fracture strength” corresponds to the maximum tensile (principal) surface stress at the failure point, defined as the peak value of the maximum principal tensile stress on the tensile surface at the final stable loading increment immediately prior to the first cracking event. This is a local, FE-resolved surface stress in bending and should not be interpreted as a nominal uniaxial tensile stress averaged over the full cross-section…

Finally, we added the corresponding definition in Supplementary Note S2, to make the stress definition consistent and explicit in the Supplementary framework as well. The added Supplementary Note text states: “We use the maximum principal stress σ_I and restrict the analysis to tensile values. This choice is consistent with brittle fracture being controlled by crack opening under tension and with the stress definition reported throughout the manuscript…”

Comment 2: *Bending tests combined with FEM analysis have previously been applied to micro-sized diamond, revealing tensile strains up to ~9% and stresses of ~89–98 GPa (Science 360, 300–302, 2018). The authors should better articulate the novelty and significance of their findings on* TiB_2 *ceramics in comparison with these prior studies.*

Response: We thank the reviewer for pointing out the relevant diamond benchmark extracted using micro-bending combined with FEM (Science 360, 300–302, 2018). This study, together

with related works, was already cited in the Introduction and included as literature data in original Figure 5 (now Figure 7 in the revised manuscript). In the revision, we cite it more explicitly and clarify the specimen scale: although the diamond specimens are micrometer-long, they are typically nanoneedle-like nanostructures with **nanoscale cross-sections, and therefore their stressed volumes remain nanoscale**. Accordingly, we now distinguish specimen length from cross-sectional (stressed-volume) scale in both the revised manuscript and Supplementary Table 9. Our key advance is that eutectic-grown TiB_2 reaches similarly large elastic strains while **being truly micrometer-scale in cross-section/stressed volume**, enabled by a **low-cost, scalable metallurgical synthesis route** rather than semiconductor-style defect minimization.

Firstly, the relevant size is the cross-sectional dimension (which controls surface stress and flaw population) rather than beam length. Our TiB_2 specimens possess cross-sectional dimensions on the order of micrometers, i.e., an order-of-magnitude larger cross-sectional area than the thin, whisker-like geometries typically required to approach ideal behavior. That is, our study explicitly shows that near-theoretical response is not confined to ultra-thin or quasi-nanoscale specimens.

Equally importantly, the formation pathway is central to the broader significance. The diamond benchmark relies on an intrinsically exceptional covalent crystal combined with specialized high-purity synthesis and microfabrication. In contrast, the TiB_2 studied here forms in situ by eutectic solidification within steel, i.e., via a low-cost, industrially scalable casting route that can inherently suppress critical defects during growth. This establishes a distinct paradigm: near-theoretical strength can be realized at the microscale through defect control during solidification and growth, rather than relying on an extraordinary crystal system and/or advanced nanoscale fabrication process.

Finally, we note that TiB_2 is not only a brittle ceramic but also a good electrical conductor, which differentiates it from diamond and expands the relevance of achieving near-theoretical mechanical behavior to applications where simultaneous mechanical robustness and electrical conductivity are required (e.g., conductive wear-resistant components and electrically

functional ceramic reinforcements).

Accordingly, we have revised the Introduction to more explicitly position our TiB_2 results relative to the diamond "micro-bending + FEM" benchmark by distinguishing specimen length from cross-sectional (stressed-volume) scale.

(Page 2, line 16)

…These nanoscale specimens offer the opportunity to observe ultrahigh strength and large elastic strains close to the theoretical limit through nanomechanical testing, primarily via bending or tension. For instance, high-quality chemical-vapor-deposited diamond[14, 15] has been fabricated into nanoneedles with nanoscale cross-sections and tested by bending, yet the stressed volume remains nanoscale even when the overall specimen length is micrometric. Uniaxial tensile tests[16] on similarly prepared diamond also confine the gauge cross-section to the nanometer scale. Clearly, achieving such near-theoretical properties in truly microscale specimens with micrometer-scale cross-sections remains exceptionally challenging…

In the next paragraph, we added the explicit novelty statement:

(Page 3, line 5)

…This easily synthesized ceramic phase achieves maximum tensile surface stresses at failure exceeding 30 GPa and tensile elastic limits up to 9.0% in large specimens with micrometer-scale cross-sections under bending, distinct from the commonly reported micron-long nanoneedles, whose cross-sections (and thus stressed volumes) remain nanoscale…

Finally, in the revised Supplementary Information we updated Supplementary Table 9 to transparently document the diamond benchmark together with its loading mode and size descriptor, in comparison with our TiB_2 dataset.

Comment 3: *The term "ideal strength" is not appropriate in this context. It specifically refers to the theoretical strength of a defect-free crystal, which is not the case in the present work.*

Response: Thank you for this important clarification. We agree that "ideal strength" refers to the maximum stress a perfectly defect-free crystal can withstand at 0 K, and that our room-temperature measurements on real micrometer-scale TiB_2 particles should not be presented as the exact ideal limit. In this context, the more precise term is the theoretical strength limit of a defect-free crystal, commonly of order ~E/10 (where E is Young's modulus), which serves as a benchmark rather than a directly measurable value in real specimens.

To ensure accurate and non-misleading terminology, we have **thoroughly revised the manuscript** to (i) replace "ideal strength" with "theoretical strength" when referring to the defect-free benchmark (at room temperature), and (ii) consistently describe experimentally measured values (including our results) as "near-theoretical" or "approaching the theoretical limit", rather than as the theoretical limit itself, **throughout the main text, Supplementary Information, and figure captions**. Because these terminology updates occur in many places, we do not enumerate every instance in this response letter.

In addition, we revised the title by replacing "ideal" with "near-theoretical". The title now becomes: Approaching near-theoretical strength and ultra-large elasticity in micrometer-scale TiB_2 ceramics

Finally, we added a clarifying sentence at the end of the paragraph describing cyclic loading on an additional C-shaped specimen, explicitly stating that our conclusion is an exceptionally low population of strength-limiting defects rather than the existence of perfectly defect-free crystals.

(Page 12, line 27)

…We also emphasize that this does not imply a perfect crystal; rather, it indicates that

strength-limiting defects are essentially absent at the micrometer scale.

These changes improve precision without altering data, analyses, or the central conclusion that micrometer-scale, in-situ-grown TiB_2 can achieve exceptionally high elastic strain and strength levels that are close to theoretical expectations.

Supplementary References

1. Dang C*, et al.* Achieving large uniform tensile elasticity in microfabricated diamond. *Science* **371**, 76-78 (2021).
2. Dong LR*, et al.* Borrowed dislocations for ductility in ceramics. *Science* **385**, 422-427 (2024).
3. Qiu K*, et al.* Self-healing of fractured diamond. *Nature Materials* **22**, 1317-1323 (2023).
4. Nie A*, et al.* Approaching diamond's theoretical elasticity and strength limits. *Nat Commun* **10**, 5533 (2019).
5. Mueller MG, Fornabaio M, Žagar G, Mortensen A. Microscopic strength of silicon particles in an aluminium–silicon alloy. *Acta Materialia* **105**, 165-175 (2016).
6. Žagar G, Pejchal V, Mueller MG, Rossoll A, Cantoni M, Mortensen A. The local strength of microscopic alumina reinforcements. *Acta Materialia* **100**, 215-223 (2015).
7. Banerjee A*, et al.* Ultralarge elastic deformation of nanoscale diamond. *Science* **360**, 300-302 (2018).
8. Cao K*, et al.* Elastic straining of free-standing monolayer graphene. *Nat Commun* **11**, 284 (2020).
9. Feng S*, et al.* Experimentally measuring weak fracture toughness anisotropy in graphene. *Communications Materials* **3**, (2022).
10. Jaddi S*, et al.* Definitive engineering strength and fracture toughness of graphene through on-chip nanomechanics. *Nat Commun* **15**, 5863 (2024).
11. Peng B*, et al.* Measurements of near-ultimate strength for multiwalled carbon nanotubes and irradiation-induced crosslinking improvements. *Nature nanotechnology* **3**, 626-631 (2008).
12. Rong C*, et al.* Elastic properties and tensile strength of 2D Ti(3)C(2)T(x) MXene monolayers. *Nat Commun* **15**, 1566 (2024).
13. Firestein KL*, et al.* Young's Modulus and Tensile Strength of Ti(3)C(2) MXene Nanosheets As

Revealed by In Situ TEM Probing, AFM Nanomechanical Mapping, and Theoretical Calculations. *Nano Lett* **20**, 5900-5908 (2020).

14. Pejchal V, Fornabaio M, Žagar G, Mortensen A. The local strength of individual alumina particles. *Journal of the Mechanics and Physics of Solids* **109**, 34-49 (2017).

15. Feilden E*, et al.* Micromechanical strength of individual Al2O3 platelets. *Scripta Materialia* **131**, 55-58 (2017).

16. Wang S*, et al.* An improved loop test for experimentally approaching the intrinsic strength of alumina nanoscale whiskers. *Nanotechnology* **24**, 285703 (2013).

17. Mueller MG, Žagar G, Mortensen A. In-situ strength of individual silicon particles within an aluminium casting alloy. *Acta Materialia* **143**, 67-76 (2018).

18. Zhang H*, et al.* Approaching the ideal elastic strain limit in silicon nanowires. *Science advances* **2**, e1501382 (2016).

19. Colas G, Serles P, Saulot A, Filleter T. Strength measurement and rupture mechanisms of a micron thick nanocrystalline MoS2 coating using AFM based micro-bending tests. *Journal of the Mechanics and Physics of Solids* **128**, 151-161 (2019).

20. Liu J, Li Y, Zhuang Q, Huang M, Xu W. Low-density and high-modulus steel achieved by hypereutectic TiB2 and high Al ferrite phase. *Journal of Materials Science & Technology*, (2025).

21. Li B*, et al.* On the fatigue crack propagation mechanism of a TiB2-reinforced high-modulus steel. *Composites Part B: Engineering* **190**, 107960 (2020).

22. Huang MX, He BB, Wang X, Yi HL. Interfacial plasticity of a TiB 2 -reinforced steel matrix composite fabricated by eutectic solidification. *Scripta Materialia* **99**, 13-16 (2015).

Response to Reviewers

Dear Editor and Reviewers,

We sincerely thank you for your continued time, careful evaluation, and constructive feedback on our manuscript NCOMMS-25-71116A, "**Approaching near-theoretical strength and ultra-large elasticity in micrometer-scale TiB_2 ceramics**". We are pleased to submit this second-round revised manuscript.

We are encouraged that the first-round revision was received positively and that the manuscript was considered to have been substantially improved. In particular, Reviewer 1 indicated that all comments and suggestions had been addressed successfully, and Reviewer 2 noted that most of the previous comments had been addressed. In this second revision, we have carefully addressed the remaining points, further strengthened the presentation and interpretation, and performed additional compression tests to provide further experimental support for our conclusions.

Each reviewer comment is copied below in *italic*. Our responses are provided in blue, and the corresponding revisions to the manuscript are highlighted in red.

Reviewer #1:

General comment: *I thank the authors for their very detailed, elaborate, and well-written response letter. I think this is the best response letter I have ever seen. I really appreciate the new tests, data, and discussion. All my comments and suggestions are addressed successfully.*

Response: Thank you very much for your highly positive and encouraging assessment. We are especially honored by your remark that this is "the best response letter". We also sincerely appreciate your recognition of our new tests, data, and discussion, and we are very pleased that you find all of your comments and suggestions to have been addressed successfully.

Comment 1: *I just have a minor question on the Supplementary note S2 and supplementary figure 5. I guess I did not understand the procedure, but to me the binned volume should be*

bigger than the cumulative stressed volume. A clarification regarding this issue will be helpful to have in the supplementary file.

Response: Thank you very much for this careful observation. We agree that, in the previous version, Supplementary Note S2 and Supplementary Figure 5 did not present the stress-volume analysis as clearly as they could, and we appreciate the opportunity to improve this point further.

The source of confusion is that the original figure combined two different quantities in a single plot: the binned stress-volume data and the cumulative-above-threshold stressed volume. Although these two quantities are mathematically related, they convey different information. The binned data describe how much TiB_2 volume falls within each individual stress interval, whereas the cumulative quantity gives the total TiB_2 volume subjected to stresses higher than a chosen threshold. Because these two types of information were previously overlaid in the same panel, we agree that the distinction was not as immediate or self-evident as it should be.

To make the figure more intuitive and easier to read, **we have further revised Supplementary Figure 5 by separating the two quantities into two panels**. In the revised Supplementary Fig. 5(a), we show only the bin data, with the x-axis labeled “Tensile stress” and the y-axis labeled “Volume in each stress bin.” In the revised Supplementary Fig. 5(b), we show only the cumulative (threshold) stressed volume, with the x-axis labeled “Stress threshold” and the y-axis labeled “TiB_2 volume above threshold.” In addition, to make the figure more immediately understandable to a broad readership, **we further avoid the use of mathematical symbols and expressions and present the quantities directly in plain language**. In this way, the reader can immediately see that panel (a) reports the volume within each single stress interval, whereas panel (b) reports the total accumulated volume above a selected stress threshold. This separation avoids mixing the two types of information and makes the figure much more self-explanatory.

With this revised presentation, the specific point raised by the reviewer becomes immediately clear. For example, the value at 15 GPa in panel (b) is not the volume of one bin at 15 GPa. Rather, it is the sum of all TiB_2 element volumes with local tensile stress higher than 15 GPa.

By contrast, panel (a) shows only the volume contained within each individual stress bin. Therefore, the cumulative quantity in panel (b) is naturally expected to be larger than any single-bin value in panel (a) at the same stress level.

We have accordingly revised both Supplementary Note S2 and the caption of Supplementary Figure 5 to match this clearer two-panel presentation. These revisions do not change the conclusion of the analysis. They simply make the procedure and the physical meaning of the plotted quantities more transparent. The key conclusion remains the same: even in cantilever bending, the TiB_2 volume subjected to very high tensile stress is already micrometer-scale, which supports our argument that the present TiB_2 contains an exceptionally low population of strength-limiting defects.

Revisions in Supplementary Information:

To further improve clarity, we have revised Supplementary Note S2 and Supplementary Figure 5 so that the two stress-volume quantities are now presented separately and in the same order as they are discussed.

1) In Supplementary Note S2, we now more explicitly distinguish between the binned stress-volume data and the cumulative-above-threshold stressed volume, and clarify that the latter is obtained by summing all element volumes above a chosen stress threshold. We also replace the original mathematical symbols and expressions by plain-language descriptions to make the descriptions easier to understand.

(Page S3, line 9)

… Two different but related quantities were then extracted from the FEM results. For each FEM mesh, we post-process all elements and calculate: (i) the TiB_2 volume in each stress bin, obtained by summing element volumes whose local tensile stress falls within each individual stress interval. This quantity is shown in Supplementary Fig. 5a for beam B-9; and (ii) the TiB_2 volume above a chosen stress threshold, obtained by summing all TiB_2 element volumes whose local tensile stress is higher than that threshold. This quantity is shown in Supplementary Fig.

5b for beam B-9. These two quantities answer different questions. Supplementary Fig. 5a shows how much TiB_2 volume lies within each individual stress interval, whereas Supplementary Fig. 5b shows the total TiB_2 volume subjected to stresses higher than a selected threshold.

…the cumulative volume above a high-stress threshold of 15 GPa exceeds 5 μm³. …

…because failure at such high tensile stress sustained over μm³-scale volumes would otherwise be unlikely under a weakest-link scenario.

2) We revised Supplementary Figure 5 and its caption accordingly. Specifically, we separate the previous figure into two panels to make the figure more immediately self-explanatory. In addition, we avoid the use of mathematical symbols and expressions in the figure and instead use plain-language descriptions to make the figure easier to understand.

(Page S9, line 1)

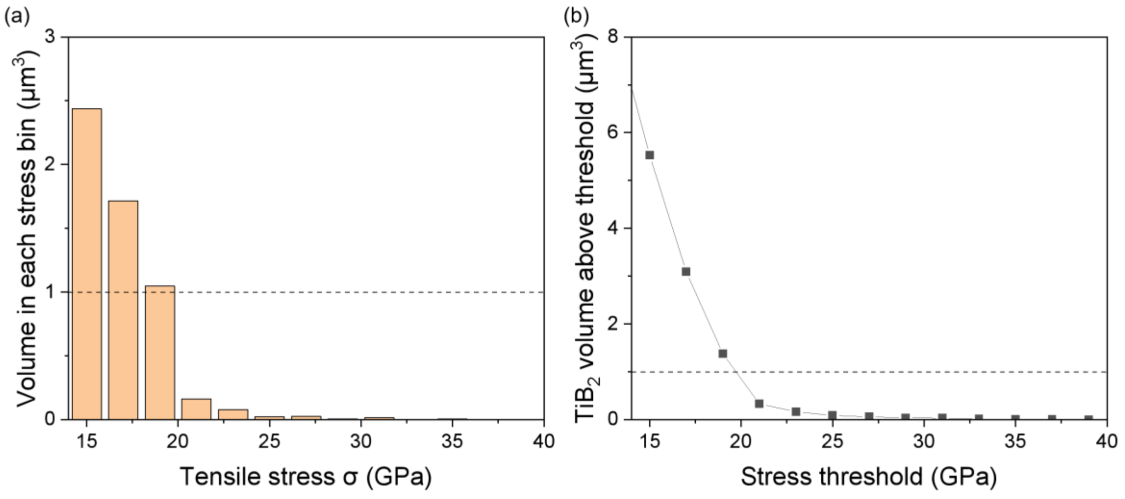

Supplementary Figure 5: Stress-volume analysis for the representative cantilever beam B-9. (a) Volume in each stress bin, showing the TiB_2 volume contained within each individual tensile-stress interval. (b) TiB_2 volume above threshold, showing the cumulative TiB_2 volume subjected to tensile stress higher than a selected threshold. The analysis shows that the TiB_2 volume subjected to very high tensile stress is already micrometer-scale; for example, the cumulative volume above 15 GPa exceeds 5 μm³ despite the non-uniform bending stress field.

Reviewer #2:

General comment: *The revised manuscript addresses most of the reviewer's comments; however, several critical issues remain unresolved:*

Response: Thank you for your careful re-evaluation of our revised manuscript. We appreciate your recognition that most of the previous comments have been addressed, and we are grateful for the opportunity to further improve the paper by responding to the remaining issues in detail below.

Comment 1:

1. Tensile testing methodology.

In the tensile experiments, fracture reproducibly initiates at the grip edge rather than within the gauge section. This behavior suggests that the specimen geometry is not optimal and should be modified, for example by increasing the gauge width to reduce stress concentration at the grips. Under the current configuration, it is questionable whether the tensile results can reliably represent the intrinsic mechanical properties of the TiB_2 ceramic. Accordingly, the statement that "the peak von Mises stress at the grip edge reaches ~65.9 GPa immediately prior to fracture" is disputable. In fact, the specimen sustains a nominal tensile stress of only ~13 GPa before fracture, which is significantly lower than the stress measured in bending tests (>30 GPa, Fig. 3).

Response: Thank you for this important comment. **We respectfully clarify that we do not use the present microscale tensile tests to measure the intrinsic uniaxial tensile fracture strength of the TiB_2 gauge section, nor do we claim that these tests directly represent the full intrinsic mechanical properties of TiB_2.** Rather, the purpose of these experiments is more specific: to test whether a truly micrometer-scale TiB_2 gauge volume can sustain very high, nearly uniform tensile stress without failing in the gauge itself. The answer is yes. In all three tensile specimens, fracture initiated reproducibly at the grip edge rather than within the calibrated gauge section, while the gauge section remained intact up to a nearly uniform tensile stress of ~13 GPa over a volume exceeding 4 μm^3. Therefore, **the tensile results provide**

direct evidence that the gauge section is not the failure-limiting site in the present setup and, importantly, support our central conclusion: the in-situ-grown large TiB_2 is essentially free of critical strength-limiting defects at the micrometer scale.

In the present hard-contact configuration, failure is governed by a severe local stress concentration at the diamond-gripper/TiB_2 contact, further aggravated by the slight but unavoidable misalignment between the two gripping faces at this length scale. Importantly, this grip-controlled failure remained dominant in all three specimens, despite two mitigation attempts, namely narrowing the gauge width (specimen T-2) and reinforcing the grip section with a deposited W layer (specimen T-3). These results support a broader conclusion: **for brittle specimens combining truly micrometer-scale gauge volumes with tens-of-gigapascal strength levels, the limiting factor in the current microscale tensile testing is the grip condition rather than the tensile strength of the gauge section itself**.

More specifically, the large force required to load such a strong micrometer-scale specimen, together with the hard contact between the diamond gripper and the TiB_2 grip section, inevitably generates a severe local stress concentration at the grip edge. **This interpretation is also consistent with the existing literature**: microscale tensile testing is generally successful for ductile materials, where local grip-induced stress concentrations can be relaxed by plastic deformation, whereas for brittle materials the reported successful tensile tests are typically confined to much smaller gauge volumes than those investigated here. To our knowledge, a direct tensile fracture measurement on a brittle ceramic combining both near-theoretical strength level and a truly micrometer-scale gauge volume has not yet been achieved. In this sense, the remaining obstacle is not primarily the TiB_2 gauge itself, but the current methodological limitations of microscale loading, especially under tensile testing of brittle, ultra-strong, truly micrometer-scale specimens. We therefore believe that future progress will likely require advances in micromechanics instrumentation and mechanical design—especially in new gripping/contact design and alignment control—rather than only incremental changes to specimen geometry.

We also respectfully suggest that increasing the gauge width would be unlikely to resolve this issue. In our experiments, the three specimens sustained similar maximum loads despite their different gauge cross-sectional areas, indicating that the attainable load was primarily capped by the grip condition. Under such circumstances, **enlarging the gauge cross-section would reduce, rather than increase, the nominal gauge stress reached prior to grip failure**. This is why our attempted optimization went in the opposite direction, namely reducing the gauge width in T-2 so as to increase the gauge stress relative to the grip-edge stress concentration. However, even this approach was insufficient to overcome the dominant grip effect. These results indicate that, within the current microscale tensile setup, the controlling limitation lies in the gripper/specimen contact condition, not in insufficient strength of the TiB_2 gauge section.

We further agree that the previously quoted ~65.9 GPa value should not be interpreted as an intrinsic strength value, nor compared directly with the >30 GPa values obtained from bending. In fact, the ~65.9 GPa quantity is solely a FEM-estimated local von Mises stress at the grip corner under a highly multiaxial, contact-dominated stress state, **introduced only to illustrate the severity of the grip-induced stress concentration**. By contrast, the >30 GPa values reported in bending are maximum principal tensile surface stresses at failure, obtained from specimen-specific FEM of bending specimens. **These are different stress measures under fundamentally different stress states and therefore are not directly comparable**. To avoid any possible misunderstanding, we have revised the main text to remove the explicit "~65.9 GPa" statement and now state only that FEM reveals a severe local multiaxial stress concentration at the grip edge.

What the tensile experiments do show is that the unbroken gauge section, with a large volume exceeding 4 μm^3, sustains a nearly uniform tensile stress of ~13 GPa without detectable cracking prior to grip-controlled failure. **We therefore treat ~13 GPa only as a conservative lower-bound tensile benchmark, not as the tensile fracture strength itself**. In other words, the result does not mean that the gauge section can sustain only ~13 GPa; rather, it means that the current setup can load the gauge only up to this level before premature grip-edge failure intervenes.

In this sense, **the tensile and bending experiments are complementary rather than contradictory: the tensile experiments demonstrate the defect-scarce nature of a large micrometer-scale TiB_2 gauge volume, whereas the bending experiments provide the measurements from which the fracture-stress level approaching the near-theoretical limit is extracted.**

Revisions in Main Text:

To avoid any possible misunderstanding, we have revised the manuscript in six places to make the role and interpretation of the microscale tensile tests explicit.

1) In the Abstract, we have further clarified the role of the tensile tests in relation to the bending results. Specifically, we now state explicitly that the bending-based experiments provide the primary assessment of the tensile-side fracture-stress level, whereas the direct tensile tests serve only as a conservative lower-bound tensile benchmark, because failure occurs at the grip edge rather than within the gauge section.

(Page 1, line 17)

…In-situ micromechanical bending tests on specimens with distinct geometries—including cantilevers, four-point bending beams, and C-shaped structures—combined with specimen-specific finite element analysis, consistently reveal tensile-side stresses in the tens of gigapascals……Microscale tensile experiments further show that a large gauge volume (~4 μm^3) can sustain very high, nearly uniform tensile stress of ~13 GPa without fracture before grip-edge failure, providing a conservative lower-bound tensile benchmark and indicating an exceptionally low population of strength-limiting defects within a micrometer-scale stressed volume…

2) In the last paragraph of the Introduction, we replaced "independent tensile benchmark" with "conservative lower-bound tensile benchmark" to define the role of the tensile tests more precisely.

(Page 3, line 23)

…these tests provide a conservative lower-bound tensile benchmark and support the conclusion that the in-situ grown TiB_2 is effectively free of strength-limiting defects at the micrometer scale…

3) In the Results and discussion, we removed the explicit "~65.9 GPa" statement from the main text and clarified that FEM reveals a severe local multiaxial stress concentration at the grip edge, whereas the gauge section sustains a nearly uniform tensile stress of ~13 GPa prior to grip-controlled failure. We also now state explicitly that these tensile tests do not provide a direct measurement of the intrinsic tensile fracture strength, but instead establish a conservative lower-bound tensile benchmark for a micrometer-scale TiB_2 gauge volume.

(Page 15, line 7)

…FEM analysis of the specimen-gripper assembly further reveals a severe local multiaxial stress concentration at the grip edge, while the gauge region sustains a nearly uniform tensile stress of ~13 GPa immediately prior to fracture (Fig. 6, Supplementary Fig. 15). Because the maximum load is similar across specimens, the uniform gauge stress decreases with increasing cross-sectional area. This dominant stress concentration could not be eliminated by either narrowing the gauge width (T-2) or reinforcing the gripped region via deposition of a protective tungsten layer over the grip section (T-3). Therefore, these microscale tensile tests do not provide a direct measurement of the intrinsic tensile fracture strength of TiB_2. Rather, they establish a conservative lower-bound tensile benchmark: the gauge section, with a volume exceeding 4 μm^3, remains intact up to ~13 GPa of near-uniform tensile stress prior to grip-controlled failure. This result shows that the present truly micrometer-scale TiB_2 volume can sustain very high tensile stress without detectable cracking, supporting an exceptionally low population of strength-limiting defects in our TiB_2…

4) In the Results and discussion, in the paragraph discussing Fig. 7b, we added a phrase after the description of the ~13 GPa value as a conservative lower bound, to avoid any misunderstanding of the role of the tensile result.

(Page 18, line 2)

…This value therefore provides a conservative lower bound, rather than the tensile fracture strength of the gauge section itself, and demonstrates that micrometer-scale TiB_2 volumes can sustain exceptionally high, near-uniform tensile stress without fracture…

5) In the Methods under "FEM analyses", we added a clarification that the reported ~13 GPa value represents the nearly uniform tensile stress sustained by the intact gauge section immediately before grip-edge failure, whereas any local grip-edge von Mises stress is used only to illustrate the severity of the contact-induced multiaxial stress concentration and is not compared directly with the bending-derived tensile fracture stresses.

(Page 23, line 6)

…For microscale tensile specimens, FE simulations incorporating the measured grip alignment were used to quantify the stress distribution and to identify the nearly uniform tensile stress sustained in the gauge section immediately prior to grip-edge failure (Supplementary Fig. 14). Accordingly, the tensile value reported (~13 GPa) is treated only as a conservative lower-bound tensile benchmark for the unbroken gauge section, rather than as the intrinsic tensile fracture strength of TiB_2. Any local von Mises stress at the grip edge is used solely to illustrate the severity of the grip-induced multiaxial stress concentration and is not compared directly with the bending-derived tensile fracture stresses…

6) We revised the Figure 6 caption to state explicitly that the microscale tensile tests provide a lower-bound tensile benchmark rather than a direct tensile-strength measurement.

(Page 16, line 12)

…In all three tests, FEM reveals a strong stress concentration at the clamp edge, consistent with the experimentally observed fracture location, while the gauge section remains crack-free up to the onset of clamp-edge failure. These tensile tests therefore provide a lower-bound tensile benchmark for the gauge section, rather than a direct measurement of intrinsic tensile fracture strength.

Comment 2:

2. Need for compression testing.

Given the uncertainties associated with the tensile results, compression testing is recommended. Compared with bending, compression provides a less complex stress state and would allow a more reliable assessment of the mechanical properties of the TiB_2 ceramic.

Response: Thank you for this constructive suggestion. Following your recommendation, **we performed additional in-situ compression tests on three newly prepared FIB-fabricated single-crystal TiB_2 micropillars** (P-1 to P-3), including one cuboid pillar and two cylindrical pillars, with specimen volumes of 1.1, 1.8, and 9.7 μm^3, respectively (Supplementary Figs. 17-18). The nominal peak compressive stresses (calculated as force divided by the initial cross-sectional area) reached 31 GPa for P-1, 43 GPa for P-2, and 33 GPa for P-3. Thus, **even under direct micropillar compression, the present micrometer-scale TiB_2 clearly sustains stresses in the tens-of-gigapascal regime**. Importantly, **these values agree well with the stress scale established by our previous experiments**, including the bending-derived tensile-side fracture stresses and the compressive-side stress level inferred from the C-shaped structures, all of which lie in the tens-of-gigapascal regime. In this sense, the new compression results provide an independent and fully consistent validation of the exceptional load-bearing capability of the in-situ-grown TiB_2 at the micrometer scale.

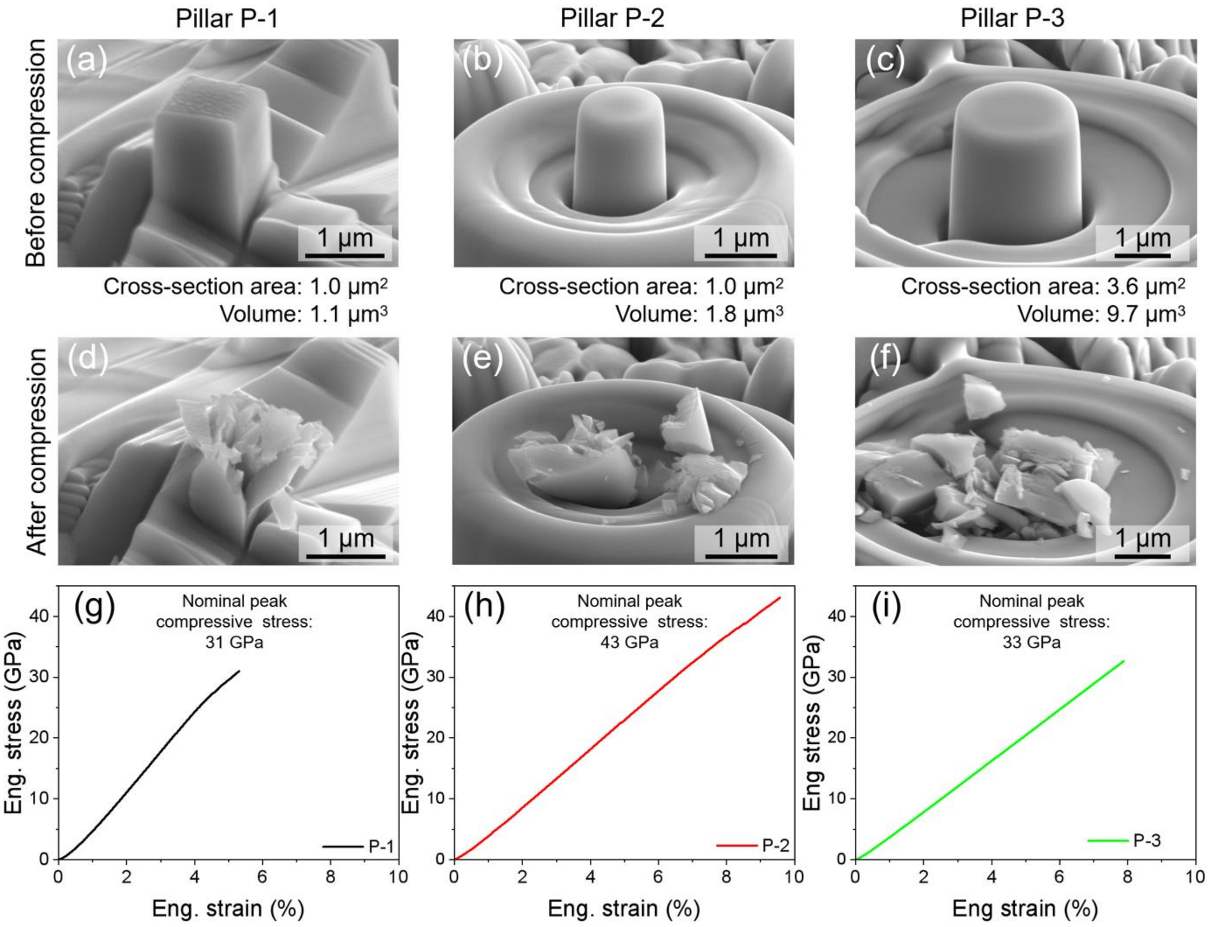

Supplementary Figure 18: Micropillar compression tests on micrometer-scale single-crystal TiB_2. (a-c) SEM images of the three FIB-fabricated TiB_2 micropillars, P-1 to P-3, prior to loading. P-1 is a cuboid pillar, whereas P-2 and P-3 are cylindrical pillars. (d-f) SEM images of P-1 to P-3 after catastrophic fracture following compression. (g-i) Engineering (nominal) stress-strain curves for P-1 to P-3, respectively. The nominal peak compressive stresses are 31 GPa for P-1, 43 GPa for P-2, and 33 GPa for P-3.

At the same time, we would like to clarify the role of compression in the present study. We agree that, compared with the grip-limited microscale tensile tests, compression provides a cleaner additional benchmark because it avoids the premature grip-edge failure encountered in tension. However, for brittle ceramics, compression is best viewed as a complementary validation rather than a replacement for the tensile/bending measurements that form the core of our work. The reason is that **brittle fracture is governed primarily by crack opening under tension, whereas compressive stress suppresses crack opening and is therefore**

intrinsically less sensitive to strength-limiting flaws. This is also why, throughout the manuscript, we focus on tensile-side stresses when estimating fracture strength and when discussing the intrinsic defect sensitivity of TiB_2.

Accordingly, we have added the micropillar compression results to the revised manuscript as a new complementary dataset. Their role is not to replace the tensile-side analyses, but to independently confirm that the present in-situ-grown micrometer-scale TiB_2 can sustain ultrahigh stresses in the tens-of-gigapascal range. Meanwhile, the bending-based tensile-side measurements remain essential for assessing the intrinsic fracture limit most relevant to brittle failure. Taken together, the compression and tensile-side results now provide a more complete and internally consistent understanding: the present TiB_2 combines exceptionally high stress tolerance at the micrometer scale with an exceedingly low population of strength-limiting defects.

Revisions in Main Text:

To further strengthen the revised manuscript, we revised four places to naturally incorporate the newly performed compression tests and clarify their role in the overall logic of the study: the bending tests remain the primary basis for evaluating the tensile-side fracture behavior most relevant to brittle failure, the tensile tests provide a conservative lower-bound tensile benchmark, and the compression tests offer independent support that the present micrometer-scale TiB_2 can indeed sustain stresses in the tens-of-gigapascal regime.

1) In the Abstract, we now state explicitly that the newly added micropillar compression results provide a complementary verification of the ultrahigh strength level indicated by the bending-derived results.

(Page 1, line 27)

…exceptionally low population of strength-limiting defects within a micrometer-scale stressed volume. Complementary micropillar compression tests, reaching nominal peak stresses in the tens of gigapascals, further support the ultrahigh strength level indicated by the bending results.

2) In the Introduction, we added a brief statement at the end of the last paragraph to clarify the role of the compression tests.

(Page 3, line 25)

…Finally, complementary micropillar compression tests reach nominal peak stresses in the tens of gigapascals, further confirming the ultrahigh stress-bearing capability of micrometer-scale TiB_2.

3) In the Results and discussion, we added several sentences following the paragraph discussing the tensile tests and serial FIB sectioning, to first present the newly performed micropillar compression results and then clarify that compression serves here as a complementary validation of the ultrahigh, tens-of-gigapascal stress-bearing capability of the present TiB_2.

(Page 15, line 28)

…As a further complementary check, we performed in-situ compression tests on three FIB-fabricated single-crystal TiB_2 micropillars (Supplementary Figs. 17-18), obtaining nominal peak compressive stresses of 31, 43 and 33 GPa for specimen volumes of 1.1, 1.8 and 9.7 μm^3, respectively. Although compressive loading suppresses crack opening and is intrinsically less sensitive to strength-limiting flaws than tensile loading in brittle solids, these results provide an independent validation that the present TiB_2 sustains stresses in the tens-of-gigapascal regime at the micrometer scale, consistent with the bending-based measurements.

4) In the Methods under "Fabrication of micromechanical TiB_2 specimens", we added the micropillars as a fifth specimen geometry and clarified how the three additional single-crystal TiB_2 compression pillars were prepared.

(Page 20, line 14)

To effectively vary the stress state and specimen volume for robust failure analysis, we

employed five complementary specimen geometries, including (i) micro-cantilever beams (B-1 to B-10), (ii) clamped-clamped (fixed-end) microscale four-point-bending beams (B-11 to B-15), (iii) C-shaped structures (C-1 and C-2), (iv) microscale tensile specimens (T-1 to T-3), and (v) microscale compression pillars (P-1 to P-3). Focused ion beam (FIB) milling was performed using a Ga+ ion source at 30 kV in a Helios 5UC SEM/FIB system. The region of interest was first isolated with an ion beam current of 2-21 nA. Rough milling was conducted at 300-700 pA, followed by fine milling at 100 pA to smooth surfaces and minimize ion-induced damage. For the compression tests, three additional single-crystal TiB_2 micropillars were fabricated from exposed TiB_2 particles, including one cuboid pillar (P-1) and two cylindrical pillars (P-2 and P-3). Schematics and geometric parameters for the cantilevers, four-point-bending beams, C-shaped structures, tensile specimens, and compression pillars are provided in Supplementary Figs. 2, 7, 11, 13, and 17, with dimensions summarized in Supplementary Tables 1-2, 4-8...

5) In the Methods under "In-situ micro-mechanical testing", we added the compression-testing procedure and the definition of engineering (nominal) compressive stress and strain.

(Page 21, line 23)

…For microscale compression tests, TiB_2 micropillars P-1 to P-3 were compressed using a diamond flat-punch indenter under displacement control. The nominal engineering compressive stress was calculated from the applied load divided by the initial pillar cross-sectional area, and the nominal engineering compressive strain was calculated from the indenter displacement divided by the initial pillar height. Representative pre-fracture and post-fracture SEM images, together with the corresponding nominal stress-strain curves, are provided in Supplementary Fig. 18.

Revisions in Supplementary Information:

The corresponding SEM images and the nominal stress-strain curves for the compression pillars have been added to Supplementary Fig. 18. Schematics and geometric parameters for the compression pillars are provided in Supplementary Fig. 17, with dimensions summarized

in Supplementary Table 8.

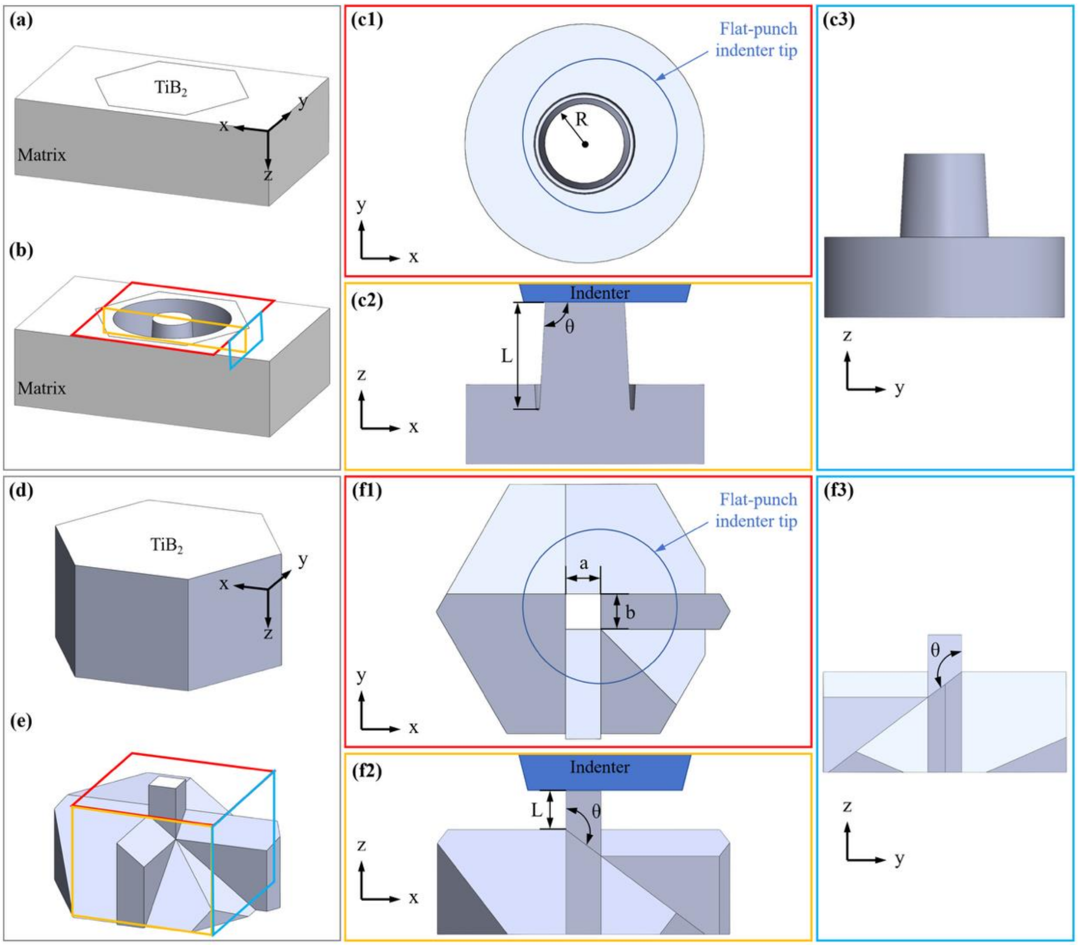

Supplementary Figure 17: Schematic of single-crystal TiB_2 particles embedded in a steel matrix and the resulting micropillar geometries after FIB milling. (a,b) Cylindrical micropillar and (d,e) cuboid micropillar. (c1-c3) Top, front, and side views of the cylindrical pillar shown in (b); (f1-f3) corresponding views of the cuboid pillar shown in (e).

Supplementary Table 8: Dimensions of FIB-milled micro-pillars, listing geometric parameters L to θ as defined in Supplementary Figure 17.

ID	L (μm)	a (μm)	b (μm)	R (μm)	θ (°)	Preparation method
P-1	1.10	1.00	1.00		127	Polishing (HMS-2)
P-2	1.81			1.14	92	Polishing (HMS-2)
P-3	2.68			2.14	92	Polishing (HMS-2)
